# Homeostatic and pathogenic roles of GM3 ganglioside molecular species in TLR4 signaling in obesity

**DOI:** 10.15252/embj.2019101732

**Published:** 2020-05-07

**Authors:** Hirotaka Kanoh, Takahiro Nitta, Shinji Go, Kei‐ichiro Inamori, Lucas Veillon, Wataru Nihei, Mayu Fujii, Kazuya Kabayama, Atsushi Shimoyama, Koichi Fukase, Umeharu Ohto, Toshiyuki Shimizu, Taku Watanabe, Hiroki Shindo, Sorama Aoki, Kenichi Sato, Mika Nagasaki, Yutaka Yatomi, Naoko Komura, Hiromune Ando, Hideharu Ishida, Makoto Kiso, Yoshihiro Natori, Yuichi Yoshimura, Asia Zonca, Anna Cattaneo, Marilena Letizia, Maria Ciampa, Laura Mauri, Alessandro Prinetti, Sandro Sonnino, Akemi Suzuki, Jin‐ichi Inokuchi

**Affiliations:** ^1^ Division of Glycopathology Institute of Molecular Biomembrane and Glycobiology Tohoku Medical and Pharmaceutical University Sendai Japan; ^2^ Department of Chemistry Graduate School of Science Osaka University Osaka Japan; ^3^ Graduate School of Pharmaceutical Sciences University of Tokyo Tokyo Japan; ^4^ Medical and Pharmaceutical Information Science Tohoku Medical and Pharmaceutical University Sendai Japan; ^5^ Department of Cardiovascular Medicine and Computational Diagnostic Radiology & Preventive Medicine Graduate School of Medicine The University of Tokyo Tokyo Japan; ^6^ Department of Clinical Laboratory Medicine Graduate School of Medicine The University of Tokyo Tokyo Japan; ^7^ Center for Highly Advanced Integration of Nano and Life Sciences (G‐CHAIN) Gifu University Gifu Japan; ^8^ Department of Applied Bio‐organic Chemistry Faculty of Applied Biological Sciences Gifu University Gifu Japan; ^9^ Organization for Research and Community Development Gifu University Gifu Japan; ^10^ Division of Organic and Pharmaceutical Chemistry Tohoku Medical and Pharmaceutical University Sendai Japan; ^11^ Department of Medical Biotechnology and Translational Medicine University of Milan Milano Italy; ^12^ Present address: Department of Pathophysiology and Metabolism Kawasaki Medical School Okayama Japan; ^13^ Present address: Department of Bioinformatics and Computational Biology The University of Texas MD Anderson Cancer Center Houston TX USA; ^14^ Present address: Nagasaki Clinic Tokyo Japan

**Keywords:** chronic inflammation, ganglioside GM3, inflammation amplification loop, obesity, TLR4, Immunology

## Abstract

Innate immune signaling via TLR4 plays critical roles in pathogenesis of metabolic disorders, but the contribution of different lipid species to metabolic disorders and inflammatory diseases is less clear. GM3 ganglioside in human serum is composed of a variety of fatty acids, including long‐chain (LCFA) and very‐long‐chain (VLCFA). Analysis of circulating levels of human serum GM3 species from patients at different stages of insulin resistance and chronic inflammation reveals that levels of VLCFA‐GM3 increase significantly in metabolic disorders, while LCFA‐GM3 serum levels decrease. Specific GM3 species also correlates with disease symptoms. VLCFA‐GM3 levels increase in the adipose tissue of obese mice, and this is blocked in TLR4‐mutant mice. In cultured monocytes, GM3 by itself has no effect on TLR4 activation; however, VLCFA‐GM3 synergistically and selectively enhances TLR4 activation by LPS/HMGB1, while LCFA‐GM3 and unsaturated VLCFA‐GM3 suppresses TLR4 activation. GM3 interacts with the extracellular region of TLR4/MD2 complex to modulate dimerization/oligomerization. Ligand‐molecular docking analysis supports that VLCFA‐GM3 and LCFA‐GM3 act as agonist and antagonist of TLR4 activity, respectively, by differentially binding to the hydrophobic pocket of MD2. Our findings suggest that VLCFA‐GM3 is a risk factor for TLR4‐mediated disease progression.

## Graphical abstract

Analysis of GM3 ganglioside composition in human serum under chronic inflammation conditions reveals that the fatty acid chain length of GM3 ganglioside impacts the inflammatory activation of macrophages via direct modulation of TLR4 signaling.

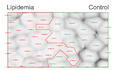

## Introduction

Chronic inflammation plays critical roles in pathogenesis of a variety of human diseases, including metabolic disorders (Lumeng, 2011; Hotamisligil, 2017). Prolonged and abnormal activation of pattern recognition receptors in innate immune system, such as Toll‐like receptors (TLR; Kawai & Akira, 2011; Moresco *et al*, 2011), causes chronic inflammation. In metabolic disorders, various ligands activate TLR4: (i) exogenous lipopolysaccharides elevated in serum (Cani *et al*, 2007), (ii) endogenous damage‐associated molecular patterns (DAMPs), *e.g*., high‐mobility group box‐1 protein (HMGB1; Harris *et al*, 2012; Guzmán‐Ruiz *et al*, 2014), free fatty acids (FFAs; Shi *et al*, 2006), and fetuin‐A protein (Pal *et al*, 2012), which are released from macrophages and adipose tissue. LPS and endogenous ligands induce production of various effector molecules including proinflammatory cytokines (*e.g*., tumor necrosis factor‐α [TNF‐α], interleukin‐6 [IL‐6]), which contributes to insulin resistance and dysregulation of lipid and energy metabolisms (Lumeng, 2011; Hotamisligil, 2017).

**Figure 1:**
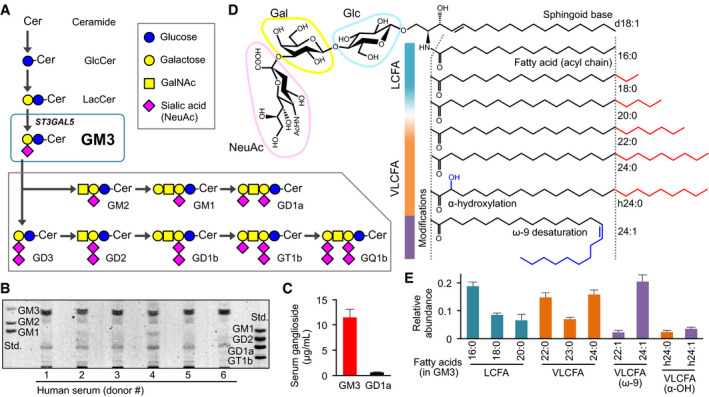
Molecular species of ganglioside GM3 in human serum and the acyl‐chain structures Biosynthetic pathway (schematic) of GM3, from ceramide, and to complex gangliosides.TLC analysis of ganglioside species in human serum.Quantification by densitometry of major ganglioside species GM3 and GD1a in human serum. Data expressed as mean ± SD, *n* = 6.Detailed structures of GM3 species: sialyllactose head group, sphingoid base (d18:1), typical fatty‐acid lengths (LCFA, VLCFA), and acyl‐chain modifications (α‐hydroxylation, ω‐9 desaturation).Quantification by LC‐MS/MS analysis of serum GM3 species with differing acyl‐chain structures. Total abundance of 10 species was defined as 1. Data expressed as mean ± SD, *n* = 6.

Gangliosides are important downstream metabolites of ceramide, a sphingolipid formed by an amide linkage between the sphingoid base and fatty acid (Bikman & Summers, 2011), and involved in a variety of cellular events (Inokuchi *et al*, 2018). Glycosyltransferases, UGCG and B4GALT5/6, convert ceramide into glucosylceramide (GlcCer) and lactosylceramide (LacCer), precursor glycosphingolipids (GSLs) for GM3 ganglioside. Consecutively, ST3GAL5, a GM3 synthase (GM3S), converts LacCer into GM3 by conjugating a sialic acid (N‐acetylneuraminic acid) (Fig 1A), which is followed by biosynthesis of complex gangliosides.

GM3 is secreted abundantly into human serum (Fig 1B), with concentration 10–15 μg/ml (~10 μM) (Fig 1C), and is circulated to all parts of the body, including insulin‐sensitive organs (*e.g*., liver, muscle, adipose; Senn *et al*, 1989; Veillon *et al*, 2015). Fatty acids of serum GM3 are composed of long‐chain fatty acid (LCFA), 16:0, 18:0, and 20:0; very‐long‐chain fatty acid (VLCFA), 22:0, 23:0, and 24:0; unsaturated VLCFA, 22:1 and 24:1; and α‐hydroxy VLCFA including h24:0 and h24:1 (Fig 1D), in almost same abundances of LCFA, VLCFA, and unsaturated VLCFA species, and a small amount of α‐hydroxy species (Fig 1E). Altered expression of various GM3 species has been observed in patients with metabolic disorders (Veillon *et al*, 2015); however, specific biological functions of these species are poorly understood.

On the other hand, it has been suggested that GM3 on the plasma membrane plays important roles in pathogenesis of metabolic disorders (Inokuchi *et al*, 2018). GM3 is also a major ganglioside in adipocytes, and its expression is induced by proinflammatory cytokines derived from adipose tissue macrophages (Tagami *et al*, 2002; Nagafuku *et al*, 2015; Wentworth *et al*, 2016). GM3 biosynthesis occurs in Golgi, and it subsequently becomes secreted into extracellular compartment or localized in plasma membrane as a component of membrane microdomains (also called “rafts”), which are signaling platforms comprised of sphingolipids (Lingwood & Simons, 2010). GM3 on plasma membrane affects diffusion kinetics of insulin receptors and regulates signal transduction (Kabayama *et al*, 2007); conversely, insulin signaling is restored when GM3 biosynthesis is blocked by glycosyltransferase inhibitors, *e.g*., D‐*threo‐*1‐phenyl‐2‐decanoylamino‐3‐morpholino‐1‐propanol (D‐PDMP) and Genz‐123346 (Tagami *et al*, 2002; Zhao *et al*, 2007). Knockout of *GM3S* diminishes not only systemic insulin resistance but also chronic inflammation in adipose tissue (Yamashita *et al*, 2003; Nagafuku *et al*, 2015), suggesting that GM3 might be involved in innate immune signaling upstream of insulin resistance; however, the molecular basis for such relationship remains unclear.

In this study, we investigated how serum GM3 species, carrying different acyl chains, regulate inflammatory signaling and contribute to onset of metabolic disorders. Here, we demonstrate that GM3 acts as an endogenous TLR4 modulator. VLCFA‐GM3 synergistically and selectively augmented TLR4 activation by LPS and HMGB1, and in contrast, LCFA and unsaturated VLCFA‐GM3 suppressed TLR4 activation. Serum VLCFA‐GM3 increased significantly and LCFA‐GM3 decreased sharply in metabolic disorders. Computational approaches using artificial intelligence revealed that specific GM3 species are related to clinical symptoms. VLCFA‐GM3 also increased in the adipose tissue of obese mice and the increase was attenuated in TLR4‐mutant mice, implying that TLR4 signaling itself is involved in production of VLCFA‐GM3. Our findings suggest that serum GM3 plays a role of rheostat for TLR4 signaling, and the increase in VLCFA‐GM3 is a risk factor for TLR4‐mediated disease progression.

## Results

### VLCFA‐GM3 species are involved in progression of chronic inflammation in metabolic disorders

To elucidate the role of GM3 species in pathophysiology of metabolic disorders, we analyzed expression patterns of serum GM3 species in human subjects (Veillon *et al*, 2015; Appendix Fig S1A–I). Sera were collected from human subjects, representing five pathological phases: healthy subjects (control, *n* = 24), visceral fat accumulation (VFA, *n* = 38) in presymptomatic phase, VFA with dyslipidemia (lipidemia, *n* = 28), VFA with hyperglycemia (glycemia, *n* = 15), and VFA with dyslipidemia and hyperglycemia (lipidemia + glycemia, *n* = 17). Scores of homeostatic model assessment for insulin resistance (HOMA‐IR) and serum C‐reactive protein (CRP) were evaluated as indicators of insulin resistance and chronic inflammation, respectively. HOMA‐IR and CRP displayed significant correlation with each other (Appendix Fig S1J), and a gradual increase in the order: control < VFA < lipidemia < glycemia < lipidemia + glycemia (Appendix Fig S1K and L). These findings indicate that the order of the five phases corresponds to increasing severity of insulin resistance and chronic inflammation.

**Figure 2:**
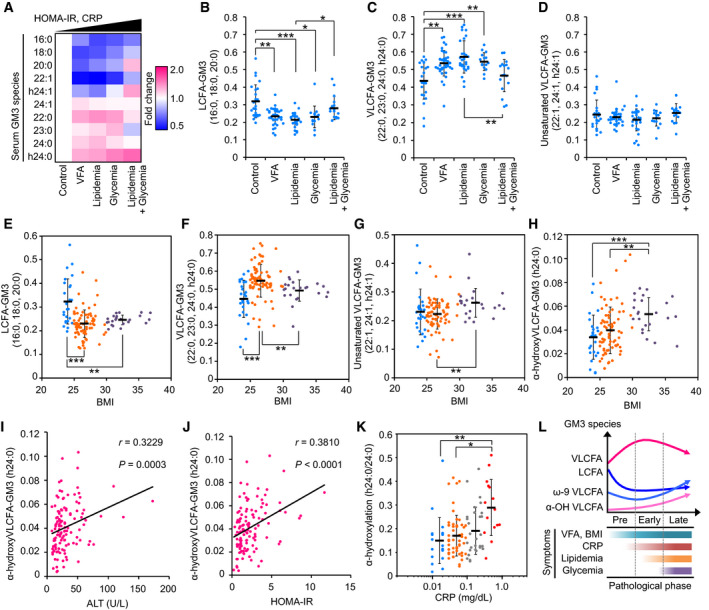
Alterations of relative abundance of GM3 species are involved in disease progression and chronic inflammation AHeat map analysis of serum GM3 species in various pathological phases: control (*n* = 24), VFA (*n* = 38), lipidemia (*n* = 28), glycemia (*n* = 15), and lipidemia + glycemia (*n* = 17). Colors indicate fold change average of each species relative to control (defined as 1), as shown in key at right. Order of pathological phases corresponds to increments of HOMA‐IR and serum CRP.B–DProperties of various GM3 species as a function of pathological phases: control (*n* = 24), VFA (*n* = 38), lipidemia (*n* = 28), glycemia (*n* = 15), and lipidemia + glycemia (*n* = 17). Data shown are relative abundances of total LCFA species (16:0, 18:0, 20:0) (B), total VLCFA species (22:0, 23:0, 24:0, h24:0) (C), and total unsaturated VLCFA species (22:1, 24:1, h24:1) (D) relative to total of ten major GM3 species (defined as 1) in each subject.E–HProperties of various GM3 species as a function of BMI: LCFA‐GM3 (E), VLCFA‐GM3 (F), unsaturated VLCFA‐GM3 (G), and α‐hydroxy VLCFA‐GM3 (h24:0) (H). Colors indicate disease severity: light blue, no abnormal scores (*n* = 25); orange, early‐phase obesity (*n* = 74); purple, severe obesity (*n* = 23).I, JSpearman's correlations for GM3 h24:0 vs. ALT (I) and vs. HOMA‐IR (J).KPlots of α‐hydroxylation rate (h24:0/24:0) vs. serum CRP. Colors indicate range of CRP value (mg/dl): light blue, 0.01–0.02 (*n* = 21); orange, 0.03–0.09 (*n* = 56); gray, 0.10–0.29 (*n* = 29); red, 0.3–1.0 (diagnostically abnormal; *n* = 15).LAssociation between serum GM3 species and progression of metabolic disorders (schematic).Data information: Data shown are individual values and mean ± SD, analyzed by two‐tailed unpaired *t*‐test with Bonferroni's correction. *P < 0.05, **P < 0.01, and ***P < 0.001 for comparisons between indicated groups.

Circulating levels of serum GM3 species were evaluated by LC‐MS/MS analysis (Appendix Fig S2A–K). Heat map analysis, which summarizes properties of the ten major species, indicated progressive increase in VLCFA species and decrease in LCFA species in association with increases in HOMA‐IR and serum CRP (Fig 2A). LCFA species (16:0, 18:0, 20:0) decreased sharply in VFA, lipidemia, and glycemia (Fig 2B), whereas VLCFA species (22:0, 23:0, 24:0, h24:0) largely increased (Fig 2C). Unsaturated VLCFA species were mostly constant as total (Fig 2D); 22:1 and h24:1 decreased, but 24:1 slightly increased (Fig 2A). The ratio of total VLCFA species to total LCFA/ unsaturated VLCFA species increased notably in presymptomatic and early phases of metabolic disorders (Appendix Fig S2L).

**Figure EV1:**
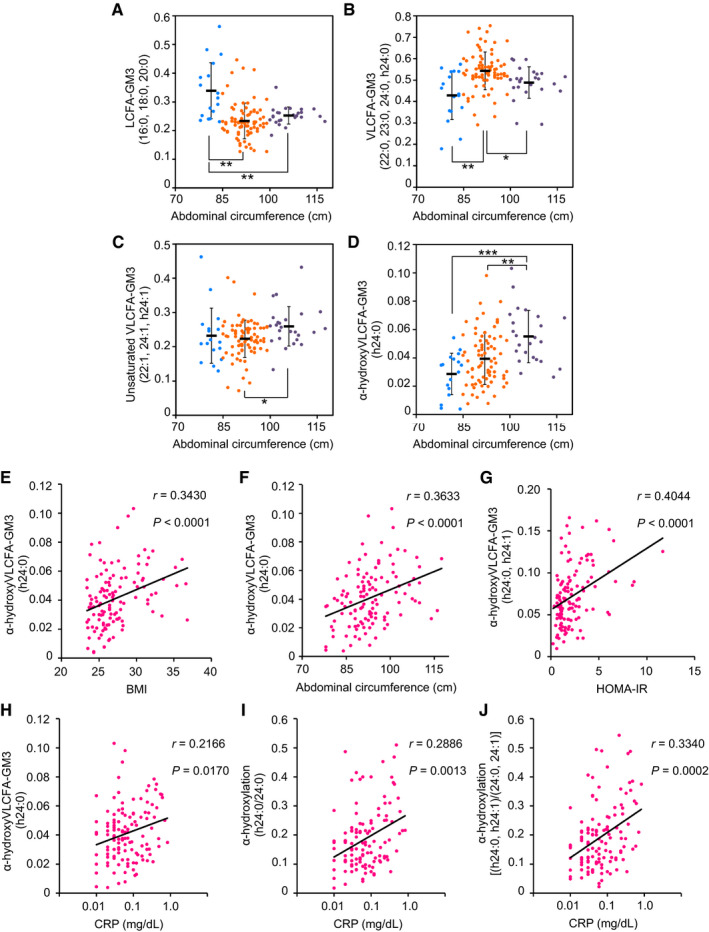
Properties of various GM3 species as a function of clinical markers of metabolic disorders and chronic inflammation A–DLCFA species (A), VLCFA species (B), unsaturated VLCFA species (C), and α‐hydroxy VLCFA‐GM3 (h24:0) (D). Colors indicate disease severity: light blue, no abnormal scores (*n* = 17); orange, early‐phase obesity (*n* = 80); purple, severe obesity (*n* = 25). Data shown are mean ± SD, analyzed by two‐tailed unpaired *t*‐test with Bonferroni's correction. *P < 0.05, **P < 0.01, and ***P < 0.001 for comparisons between indicated groups.E–JSpearman's correlations for GM3 h24:0 vs. BMI (E), GM3 h24:0 vs. abdominal circumference (F), total of α‐hydroxy GM3 (h24:0 and h24:1) vs. HOMA‐IR (G), GM3 h24:0 vs. serum CRP (H), α‐hydroxylation rate (h24:0 to 24:0) vs. serum CRP (I), and α‐hydroxylation rate (h24:0 and h24:1 to 24:0 and 24:1) vs. serum CRP (J).Data information: Sample sizes: (A–G), *n* = 122; (H‐J), *n* = 121.

Early‐phase increases in body mass index (BMI) (> 25) or abdominal circumference (> 85 cm) were associated with sharp reduction in LCFA species (Figs 2E and EV1A) and increase in VLCFA species (Figs 2F and EV1B). These findings suggest that increases in VLCFA‐GM3 species occur in obesity, and play a role in early pathogenesis of metabolic disorders. In cases of severe obesity (BMI > 30 and/or abdominal circumference > 100 cm) and severe metabolic disorders (lipidemia + glycemia), there was moderate reduction in VLCFA‐GM3 species (Figs 2F and EV1B) and significant increase in unsaturated species (Figs 2G and EV1C). These findings indicate that desaturation of VLCFA species occurs after onset of metabolic disorders.

Abundance of α‐hydroxy VLCFA‐GM3 (h24:0) showed a linear increase along with increases in BMI and abdominal circumference (Figs 2H and EV1D), with strong correlation (Fig EV1E and F). α‐hydroxy VLCFA‐GM3 was also strongly correlated with indicators of insulin resistance and chronic inflammation (ALT, HOMA‐IR, CRP) (Figs 2I and J, and EV1G–J). In particular, the ratio of h24:0 to 24:0 was much higher in subjects with abnormal CRP value (> 0.3 mg/dl) (Figs 2K and EV1I), indicating considerable involvement of h24:0 in chronic inflammation. Relationships between these GM3 species and pathophysiology of metabolic disorders are summarized schematically in Fig 2L. In steady state, homeostasis is maintained by balance of GM3 species; in presymptomatic and early phases, VLCFA species increase in correlation with chronic inflammation and insulin resistance; in late phases, modifications such as desaturation and α‐hydroxylation could occur in VLCFA species.

### Artificial intelligence‐based approaches revealed GM3 species specific to disease symptoms

**Figure 3:**
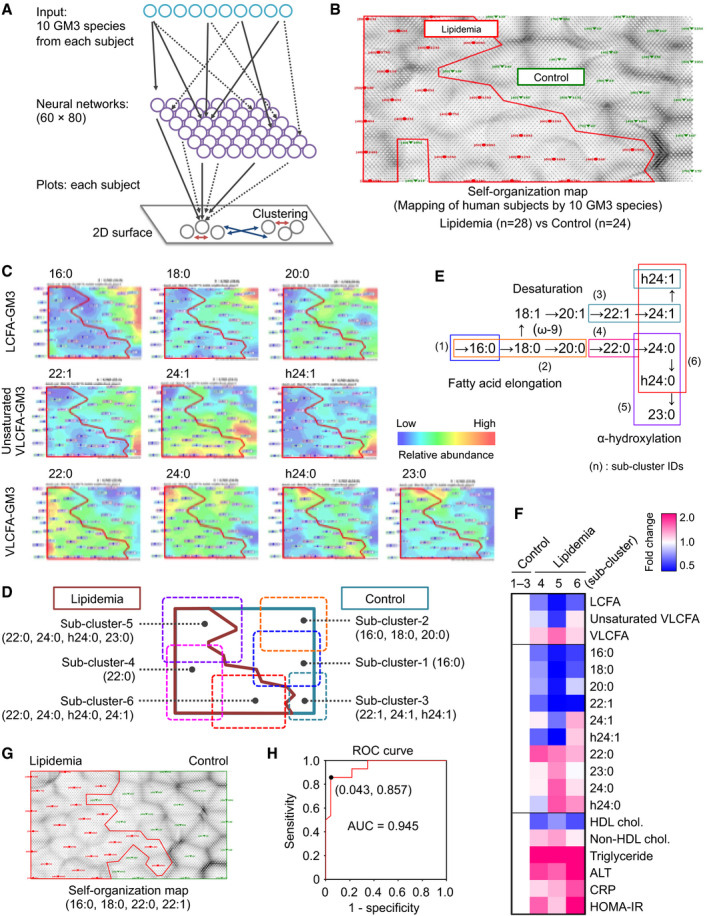
Self‐organization map (SOM) analysis based on relative abundances of serum GM3 species Procedure (schematic) for self‐organization map (SOM) analysis, a pattern recognition method using neural‐network‐type artificial intelligence. Complex patterns of multi‐dimensional information (in this case, expression patterns of the ten major GM3 species in human subjects) are mapped onto a 2D surface. Subjects having similar GM3 patterns are located proximal to each other and form several clusters (red arrows), whereas subjects having different GM3 patterns are located distal to each other (blue arrows).SOM analysis of control and lipidemia subjects based on expression patterns of ten GM3 species.Mapping of expression levels of ten GM3 species onto SOM in (B).Identification of sub‐clusters having different GM3 patterns based on SOM in (B).Metabolic pathways for fatty acids: elongation, desaturation, and α‐hydroxylation (α‐oxidation) (schematic). Sub‐clusters identified by SOM analysis are mapped on metabolic pathways.Heat map analysis for GM3 species and clinical markers of six clusters. Sample sizes: sub‐clusters 1–3 (total), *n* = 22; cluster 4, *n* = 7; cluster 5, *n* = 9; cluster 6, *n* = 12.Self‐organization map (SOM) based on four GM3 species as indicated at bottom.ROC curve derived from Bayesian regularized neural networks (BRNNs) based on four GM3 species in (G).

To analyze more detailed relationships between GM3 species and metabolic disorders, we utilized an unbiased approach using self‐organization map (SOM), a neural‐network‐type artificial intelligence model. Subjects were analyzed based on expression patterns of the ten major GM3 species, then mapped onto a two‐dimensional (2D) surface such that subjects with similar GM3 patterns are located near each other and form clusters (Fig 3A). SOM analysis gave nearly distinct clusters of control and lipidemia subjects (Fig 3B), indicating increases in VLCFA species and decreases in LCFA/ unsaturated VLCFA species in lipidemia subjects (Fig 3C). These subjects were further mapped into six sub‐clusters based on expression patterns of GM3 species: in control, sub‐clusters 1 (16:0), 2 (16:0, 18:0, 20:0), and 3 (22:1, 24:1, h24:1); in lipidemia, sub‐clusters 4 (22:0), 5 (22:0, 24:0, h24:0, 23:0), and 6 (22:0, 24:0, h24:0, 24:1) (Fig 3D). This classification indicates that elongation, α‐hydroxylation, and desaturation of GM3 acyl chains occur corresponding to disease progression (Fig 3E). Sub‐clusters 4 and 5 (higher in α‐hydroxy VLCFA) showed higher non‐HDL cholesterol; sub‐cluster 6 (higher in unsaturated VLCFA) showed higher CRP and HOMA‐IR (Fig 3F), indicating fatty‐acid modifications specific to different disease severities. Optimized SOM analysis, based on four species (16:0, 18:0, 22:0, and 22:1), was able to completely distinguish control vs. lipidemia subjects (Fig 3G). The receiver operating characteristic (ROC) curve gave excellent scores in sensitivity, specificity, and area under the curve (AUC; Fig 3H). These findings suggest that alterations of serum GM3 species are potential risks of disease progression, and the measurement is a valuable tool for clinical assessment.

### Serum GM3 species positively and negatively regulate innate immune responses in an acyl‐chain‐dependent manner

**Figure 4:**
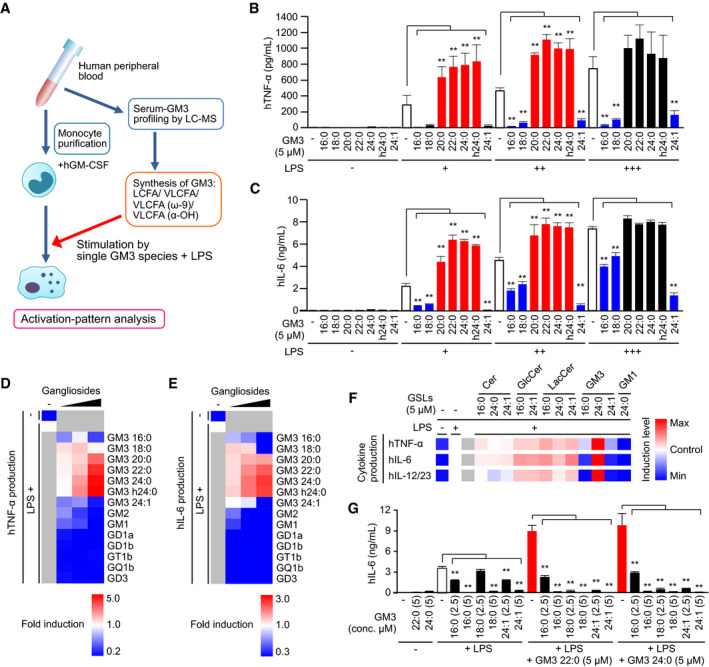
Positive and negative regulation of innate immune responses by serum GM3 species in an acyl‐chain‐dependent manner AProfiling of bioactivities of serum GM3 species in LPS‐mediated monocyte activation (schematic).B, CGM3‐mediated enhancement and inhibition of proinflammatory cytokine production from LPS‐stimulated monocytes (LPS: 0.06, 0.13, 0.25 ng/ml). TNF‐α (B) production and IL‐6 (C) production in culture supernatant were measured by ELISA.D, ECo‐stimulation of monocytes by LPS plus GM3 species or complex ganglioside species (1.5, 3.0, 4.5 μM). TNF‐α (D) production and IL‐6 (E) production were shown in heat maps.FCo‐stimulation of monocytes by LPS plus GM3 species or precursor GSL species. TNF‐α production, IL‐6 production, and IL‐12/23 production were shown in heat maps.GInhibitory effect of LCFA and unsaturated VLCFA‐GM3 on VLCFA‐GM3 species. IL‐6 production in culture supernatant was measured by ELISA.Data information: Data shown are mean ± SD (*n* = 3), analyzed by Tukey's multiple comparison test. **P < 0.01 for comparisons between indicated groups.

**Figure EV2:**
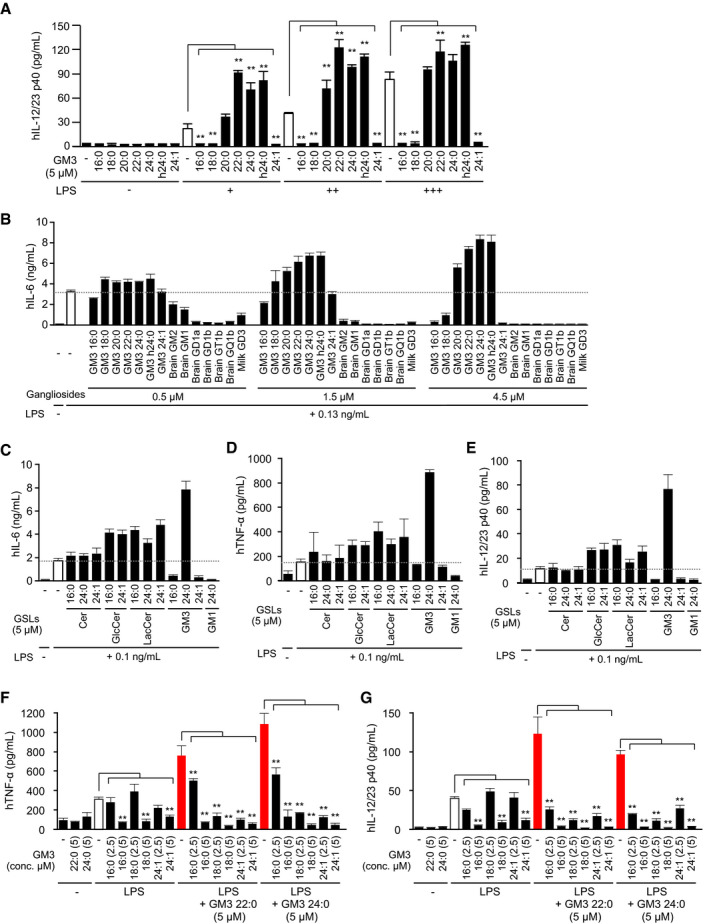
Positive and negative regulation of innate immune response by GM3 gangliosides AGM3‐mediated enhancement and inhibition of IL‐12/23 production from LPS‐stimulated monocytes (measured by ELISA).BCo‐stimulation of monocytes by LPS plus GM3 species or complex ganglioside species. IL‐6 production in culture supernatant was measured by ELISA.C–ECo‐stimulation of monocytes by LPS plus GM3 species or precursor GSL species. The production of IL‐6 (C), TNF‐α (D), and IL‐12/23 p40 (E) in culture supernatant was measured by ELISA.F, GInhibitory effect of LCFA and unsaturated VLCFA‐GM3 on VLCFA‐GM3 species. The production of TNF‐α (F) and IL‐12/23 p40 (G) in culture supernatant was measured by ELISA.Data information: Data shown are mean ± SD (*n* = 3) analyzed by Tukey's multiple comparison test. **P < 0.01 for comparison with LPS stimulation without GM3 species (A), or co‐stimulation by LPS and proinflammatory GM3 (22:0, 24:0) (F, G).

We investigated the effects of GM3 species (16:0, 18:0, 20:0, 22:0, 24:0, h24:0, and 24:1, in Fig 1D) on LPS‐mediated activation of human peripheral blood monocytes (Fig 4A). Every GM3 species by themselves did not exhibit notable effects, but VLCFA species (22:0, 24:0) and α‐hydroxy VLCFA species (h24:0) synergistically enhanced LPS‐mediated production of proinflammatory cytokines, *e.g*., IL‐6, TNF‐α, and IL‐12/23 p40 (Figs 4B and C, and EV2A). In contrast, LCFA species (16:0, 18:0) strongly inhibited LPS‐mediated cytokine production. Unsaturated VLCFA‐GM3 24:1 had an inhibitory effect. These enhancing and inhibitory effects were dose‐dependent and were observed at physiological concentration (< 10 μM) (Fig EV2B). These findings suggest that serum GM3 species positively and negatively regulate LPS‐mediated monocyte activation in an acyl‐chain‐dependent manner.

Among various types of gangliosides, only VLCFA‐GM3 species displayed dose‐dependent synergistic activation (Fig 4D and E), and other complex gangliosides exhibited inhibitory effects as reported (Shen *et al*, 2008). Monocyte activation was moderately enhanced in the presence of precursor GSL species and reached to the maximum in the presence of GM3 24:0 (Figs 4F and EV2C–E). GM3 species showed both positive and negative regulations in an acyl‐chain structure‐dependent manner, but Cer, GlcCer, and LacCer did not show such effects (Figs 4F and EV2C–E). Increasing doses of GM3 16:0, 18:0, and 24:1 reversed the effect of GM3 22:0 and 24:0 (Figs 4G and EV2F and G), suggesting that activation of human monocytes is regulated by the balance of LCFA‐GM3, VLCFA‐GM3, and unsaturated VLCFA‐GM3 species in serum.

### VLCFA‐GM3 species selectively enhance human TLR4/MD‐2 activation

**Figure 5:**
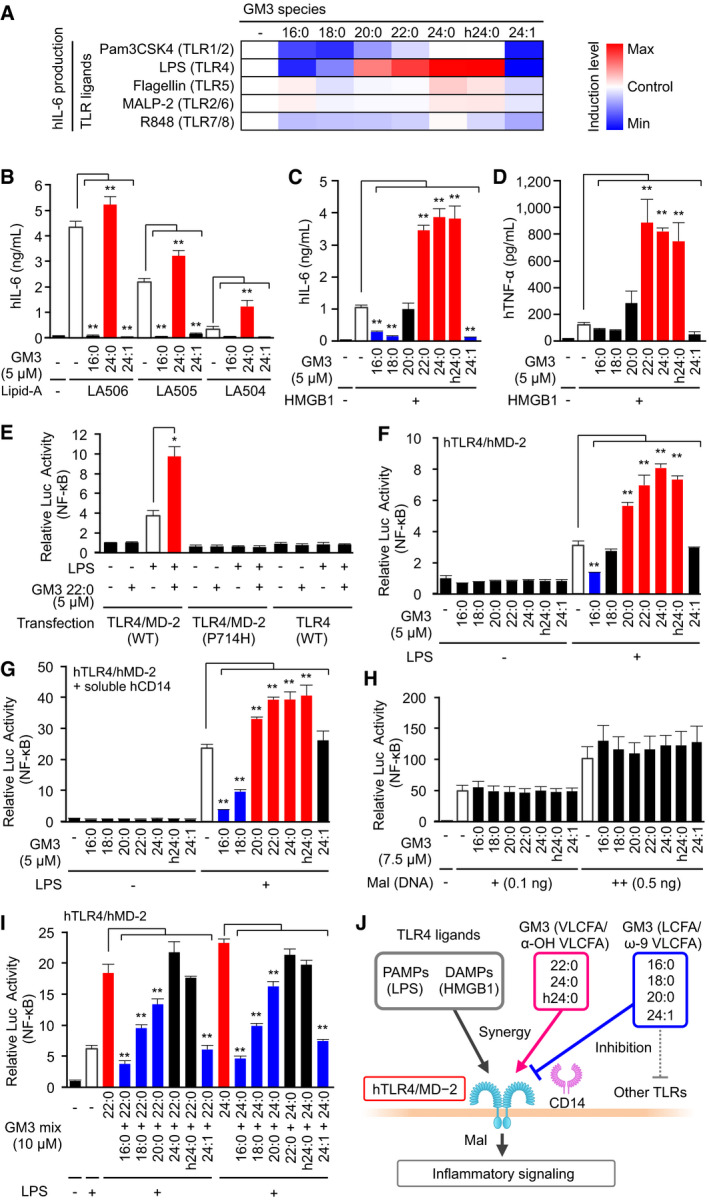
VLCFA‐GM3 species synergistically and selectively control human TLR4/MD‐2 activation ACo‐stimulation of human monocytes by GM3 species plus various TLR ligands: LPS (0.13 ng/ml), TLR4/MD‐2, Pam3CSK4 (0.5 μg/ml), TLR1/2, Flagellin (50 ng/ml), TLR5, R848 (0.5 μg/ml), TLR7/8, MALP‐2 (1.0 ng/ml), and TLR2/6. IL‐6 production in culture supernatant was quantified by ELISA (shown in a heat map).BCo‐stimulation of monocytes by GM3 species (16:0, 24:0, 24:1) plus synthetic TLR4 ligands LA506 (15 ng/ml), LA505 (150 ng/ml), or LA504 (150 ng/ml).C, DProduction of IL‐6 (C) and TNF‐α (D) in culture supernatant following co‐stimulation of monocytes by GM3 species plus human HMGB1.EOverexpression of hTLR4, hTLR4/hMD‐2, and hTLR4 (P714H) /MD‐2 in HEK293T cells, and co‐stimulation by GM3 22:0 with LPS (5 ng/ml). TLR4 activation was monitored by NF‐κB luciferase reporter assay (termed “Relative Luc Activity” on y‐axis).F, GCo‐stimulation of hTLR4/hMD‐2 by GM3 species plus LPS (5 ng/ml) (F) and further addition of soluble human CD14 (1 μg/ml) (G).HStimulation of Mal‐overexpressing HEK293T cells by GM3 species.ICo‐stimulation of hTLR4/hMD‐2 by LPS (5 ng/ml) plus various mixtures of GM3 species.JRegulation of hTLR4/hMD‐2 by GM3 species balance (schematic).Data information: Data shown are mean ± SD (A–D and F‐I, *n* = 3; E, *n* = 4) analyzed by Tukey's multiple comparison test. *P < 0.05 and **P < 0.01 for comparisons between indicated groups.

**Figure EV3:**
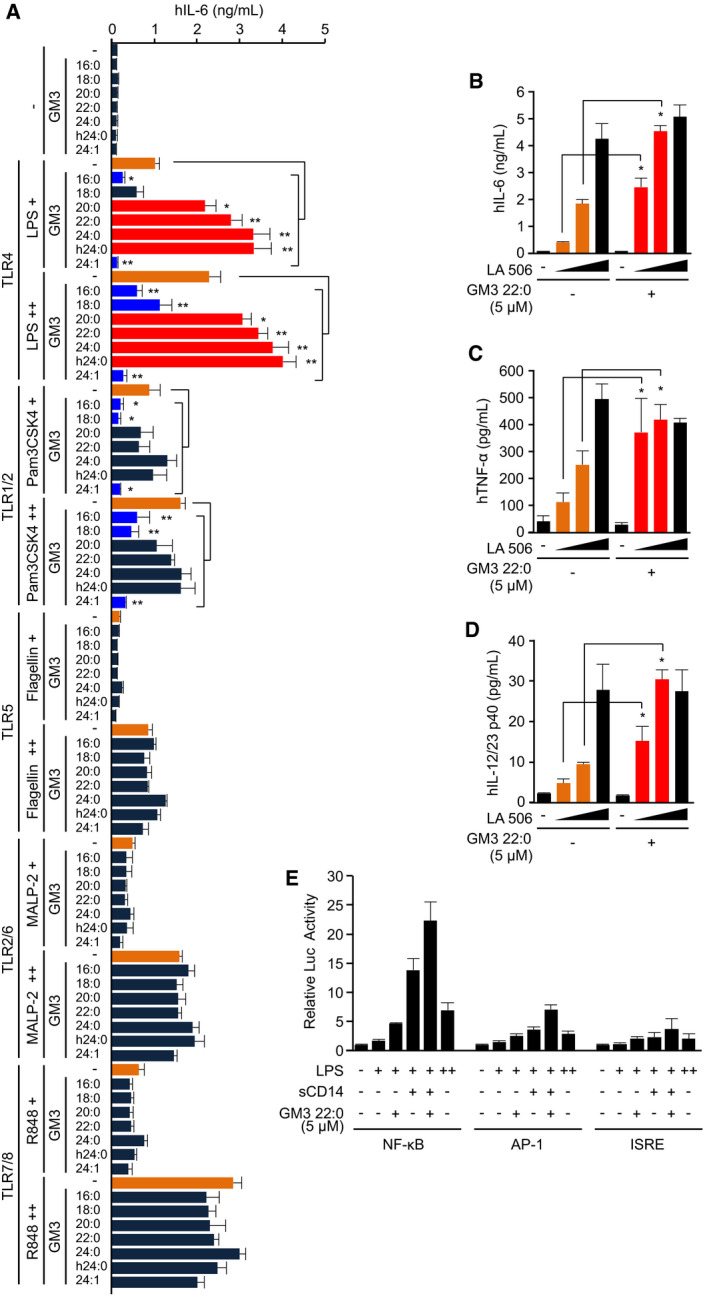
VLCFA‐GM3 species synergistically and selectively control human TLR4/MD‐2 activation ACo‐stimulation of human monocytes by GM3 species plus various TLR ligands: LPS (0.13, 0.25 ng/ml), TLR4/MD‐2, Pam3CSK4 (0.25, 0.5 μg/ml), TLR1/2, Flagellin (10, 50 ng/ml), TLR5, R848 (0.25, 0.5 μg/ml), TLR7/8, MALP‐2 (0.5, 1.0 ng/ml), and TLR2/6. IL‐6 production in culture supernatant was quantified by ELISA.B–DProduction of IL‐6 (B), TNF‐α (C), and IL‐12/23 p40 (D) in culture supernatant following co‐stimulation of monocytes by GM3 species plus LA506 (synthetic TLR4 ligand) (3, 10, 30 ng/ml).ERelative luciferase reporter activities of NF‐κB, AP‐1, and ISRE in response to LPS (+, 5 ng/ml; ++, 1 μg/ml), sCD14 (1 μg/ml), GM3 22:0 (5 μM), and their combinations. Relative luciferase activity of control condition was defined as 1 for every reporter gene.Data information: Data shown are mean ± SD (*n* = 3, A–D; *n* = 4, E) analyzed by Tukey's multiple comparison test (A) or by two‐tailed unpaired *t*‐test (B–D). *P < 0.05 and **P < 0.01 for comparisons between indicated groups.

To elucidate the molecular mechanisms underlying GM3‐mediated monocyte activation, we screened signaling pathways targeted by GM3 species. Monocytes were co‐stimulated by GM3 species in combination with various PAMPs, including ligands for TLR1/2, TLR4, TLR5, TLR2/6, and TLR7/8. We found that VLCFA species selectively synergized with LPS, a TLR4 ligand, but not for other TLR ligands (Figs 5A and EV3A). LCFA species strongly inhibited cytokine production by LPS, and partially by Pam3CSK4, a TLR1/2 ligand, but not by MALP‐2, a TLR2/6 ligand. Thus, GM3 species act as endogenous modulators selective for TLR4 signaling. GM3‐mediated modulations were also observed in di‐/monophosphoryl lipid A species LA506, LA505, and LA504, core structural components of LPS (Fig 5B). VLCFA‐GM3 22:0 significantly enhanced cytokine production in the presence of low‐dose LA506 (Fig EV3B–D). Moreover, VLCFA species strongly enhanced cytokine production by high‐mobility group box‐1 (HMGB1) protein (Fig 5C and D), an endogenous TLR2/4 ligand released from dead cells or visceral adipocytes in obese patients (Harris *et al*, 2012; Guzmán‐Ruiz *et al*, 2014). HMGB1‐mediated IL‐6 production was also inhibited by LCFA species (Fig 5C). These findings, taken together, indicate that GM3 species selectively modulate TLR4‐mediated cytokine production.

To confirm human TLR4 (hTLR4) as a target molecule of GM3 species, we reconstituted TLR4 signaling pathway in HEK293T cells. NF‐κB activity was not increased by LPS or GM3 22:0 in hTLR4 single‐expressing cells; however, synergistic enhancement was clearly observed in hTLR4/hMD‐2 co‐expressing cells (Fig 5E). Neither GM3‐mediated enhancement nor LPS‐mediated activation was observed in cells co‐expressing dominant‐negative hTLR4 variant (P714H, in intracellular domain)/hMD‐2 (Fig 5E). These findings suggest that hTLR4/hMD‐2 complex and its downstream pathway are required for NF‐κB activation by GM3 species. Activation of hTLR4/hMD‐2 by LPS was strongly enhanced by GM3 22:0, 24:0, and h24:0, but inhibited by GM3 16:0 (Fig 5F). hTLR4/hMD‐2 activation enhanced by addition of recombinant soluble hCD14 was strongly suppressed by 16:0 and 18:0 (Fig 5G), consistent with observed inhibition of CD14‐positive human cells by long‐chain species. Among the downstream targets of TLR4, in addition to NF‐κB, the activator protein 1 (AP‐1) activity was moderately enhanced by GM3 22:0, but the interferon‐stimulated response element (ISRE) activity was enhanced only weakly (Fig EV3E). None of the GM3 species affected NF‐κB activation by overexpression of MyD88‐adaptor‐like (Mal), a proximal adaptor protein of hTLR4 (Fig 5H), indicating that GM3 species target hTLR4/hMD‐2 complex upstream of Mal. The result of co‐stimulation by LPS plus GM3 species in 1:1 mixture suggests that the balance of extracellular GM3 species controls activation patterns of hTLR4/hMD‐2 (Fig 5I and J). 16:0 consistently inhibited TLR4 activation even in the presence of 22:0 or 24:0; 18:0 and 20:0 substantially counteracted enhancement by 22:0 or 24:0; and 24:1 reduced TLR4 activation to basal level by LPS single stimulation (Fig 5I). These findings, taken together, indicate that TLR4 signaling is selectively modulated by balance of GM3 species (Fig 5J).

### VLCFA‐GM3 species selectively enhance mouse TLR4/MD‐2 signaling

**Figure 6:**
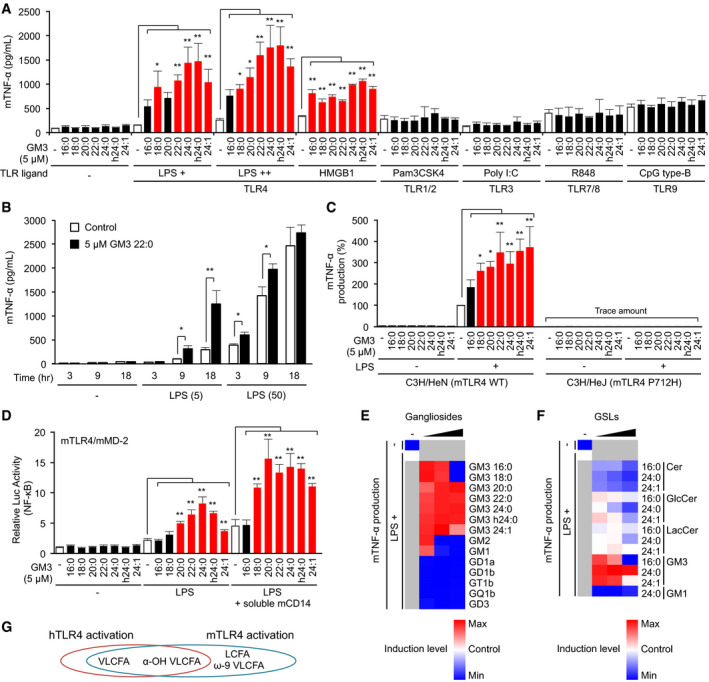
GM3 species selectively modulate mouse TLR4/MD‐2 signaling ACo‐stimulation of RAW macrophages by GM3 species plus various TLR ligands: LPS (0.5, 1.0 ng/ml), bovine thymus HMGB1 (0.25 μg/ml), Pam3CSK4 (50 ng/ml), Poly I:C (10 μg/ml), R848 (4 ng/ml), and CpG type B (20 nM). TNF‐α production in culture supernatant was quantified by ELISA.BCo‐stimulation of RAW macrophages by low‐ and high‐dose LPS (0, 5, 50 ng/ml) plus GM3 22:0 (5 μM). Time course of TNF‐α production in culture supernatant was quantified by ELISA.CCo‐stimulation of BMDMs from C3H/HeN or C3H/HeJ mice by GM3 species plus LPS (0.5 ng/ml), and TNF‐α production in culture supernatant.DCo‐stimulation of mTLR4/mMD‐2‐expressing HEK293T cells by GM3 species plus LPS (2.5 ng/ml), and further addition of soluble mouse CD14‐Fc fusion protein (1 μg/ml).E, FCo‐stimulation of BMDMs from C3H/HeN mice by LPS plus GM3 species and complex ganglioside species (E; 2.5, 5.0, 10 μM), or by LPS plus GM3 species and precursor GSL species (F; 2.5, 5.0, 10 μM) (shown in heat maps).GStructure–bioactivity relationships of GM3 species with human or mouse TLR4.Data information: Data shown are mean ± SD (A and B, *n* = 3; C, E, and F, *n* = 4; D, *n* = 6) analyzed by Tukey's multiple comparison test (A, C, and D) or two‐tailed unpaired *t*‐test (B). *P < 0.05 and **P < 0.01 for comparison with stimulation by TLR ligand without GM3 species.

We also investigated the effects of GM3 species on mouse TLR4/MD‐2 (mTLR4/mMD‐2). In RAW macrophages, VLCFA species strongly enhanced TNF‐α production by TLR4 ligands LPS and HMGB‐1 but not by other TLR ligands, similarly to results in human cells (Fig 6A and B). The enhancement was clearly observed in chronic/weak TLR4 activation by low‐dose LPS and was saturated in rapid/strong activation by high‐dose LPS (Fig 6B). LCFA‐GM3 and unsaturated VLCFA‐GM3 species moderately enhanced TLR4 activation (Fig 6A), in contrast to results in human cells. These effects were also observed in BMDMs from C3H/HeN (WT TLR4) mice, but not in C3H/HeJ (dominant‐negative TLR4, P712H) mice (Fig 6B), indicating that mTLR4 and its downstream signaling pathway are required. Activation patterns of macrophages were reproduced by overexpression of mTLR4/mMD‐2 complex in HEK293T cells (Fig 6C), and NF‐κB activity increased progressively associating with acyl‐chain length of GM3 species. Addition of soluble mouse CD14 enhanced the synergistic activation by GM3 species, indicating that CD14 facilitate GM3 representation to mTLR4/mMD‐2. Among complex gangliosides and precursor GSLs, synergistic activation in the mouse model was highest for VLCFA‐GM3 species (Fig 6D and E), similarly to findings in human cells. LCFA‐GM3 (16:0, 18:0) induced synergistic activation at physiological concentration to a similar degree as other GM3 species, but they displayed antagonistic effects at higher concentrations (Fig 6D). Molecular characteristics of GM3 species and effects on TLR4/MD‐2 are summarized in Fig 6F and Appendix Fig S3. VLCFA‐GM3 and LCFA‐GM3 displayed enhancement and inhibition for TLR4/MD‐2, respectively, correlating with volume of the hydrophobic moiety. Interestingly, lipid A/IVa is known to show agonistic and antagonistic activities correlating with total number of fatty acids, also corresponding to volume of the hydrophobic moiety (Akashi *et al*, 2001; Saitoh *et al*, 2004). Lipid IVa synergizes with LPS or lipid A in low‐dose range, but show antagonistic effects in high‐dose range (Mueller *et al*, 2004), similarly to LCFA‐GM3. These current and reported findings suggest that GM3 species utilize molecular mechanisms closely similar to lipid A/ IVa in regulating TLR4 activation.

### VLCFA‐GM3 species increase in mouse adipose tissue in metabolic disorders

**Figure EV4:**
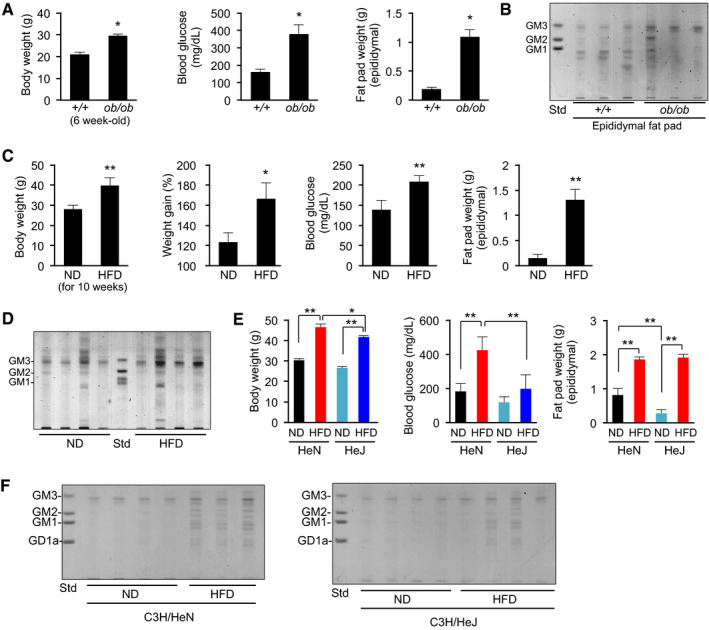
GM3 ganglioside in adipose tissue showed increased abundance in early‐phase obesity and short‐term HFD Body weight, blood glucose, and epididymal fat pad weight of 6‐week‐old control C57/BL6 mice and *ob/ob* mice (*n* = 3).Ganglioside species in epididymal fat pad were analyzed by TLC.Body weight, weight gain, blood glucose, and epididymal fat pad weight of normal diet (ND) and high‐fat diet (HFD) C57/BL6 mice (*n* = 4).Ganglioside species in epididymal fat pad were analyzed by TLC.Body weight, blood glucose, and epididymal fat pad weight of C3H/HeN mice (ND, HFD) and C3H/HeJ mice (ND, HFD) (*n* = 4).Full‐size images of Fig 7C. TLC analysis of acidic GSL fraction (equivalent to 0.1 mg protein) from epididymal fat pads of C3H/HeN and C3H/HeJ mice on ND or HFD for 8 weeks.Data information: Data shown are mean ± SD analyzed by two‐tailed unpaired *t*‐test (A, C) or by Tukey's multiple comparison test (E). *P < 0.05 and **P < 0.01 for comparisons between indicated groups.

**Figure 7:**
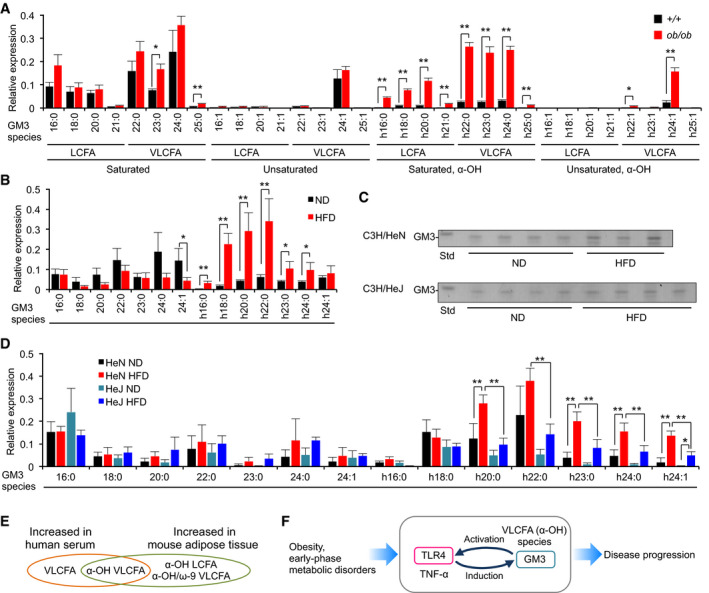
α‐hydroxy VLCFA‐GM3 species in adipose tissue showed increased abundance in obesity GM3 molecular species of 6‐week‐old control C57/BL6 mice and *ob/ob* mice were analyzed, respectively, by LC‐MS/MS (*n* = 3).GM3 molecular species of normal diet (ND) and high‐fat diet (HFD) C57/BL6 mice were analyzed by LC‐MS/MS (*n* = 4).TLC analysis of acidic GSL fraction (equivalent to 0.1 mg protein) from epididymal fat pads of C3H/HeN (A) and C3H/HeJ mice (B) on ND or HFD for 8 weeks.GM3 molecular species of C3H/HeN mice (ND, HFD) and C3H/HeJ mice (ND, HFD) were analyzed by LC‐MS/MS (*n* = 4).Comparison of increased GM3 species in human serum and mouse adipose tissue.Feedback loop mediated by TLR4 and GM3 species, promoting disease progression (schematic).Data information: Data shown are mean ± SD analyzed by two‐tailed unpaired *t*‐test (A, B) or by Tukey's multiple comparison test (D). *P < 0.05 and **P < 0.01 for comparisons between indicated groups.

In view of significant increase in VLCFA‐GM3 in early‐phase metabolic disorders in humans, we performed comparative studies using mouse models of obesity. Six‐week‐old *ob*/*ob* mice, showing early onset of metabolic disorders (Fig EV4A), had an increased abundance of GM3 in visceral adipose tissue (Fig EV4B). We analyzed these GM3 species by LC‐MS/MS. Control C57/BL6 mice had 16:0, 18:0, 20:0, 22:0, 23:0, 24:0, and 24:1 as major GM3 species, and a small amount of α‐hydroxy species (Fig 7A), showing similar composition to human serum GM3. On the other hand, *ob*/*ob* mice had notably increased levels of α‐hydroxy GM3: a strong increase in VLCFA species (h22:0, h23:0, h24:0), and moderate increase in LCFA and unsaturated VLCFA species (h16:0, h18:0, h20:0, h24:1). These findings suggest that increases in VLCFA‐GM3 and α‐hydroxylation occur in visceral adipose tissue in obesity and metabolic disorders.

Next, we analyzed diet‐induced obese mice as a more chronic and moderate model than *ob*/*ob* mice. High‐fat diet (HFD) in 8‐week‐old mice for 10 weeks resulted in obesity and increased GM3 levels (Fig EV4C and D). LC‐MS/MS analysis showed increase in α‐hydroxy GM3 species in HFD (Fig 7B); however, the predominant GM3 species in HFD mice were those with shorter acyl chains (h18:0, h20:0, h22:0) relative to *ob*/*ob* mice (h22:0, h23:0, h24:0). These findings indicate a correlation between the fatty‐acid length of α‐hydroxy GM3 and the severity of metabolic disorders.

We previously reported that proinflammatory cytokines released from adipose tissue‐resident macrophages induce GM3 production in adipocytes (Nagafuku *et al*, 2015). TLR4 is a key receptor for cytokine productions in adipose tissue (Shi *et al*, 2006; Suganami *et al*, 2017), implying that TLR4 activation itself induces increase in VLCFA‐GM3. So, we analyzed GM3 species in HFD C3H/HeN and C3H/HeJ mice by LC‐MS/MS. HFD in 8‐week‐old C3H/HeN mice for 8 weeks resulted in increased body weight, blood glucose level, and visceral adipose tissue weight (Fig EV4E). TLC analysis showed moderate increase in total GM3 (Figs 7C and EV4F), and LC‐MS/MS analysis revealed notable increases in α‐hydroxy VLCFA‐GM3 species in visceral adipose tissues of HFD C3H/HeN mice (Fig 7D). Diabetic phenotypes and increased levels of α‐hydroxy VLCFA‐GM3 species were ameliorated in HFD C3H/HeJ mice (Figs 7D and EV4E), suggesting that TLR4 signaling is partially involved in production of α‐hydroxy VLCFA species in obesity. These findings, taken together, suggest that α‐hydroxy VLCFA‐GM3 increases in both human serum and mouse adipose tissue (Fig 7E), and an interplay between TLR4 and GM3 species results in a feedback loop from TLR4 to GM3 (shown schematically in Fig 7F).

### GM3 species recognition by TLR4/MD‐2 induces receptor dimerization/ oligomerization

**Figure 8:**
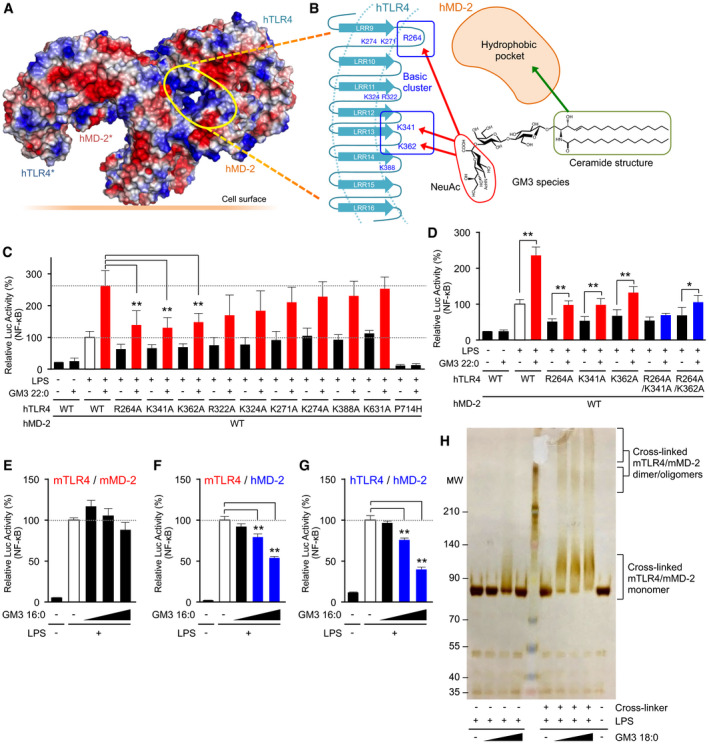
GM3 recognition by TLR4/MD‐2 induces receptor oligomerization A, BSurface electrostatic potentials of reported crystal structure of human TLR4/MD‐2/LPS complex (3FXI), and mapping of putative GM3‐binding pocket (A). Candidate basic residues and a hydrophobic pocket recognizing sialic acid and ceramide structure of GM3 are indicated (schematic) (B).C, DAlanine scanning for basic residues involved in signal transduction via VLCFA‐GM3 (*n* = 5) (C), and combinations of effective mutations (*n* = 6) (D). Signal transduction was monitored by NF‐κB reporter assay.E–GComparative inhibitory effects of GM3 16:0 on mTLR4/mMD‐2 (E), mTLR4/hMD‐2 (domain‐swapped complex) (F), and hTLR4/hMD‐2 (G) (*n* = 3).HCross‐linked SDS–PAGE analysis of recombinant mTLR4 (extracellular domain)/mMD‐2 complexed with GM3 18:0, GM3 species enhancing mTLR4 activation.Data information: Data shown are mean ± SD analyzed by Tukey's multiple comparison test. *P < 0.05, **P < 0.01 for comparisons between indicated groups.

To elucidate the molecular basis of GM3 recognition and signal transduction, we performed structure‐based mutation mapping on TLR4/MD‐2 complex. Previously reported crystal structures of TLR4/MD‐2 complex (Park *et al*, 2009; Ohto *et al*, 2012a) indicate that two ligand‐binding sites are formed on the dimerization interface between two TLR4/MD‐2 units (Fig 8A). MD‐2 forms hydrophobic pockets that bind to the acyl‐chain moiety of LPS, while TLR4 leucine‐rich repeats (LRRs) provide charged amino acids that recognize the hydrophilic head group of LPS (Fig 8B). Lys (K) and Arg (R) residues around the LPS‐binding pocket were replaced by Ala (A), because these cationic residues may recognize the sialic acid on GM3 saccharide chain. Mutations of R264, K341, and K362 greatly reduced synergistic hTLR4 activation by GM3 22:0 and partially reduced hTLR4 activation by LPS single stimulation (Fig 8C). R322, which recognizes a heptulose‐phosphate group on LPS oligosaccharide region (Park *et al*, 2009), contributed weakly to GM3‐mediated TLR4 activation (Fig 8C). Mutations of R264A, K341A, and K362A in combination effectively suppressed GM3‐mediated TLR4 activation (Fig 8D). On the other hand, nickel ion, an allosteric TLR4 ligand (Schmidt *et al*, 2010), did not display synergistic activation with GM3 22:0 (Appendix Fig S4). We also confirmed that R264A, K341A, and K362A had no effect on nickel‐mediated hTLR4 activation. These findings indicate that R264, K341, and K362 are required for hTLR4 activation by both LPS and GM3 species and facilitate their synergistic activation, whereas nickel ion does not synergize with GM3 species because its activity is independent of these amino acids.

To clarify MD‐2‐dependent recognition of GM3 acyl‐chain structure, we compared inhibitory effects of GM3 16:0 on mTLR4/mMD‐2, hTLR4/hMD‐2, and mTLR4/hMD‐2, a domain‐swapped complex comprised of mouse TLR4 and human MD‐2. mTLR4/mMD‐2 activation was not affected by 16:0 at physiological concentration (Fig 8E), whereas mTLR4/hMD‐2 activation and hTLR4/hMD‐2 activation were strongly inhibited by 16:0 (Fig 8F and G). Thus, MD‐2 evidently provides a basis for selectivity for GM3 species.

To investigate activation state of TLR4/MD‐2 complex induced by GM3, we performed chemical cross‐linking and SDS–PAGE analysis of recombinant mTLR4 extracellular domain/mMD‐2 complex. Addition of LPS, GM3 18:0, and chemical cross‐linker induced large mobility shift of mTLR4/mMD‐2 complex and observed molecular weights indicate the presence of dimers and higher‐order oligomers (Fig 8H). Previous reports indicate that LPS‐mediated signal transduction is initiated by dimerization of TLR4/MD‐2 unit (Akashi *et al*, 2001; Saitoh *et al*, 2004; Kobayashi *et al*, 2006). Clustering of TLR4 was observed by fluorescent and electron microscopy after LPS stimulation (Visintin *et al*, 2003; Triantafilou *et al*, 2004; Latty *et al*, 2018), and the signaling was mediated by a left‐handed helical oligomer of downstream adaptors consisting death domains (Lin *et al*, 2010); *i.e*., receptor oligomerization may provoke further downstream signaling. These previous and current findings, taken together, suggest that GM3 species act as TLR4‐selective endogenous modulators to induce receptor dimerization/ oligomerization, and consequently enhance signal transduction leading to chronic inflammation in metabolic disorders.

### Molecular docking approach implicates different binding modes of GM3 species modulating TLR4 activation

**Figure 9:**
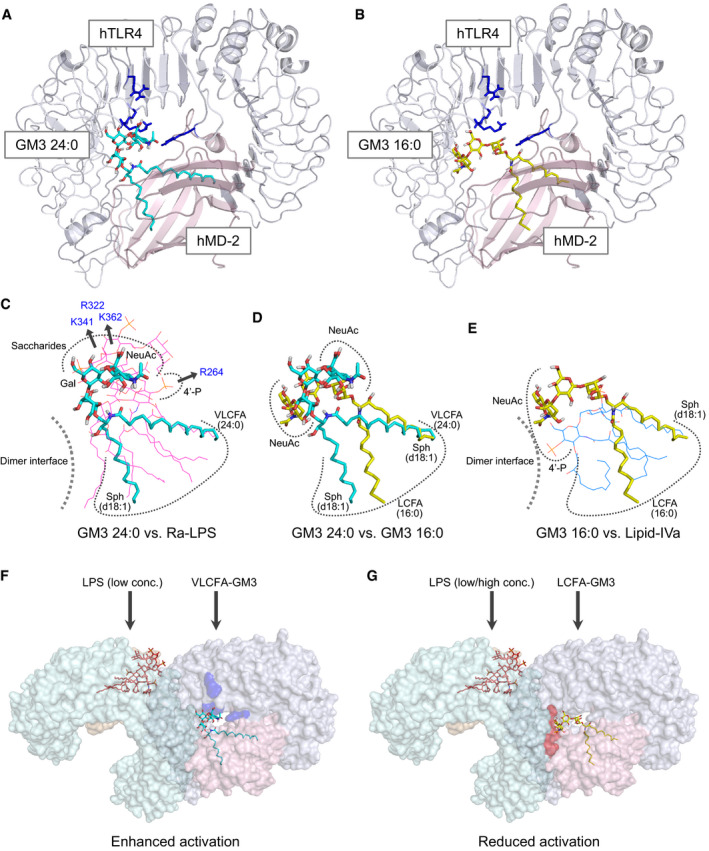
Ligand‐macromolecular docking analysis implicates species‐specific GM3 recognition by TLR4/MD‐2 A, BDocking model of GM3 24:0 (A) and 16:0 (B) binding to human TLR4/MD‐2 complex (3FXI). Basic residues of TLR4 are colored in blue.C–ESuperposition of GM3 24:0 (in docking model) vs. Ra‐LPS (in 3FXI) (C), GM3 24:0 vs. GM3 16:0 (in docking model) (D), and GM3 16:0 vs. lipid IVa (in 2E59) (E). Basic residues and the dimer interface are indicated schematically.F, GWorking model for hTLR4 activation enhanced by VLCFA‐GM3 species (F) and reduced by LCFA‐GM3 (G). Basic residues contributing to GM3 recognition are colored in blue. Residues of dimer interface are colored in red.

**Figure EV5:**
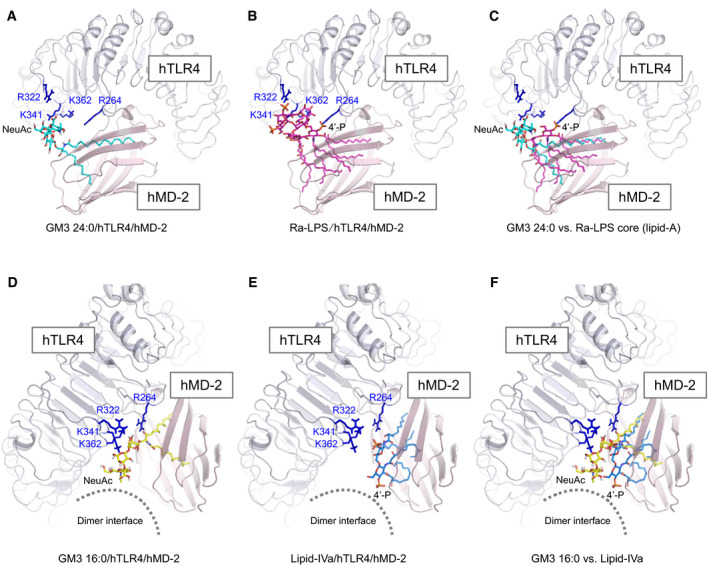
Binding model of VLCFA/LCFA‐GM3 species on hTLR4/hMD‐2 and the comparison to LPS and lipid IVa A–CDocking model of GM3 24:0 (A), Ra‐LPS (in 3FXI) (B), and superposition of GM3 24:0 vs. core structure of Ra‐LPS (lipid A) (C).D–FDocking model of GM3 16:0 (D), lipid IVa (in complex with hMD‐2 in 2E59) (E), and superposition of GM3 16:0 vs. lipid IVa (F). Basic residues of hTLR4 are colored in blue.

To figure out how GM3 species enhance and suppress TLR4 activation depending on the acyl‐chain structure, we performed a ligand‐macromolecular docking study on hTLR4/hMD‐2 complex. Binding modes of VLCFA‐GM3 (24:0) and LCFA‐GM3 (16:0) were sought on the molecular surface around the hydrophobic pocket of hMD‐2 and the basic residues of hTLR4. Docking models of hTLR4/hMD‐2/GM3 (24:0 or 16:0) complex are shown in Fig 9A and B. Similarly to LPS and lipid IVa, both GM3 24:0 and 16:0 bound the hydrophobic pocket of hMD‐2 via the fatty acid and the sphingoid base (Fig 9C–E). The binding model of GM3 24:0 overlapped closely to Ra‐LPS in the crystal structure of reference, and the saccharide chain of GM3 24:0 was surrounded by basic residues of TLR4 (Figs 9C and EV5A–C). The basic residues of TLR4 (K341, K362, and R322), that interact with the saccharide chain of LPS and show conformational changes upon TLR4 activation (Park *et al*, 2009; Ohto *et al*, 2012b), were closely associated with the saccharide chain of GM3 24:0. However, R264, a key residue recognizing 4′‐phosphate of LPS and triggering TLR4 activation (Park *et al*, 2009), was far from the saccharide chain of GM3 24:0. These results imply the underlying mechanism of VLCFA‐GM3 capability to enhance TLR4 signaling without triggering activation by itself. Since synergistic activation by VLCFA‐GM3 was mainly observed in the presence of low‐dose LPS or weak TLR4 ligands, VLCFA‐GM3 may act as an endogenous LPS mimic without intrinsic activity, which could sensitize TLR4 signaling by decreasing the ligand concentration required for TLR4 activation and increasing dimer/ oligomer formation (Fig 9F).

Next, binding model of GM3 16:0 was compared to lipid IVa in complex with hMD‐2 (Figs 9E and EV5D–F). Lipid IVa shows different binding mode in comparison with LPS, with reverse orientation of 4′‐phosphate and acyl chains, which may inhibit dimer formation of hTLR4/hMD‐2 by presenting hydrophilic groups (phosphate and glucosamine) to the lipophilic dimer interface (Park *et al*, 2009; Ohto *et al*, 2012a; Fig 9C and E). Similarly, GM3 16:0 showed opposite binding mode to GM3 24:0, with reverse orientation of the saccharide chain and the acyl chain (Fig 9D), which may interfere and reduce TLR4 activation through presentation of the saccharide chain to the dimer interface (Figs 9E and EV5D–F). On the other hand, lipid IVa and lipid A (LPS‐core structure) are known to show same binding orientations on mTLR4/mMD‐2 (Ohto *et al*, 2012a). Binding model of GM3 16:0 on mTLR4/mMD‐2 showed almost the same orientation compared with GM3 24:0 (Appendix Fig S5A–C). These comparative analyses implicate the mechanism by which GM3 species can enhance and reduce TLR4 activation in an acyl‐chain‐dependent manner.

## Discussion

TLR4 signaling plays crucial roles in pathogenesis of obesity and metabolic disorders. This study demonstrated that human TLR4/MD‐2 received positive regulation by VLCFA‐/α‐hydroxy VLCFA‐GM3 and negative regulation by LCFA‐/unsaturated VLCFA‐GM3 in the presence of LPS and HMGB1. LCFA‐GM3 species such as 16:0 consistently inhibited TLR4 activation even in the presence of VLCFA‐GM3 species 22:0 or 24:0; 18:0; and 20:0 (Fig 5I). These findings indicate that GM3 species function as a rheostat for TLR4 signaling (Fig 5J). Increases in VLCFA‐/α‐hydroxy VLCFA‐GM3 species, and decreases in LCFA‐GM3 species, were involved in pathogenesis of metabolic disorders via chronic inflammatory processes. Computational approaches revealed that elongation, α‐hydroxylation, and desaturation of fatty‐acid structures of GM3 were related to signatures of disease progression. α‐hydroxy VLCFA‐GM3 species were also increased in adipose tissue of obese mice. The increase in α‐hydroxy VLCFA‐GM3 was attenuated by TLR4 mutation, implying a feedback loop from TLR4 activation to GM3 production, analogous to that for free fatty acids (Suganami *et al*, 2007). GM3 induced dimerization/ oligomerization of TLR4/MD‐2, and MD‐2 was involved in recognition of the fatty‐acid structure of GM3. These findings suggest that GM3 plays an important role in TLR4 signaling, and the increase in VLCFA‐GM3 species, showing the strongest synergistic TLR4 activation, is a risk factor for TLR4‐mediated disease progression.

Measurement of serum GM3 species will potentially allow evaluation of hidden risks of TLR4‐signaling‐related inflammatory diseases (*e.g*., inflammatory bowel disease, chronic kidney disease, rheumatoid arthritis, cancer metastasis) via LPS and endogenous ligands such as HMGB1, S100A8/9 (Mrp8/14), and SAA3 (Vogl *et al*, 2007; Hiratsuka *et al*, 2008; Harris *et al*, 2012). Over 20 GM3 species, in addition to the ten major species examined in this study, are present in human serum (Veillon *et al*, 2015). Moreover, there is increasing evidence for important roles of GSLs in innate immune responses and chronic inflammation (Kondo *et al*, 2013; Nakayama *et al*, 2016; Nagata *et al*, 2017; Nitta *et al*, 2019). Expression pattern analysis utilizing artificial intelligence will allow us to deal effectively with the complexity and variety of GM3 and other GSL species, and to further elucidate the relationships between particular species and inflammatory diseases.

The detailed mechanism whereby GM3 species are secreted and presented to TLR4/MD‐2 complex is currently under study. It is supposed that GM3 is secreted as part of a lipoprotein complex (Senn *et al*, 1989; Veillon *et al*, 2015) allowing circulation from the liver to most body tissues, including adipose tissue. TLR4 has been shown to mediate innate immune responses by LDL cholesterol (Stewart *et al*, 2010). Ceramide 24:0 is preferentially incorporated into LDL cholesterol (Boon *et al*, 2013). The present study shows that levels of VLCFA‐GM3 and non‐HDL cholesterol increase together, whereas levels of LCFA‐GM3 and HDL cholesterol decrease together, indicating species‐selective incorporation into lipoproteins. However, other secretion pathways, such as exosomes and microvesicles (Skotland *et al*, 2017), may also be involved. Furthermore, α‐hydroxylation, mediated by enzymes such as fatty acid‐2 hydroxylase (FA2H), may contribute to secretion of GM3 species via reducing hydrophobicity and affecting lipid diffusion (Hama, 2010).

In regard to activation mechanisms, our results displayed that CD14 and MD‐2 facilitate GM3 to modulate TLR4 signaling. It is possible that CD14, MD‐2, and LPS‐binding protein take up serum GM3 species and transport them to TLR4, as reported for LPS (Ryu *et al*, 2017). As shown in docking study, VLCFA‐GM3 and LCFA‐GM3 may interact with TLR4/MD‐2 by utilizing different interaction modes to promote or disrupt dimerization, similarly to lipid A/IVa species and eritoran (a strong antagonist in lipid IVa analogs) (Mullarkey *et al*, 2003; Kim *et al*, 2007; Ohto *et al*, 2007, 2012a; Appendix Fig S3). In particular, molecular features of LCFA‐GM3 and unsaturated VLCFA‐GM3 resemble those of eritoran: (i) short aliphatic‐chain length (C10) in comparison with agonistic lipid A species (C14) and (ii) desaturation (C18:1, ω‐7) making a 180‐degree turn of the acyl chain in the hydrophobic pocket of MD‐2 (Kim *et al*, 2007). Mimetic compounds based on lipid A/IVa precursors (diacyl monosaccharide species), carrying less number of fatty acids, show antagonistic effect (Facchini *et al*, 2018). Thus, less fatty‐acid number, shorter acyl‐chain length, and desaturation may cooperatively contribute to antagonistic activity by affecting interaction mode. Our findings suggest that GM3 species modulate TLR4 activation by utilizing molecular mechanisms closely related to lipid A/ IVa. Formation of two ligand‐binding pockets on the dimerization interface between two TLR4/MD‐2 units has been suggested by crystallographic analyses (Park *et al*, 2009; Ohto *et al*, 2012a). Reported binding study of TLR4/MD‐2 with lipid A suggests that the maximal binding of the agonistic *E. coli* lipid A was approximately half‐fold lower than that of the antagonistic lipid IVa (Akashi *et al*, 2001; Saitoh *et al*, 2004); i.e., under physiological conditions, one ligand pocket is occupied by agonist (e.g., LPS) while the other is vacant or occupied by unknown intrinsic ligands. It may allow GM3 species to modulate dimerization efficiency via the second pocket (Fig 9F and G). Future studies are expected to reveal structures of oligomeric TLR4/MD‐2 signalosomes complexed with GM3 species. Additionally, it is known that the dimerization and internalization of mTLR4/MD‐2 upon acute stimulation by LPS can be analyzed by flow cytometry (Akashi *et al*, 2003; Zanoni *et al*, 2011; Tan *et al*, 2015), which might enable to detect GM3‐mediated receptor dynamics directly on the plasma membrane of living cells.

Biosynthesis of the various GM3 species may depend on several enzymes: fatty‐acid elongases (Elovls), acyl‐CoA desaturases, ceramide synthases (CerSs), and GM3S. Blocking of 16:0‐to‐18:0 fatty‐acid elongation in mice by *Elovl6* knockout was found to inhibit progression of metabolic disorders through alterations of fatty‐acid structures, *i.e*., increased 16:0 and decreased 18:0‐to‐24:0 levels (Matsuzaka *et al*, 2007). *Elovl6* deficiency therefore may attenuate increase in VLCFA‐GM3 species, and achieve homeostatic balance of acyl‐chain structures. On the other hand, increase in LCFA‐Cer (16:0) and decreases in VLCFA‐Cer (22:0, 24:0) in obese subjects, resulting from imbalance of CerS2/6 expression and inhibition of β‐oxidation, were reported to correlate to progression of metabolic disorders (Raichur *et al*, 2014; Turpin *et al*, 2014). Our results imply that such imbalances in Cer species might be involved in decreased production of LCFA‐GM3 and increased production of VLCFA‐GM3 in metabolic disorders. Fatty‐acid desaturation was shown to occur in the resolution phase of innate immune response, and to reduce inflammation (Oishi *et al*, 2017); however, the direct mechanism whereby ω‐9 mono‐unsaturated VLCFA attenuates chronic inflammation is not completely understood. Increased levels of unsaturated GM3 species in severe metabolic disorders may result from desaturation mechanisms after the activation phase. Both elongase and desaturase genes are regulated by SREBP‐1, a key transcription factor in lipid signaling whose activation occurs in parallel with that of NF‐κB (Matsuzaka *et al*, 2007; Oishi *et al*, 2017). We previously reported that proinflammatory cytokines TNF‐α and IL‐1β induce *GM3S* expression and GM3 production in adipocytes (Tagami *et al*, 2002; Nagafuku *et al*, 2015). In the present study, *TLR4* deficiency reduced production of α‐hydroxy VLCFA‐GM3 (Fig 7D), suggesting the involvement of TLR4 signaling in GM3 production. These previous and current findings indicate that fatty‐acid structures and total expression level of GM3 species are controlled by complex, coordinated mechanisms regulated by innate immune signaling, lipid signaling, and other cellular responses.

Moreover, it should be clarified directly in adipose tissue that GM3 species could mediate the adipocyte–macrophage communication in the future study. It would be important to specify the GM3 and other ganglioside species expressed in a specific type of cells, such as macrophages, pre‐adipocytes, and differentiated adipocytes, that are mixed in adipose tissue. While pre‐adipocytes/adipocytes predominantly express GM3, it is considered that human monocytes and mouse macrophages express GM3 and GM1/GD1a, respectively (Yohe *et al*, 2001; Tanabe *et al*, 2009). However, it remains unclear how ganglioside species and their acyl‐chain structures are different in a cell‐type‐specific manner in the intact adipose tissue. To characterize miscellaneous cells in adipose tissue, in vitro enzymatic digestion/fractionation and antibody‐based cell sorting are performed generally. On the other hand, our previous report suggested that GM3 expression in adipocytes was regulated by the co‐presence of the resident macrophages in adipose tissue (Nagafuku *et al*, 2015). It has been also known that the activation of GM3 synthase in monocyte/macrophages was easily occurred during culturing in vitro (Gracheva *et al*, 2007). Therefore, the specific method such as the imaging mass spectrometry for GM3 species should be established in order to analyze GM3 species directly in the intact adipose tissues without in vitro cell manipulation (Sugimoto *et al*, 2016).

In regard to potential therapeutic approaches, treatment with supplemental LCFA‐GM3 16:0 may inhibit systemic and local production of TNF‐α, IL‐6, and IL‐12/23 via TLR4, and in part via TLR2, driven by LPS and HMGB1. On the other hand, VLCFA‐GM3 24:0 could act as a booster for immunological adjuvants such as monophosphoryl lipid A species (LA505, LA504) and other synthetic TLR4 ligands (Wang *et al*, 2016; Chan *et al*, 2017; Okamoto *et al*, 2017). Utilization of naturally occurring GM3 species may prevent production of autoantibodies (Bowes *et al*, 2002).

In conclusion, our findings would help clarify the pathophysiological roles of serum/ adipose GM3 species in TLR4 signaling, and the complex interplay between glycosphingolipid metabolism and innate immune signaling in metabolic disorders.

## Materials and Methods

### Ceramide, GSLs, and complex gangliosides

Ceramide species (16:0, 24:0, 24:1) and GlcCer species (16:0, 24:1) were from Avanti Polar Lipids (Alabaster, AL, USA). GlcCer (24:0), LacCer (16:0, 24:0, 24:1), and GM3 (16:0, 18:0, 20:0, 22:0, 24:0, h24:0, 24:1) and GM1 (18:0) were synthesized as described previously (Murase *et al*, 1989; Mauri *et al*, 1999). GM2 (from brain of Tay‐Sachs disease patient) was from Matreya (State College, PA, USA). Brain GD1a, GD1b, and GT1b were from Sigma‐Aldrich (St. Louis, MO, USA). Brain GQ1b was from AdipoGen Life Sciences (San Diego, CA, USA). Milk GD3 was from Nagara Science Co. (Gifu, Japan). Ceramides, GlcCer, and LacCer species were dissolved at 1 mM in warmed DMSO. Gangliosides were dissolved at 0.5 mM concentration in warmed low‐glucose DMEM (Nacalai Tesque; Kyoto, Japan). Stock solutions were stored at −30°C and diluted with low‐glucose DMEM to 100 μM concentration prior to experiments.

### TLR ligands and recombinant proteins

Toll‐like receptors ligands and recombinant proteins were purchased from the following vendors: LPS from *E. coli* O111:B4 (Sigma‐Aldrich); human recombinant HMGB1, soluble form human CD14 (BioLegend; San Diego); bovine thymus HMGB1 (Chondrex; Redmond, WA, USA); soluble form mouse CD14‐Fc fusion (Sino Biological, Inc.; Beijing, China); Pam3CSK4 and MALP‐2 (Novus Biologicals; Littleton, CO, USA); and Poly I:C, R848, Flagellin from *Salmonella typhimurium*, and CpG (ODN 1826) (Enzo Life Sciences; Farmingdale, NY, USA). TLR ligands other than R848 were reconstituted in endotoxin‐free water (Nacalai Tesque). R848 was reconstituted in ethanol (Fujifilm Wako; Osaka, Japan). Lipid A and derivatives were previously synthesized (Imoto *et al*, 1985, 1987; Liu *et al*, 1999).

### Vector construction

Vector carrying mouse MD‐2 and TLR4 cDNA (pDUO‐mMD2/TLR4) was from InvivoGen (San Diego). cDNA fragments, fused with a KpnI site and one Kozak sequence (ACC) at 5′‐end and SalI site at 3′‐end, were amplified by PCR (KOD‐Plus‐Neo; Toyobo) and inserted separately into pCDNA3 at KpnI and XhoI sites (Invitrogen). A set of vectors for dual luciferase assay, NF‐κB reporter gene (pGL3‐ELAM; a firefly luciferase gene controlled by NF‐κB‐dependent promoter of ELAM‐1), control reporter gene (pRL‐TK; a Renilla luciferase gene controlled by constitutive active promoter of thymidine kinase), and pCDNA3 vectors carrying human MD‐2 and TLR4 cDNA were previously described (Muta & Takeshige, 2001; Fujimoto *et al*, 2004). Reporter vectors for AP‐1 and ISRE were purchased (Promega; Australia). Site‐directed mutagenesis was performed according to the manufacturer's protocol of QuikChange (Agilent; Santa Clara, CA, USA) with minor modifications.

### Purification and stimulation of human monocytes

Heparinized fresh human peripheral blood was diluted to 2× volume with cold (4°C) endotoxin‐free PBS (Nacalai Tesque) containing 1 μg/ml polymyxin B (Sigma‐Aldrich). Diluted blood was overlaid on cold (4°C) lymphocyte separation solution (Nacalai Tesque) containing 1 μg/ml polymyxin B and centrifuged at 800 *g* for 25 min at 4°C. Peripheral blood mononuclear cell (PBMC) fraction was collected and diluted to 2× volume of wash solution (PBS, 1% heat‐inactivated FCS (Biosera), 5 mM EDTA, pH 7.5 (Nacalai Tesque), 1 μg/ml polymyxin B). PBMCs were separated by centrifugation at 600 *g* for 10 min at 4°C, washed twice, resuspended in 750 μl wash solution and incubated with 120 μl anti‐human CD14 magnetic particles (BD Biosciences) for 30 min at room temperature. CD14‐positive cells (monocytes) were separated by magnetic field and washed 3× with wash solution. Purified cells were resuspended in cold low‐glucose DMEM with 0.75% FCS, left on ice for 45 min, counted, diluted to 2.0 × 10^5^ cells/ml with culture medium (low‐glucose DMEM, 0.75% FCS, 40 ng/ml recombinant human granulocyte‐macrophage colony‐stimulating factor (GM‐CSF) (BioLegend), and cultured in 96‐well plates (100 μl/well) overnight at 37°C under 5% CO_2_ atmosphere.

### Differentiation of mouse bone marrow‐derived macrophages (BMDMs)

Femoral and tibial bone marrows of 12‐ to 16‐week‐old nondiabetic C3H/HeN mice (Japan SLC Inc.) were collected in 1% FCS‐supplemented low‐glucose DMEM, and erythrocytes were lysed in RBC lysis buffer. Bone marrow cells were washed in 1% FCS DMEM and cultured 5–7 days in 10% FCS DMEM supplemented with 40 ng/ml recombinant human macrophage colony‐stimulating factor (M‐CSF) (BioLegend). Non‐adhesive cells were washed out with PBS. Differentiated macrophages were collected by scraping in ice‐cold PBS with 1% FCS/ 5 mM EDTA, washed, counted, diluted to 2.0 × 10^5^ cells/ml in 1% FCS DMEM, and cultured in 96‐well plates overnight at 37°C under 5% CO_2_ atmosphere.

### Cell culture and transfection

HEK293T cells (RIKEN BioResource Center; Wako, Japan) were maintained in 10% FCS low‐glucose DMEM at 37°C under 5% CO_2_ atmosphere. Prior to transfection, cells were diluted to 2.0 × 10^5^/ml in 1% FCS DMEM and cultured in 96‐well plates overnight. Cells in each well were transfected with reporter vectors (40 ng pGL3‐ ELAM, 20 ng pRL‐TK) and expression vectors (hTLR4/hMD‐2, 20 ng pCDNA3‐hMD‐2 and 40 ng pCDNA3‐hTLR4; mTLR4/mMD‐2, 20 ng pCDNA3‐mMD‐2 and 1 ng pCDNA3‐mTLR4; mTLR4/hMD‐2, 20 ng pCDNA3‐hMD‐2 and 1 ng pCDNA3‐mTLR4; and hMal, 0.1 or 0.5 ng pCDNA3‐hMal), complexed with 0.5 μl Lipofectamine LTX and 0.25 μl Plus reagent in 20 μl Opti‐MEM (Invitrogen), and subjected to stimuli 24 h after transfection.

### Cell stimulation, ELISA, and luciferase assay

Cells were primed for 30 min with various sphingolipids and then stimulated with TLR ligands. After 18‐h culture, media were collected and subjected to ELISA. ELISA kits for human IL‐6, human TNF‐α, human IL12/23 p40, and mouse TNF‐α were from BioLegend. Firefly and Renilla luciferase activities were measured using Dual‐Glo Luciferase Assay System (Promega; Australia) on a microplate reader (model Infinite M1000 PRO, Tecan Group; Männedorf, Switzerland).

### TLC and LC‐MS/MS analysis of GM3 species

Total lipids in lyophilized human serum were extracted with chloroform/ methanol (2:1 and 1:1, v/v) and separated into acidic and neutral fractions on DEAE‐Sephadex A‐25 anion‐exchange columns (GE Healthcare Life Sciences; Nitta *et al*, 2019). Acidic fraction was de‐esterified by mild alkaline hydrolysis for phospholipids, followed by desalting using a Sep‐Pak C18 cartridge (Waters; Milford, MA, USA). Acidic GSLs in mouse adipose tissues were separated by Ladisch's partitioning method as previously described (Tagami *et al*, 2002). Acidic GSLs (respective protein equivalent 100 μg [mouse adipose tissue] or respective volume equivalent 1 ml [human serum]) were spotted on HPTLC plates, developed, respectively, with chloroform/ methanol/ 0.2% CaCl_2_ (55:25:10, v/v/v) and chloroform/ methanol/ water (60:25:4, v/v/v), and visualized by orcinol/ sulfuric acid staining. Acidic GSLs were also subjected to LC‐MS/MS analysis by running method as described previously (Veillon *et al*, 2015; Go *et al*, 2017). 100 ng of the deuterated GM3 (d18:1‐[^13^C]16:0) was added for internal standard. Relative abundance of a particular GM3 species was expressed as peak area of that species divided by total peak area. For comparison of GM3 species among different mouse groups, total abundance of GM3 species in control group was defined as 1, and the abundances of each GM3 species in both control and fatty (e.g., HFD, *ob/ob*) group were normalized and displayed as relative amounts.

### Analysis of LC‐MS/MS data of GM3 species in sera of presymptomatic subjects and patients with metabolic disorders

LC‐MS/MS data and clinical markers of human subjects were obtained in previous study (Veillon *et al*, 2015), and relative abundances of ten major GM3 species (fatty acid: 16:0, 18:0, 20:0, 22:0, 23:0, 24:0, h24:0, 22:1, 24:1, h24:1) with reference to their total (defined as 1) were newly evaluated for each subject. Trends of each species in terms of pathological phases and Spearman's correlation coefficient in relation to clinical markers of metabolic disorders and chronic inflammation were analyzed. Self‐organization map (SOM) and Bayesian regularized neural‐network (BRNN) analysis were performed as described previously (Aoki *et al*, 2011).

### Animal studies

Six‐week‐old male *ob*/*ob* and control background (C57/BL6) mice were from CLEA Japan (Tokyo). Eight‐week‐old male C57/BL6, C3H/HeN, and C3H/HeJ (TLR4 mutant; Poltorak *et al*, 1998) mice (Japan SLC) were divided randomly into two groups. The control group was fed normal diet (ND) (CE‐2; CLEA Japan), while the HFD group was fed high‐fat diet (HFD) (D12492; Research Diet; New Brunswick, NJ, USA) ad lib for 8 weeks (C3H/HeN, C3H/HeJ) or 10 weeks (C57/BL6). Epididymal fat pads and blood (from right ventricle) were harvested from sacrificed animals, and non‐fasting blood glucose level was measured using Accu‐Chek Aviva strips (Roche DC; Japan).

### Cross‐linking and SDS–PAGE analysis

The recombinant mouse TLR4 (extracellular domain)/mMD‐2 proteins were prepared as described previously (Ohto *et al*, 2012a). Mouse TLR4/mMD‐2 complex proteins (1.5 μg) were mixed with GM3 18:0 (final 0.0071, 0.71, 71 μM) and incubated at room temperature for 1 h. LPS (0.05 μg) was added to the complex and incubated at 37°C for 1 h. Cross‐linking was performed by incubation with 0.6 μmol DMP (dimethyl pimelimidate; Thermo Fisher Scientific) at room temperature for 1 h. Cross‐linked protein complex was analyzed by SDS–PAGE (5–10% gradient gel) and silver‐stained.

### Ligand‐macromolecular docking

Molecular editing and lowest‐energy calculation of GM3 24:0 and 16:0 were performed on Avogadro molecular editing software (Hanwell *et al*, 2012), and their three‐dimensional structures were exported as PDB files. Ligand‐macromolecular docking between GM3 species and TLR4/MD‐2 complex was performed on AutoDock 4.2 molecular docking software (Morris *et al*, 2009). Grid settings for generating the binding surface on hTLR4/hMD‐2 were below: spacing, 0.431 Å; grid points, 60, 80, and 80; center of grids, 25, −13, and 15 (on x‐, y‐, and z‐axis). Grid settings for mTLR4/mMD‐2 were below: spacing, 0.431 Å; grid points, 80, 60, and 80; and center of grids, −30, −16, and 17 (on x‐, y‐, and z‐axis). Lamarckian genetic algorithm was used for searching candidate binding modes, and the binding mode with lowest energy was exploited from 100 calculation results. As a benchmark, rigid‐rigid dockings of Ra‐LPS (conformer in 3FXI) vs. hTLR4/hMD‐2 (3FXI, chains A and C) and lipid A (conformer in 3VQ2) vs. mTLR4/mMD‐2 (3VQ2, chains A and C) were performed (Appendix Fig S5D and E). Same settings and procedures for calculation were applied for searching rigid‐rigid binding modes of GM3 species on human and mouse TLR4/MD‐2 complex. All molecular/ protein structures were visualized by PyMOL software (DeLano Scientific).

### Ethics and informed consents for human‐subjected study

All participants gave their written informed consent prior to their inclusion in the study. The experimental protocol was in agreement with international norms and approved by the ethics committee of the University of Tokyo and Tohoku Medical and Pharmaceutical University.

### Statistical analysis

Data were expressed as mean ± SD and analyzed by two‐tailed unpaired *t*‐test or Tukey's multiple comparison (honesty significant difference) test using Microsoft Excel (Microsoft) and StatPlus:Mac Pro (AnalystSoft; Walnut, CA, USA). Differences between means were considered significant for *P* < 0.05 (*), < 0.01 (**), or < 0.001 (***).

## Author contributions

HK, TN, SG, KI, LV, WN, MF, KK, ASh, UO, TS, TW, HS, SA, KS, MN, YYa, NK, HA, HI, YN, YYo, AZ, AC, ML, MC, LM, ASu, and JI performed the research and analyzed the data. HK, KF, KS, MK, AP, SS, and JI. designed and supervised the research. HK and JI wrote the manuscript.

## Conflict of interest

The authors declare that they have no conflict of interest.

## Supporting information



Appendix

Expanded View Figures PDF

Review Process File

## Data Availability

The mass spec data of the GM3 species in human serum are available at the database GlycoPOST (https://glycopost.glycosmos.org). The accession number is GPST000057.
