# Supplementary material for: Homeostatic and pathogenic roles of GM3 ganglioside molecular species in TLR4 signaling in obesity
Source: EMBO J. 2020 May 7;39(12):e101732. doi: 10.15252/embj.2019101732 (PMC7298289; doi:10.15252/embj.2019101732)
Supplement: Supplementary file 1 — Appendix [file EMBJ-39-e101732-s001.pdf]

TABLE OF CONTENT

Appendix Figure 1
Values of clinical markers in presymptomatic subjects and patients with metabolic disorders.

Appendix Figure 2
Relative abundances of major serum GM3 species in various pathological phases.

Appendix Figure 3
Molecular characteristics of lipid-A/IVa species, eritoran, and GM3 species.

Appendix Figure 4
Co-stimulation of human TLR4/MD-2 variants by nickel ion plus proinflammatory GM3 22:0.

Appendix Figure 5
Binding model of VLCFA/LCFA-GM3 species on mTLR4/mMD-2 and results of benchmark calculation.

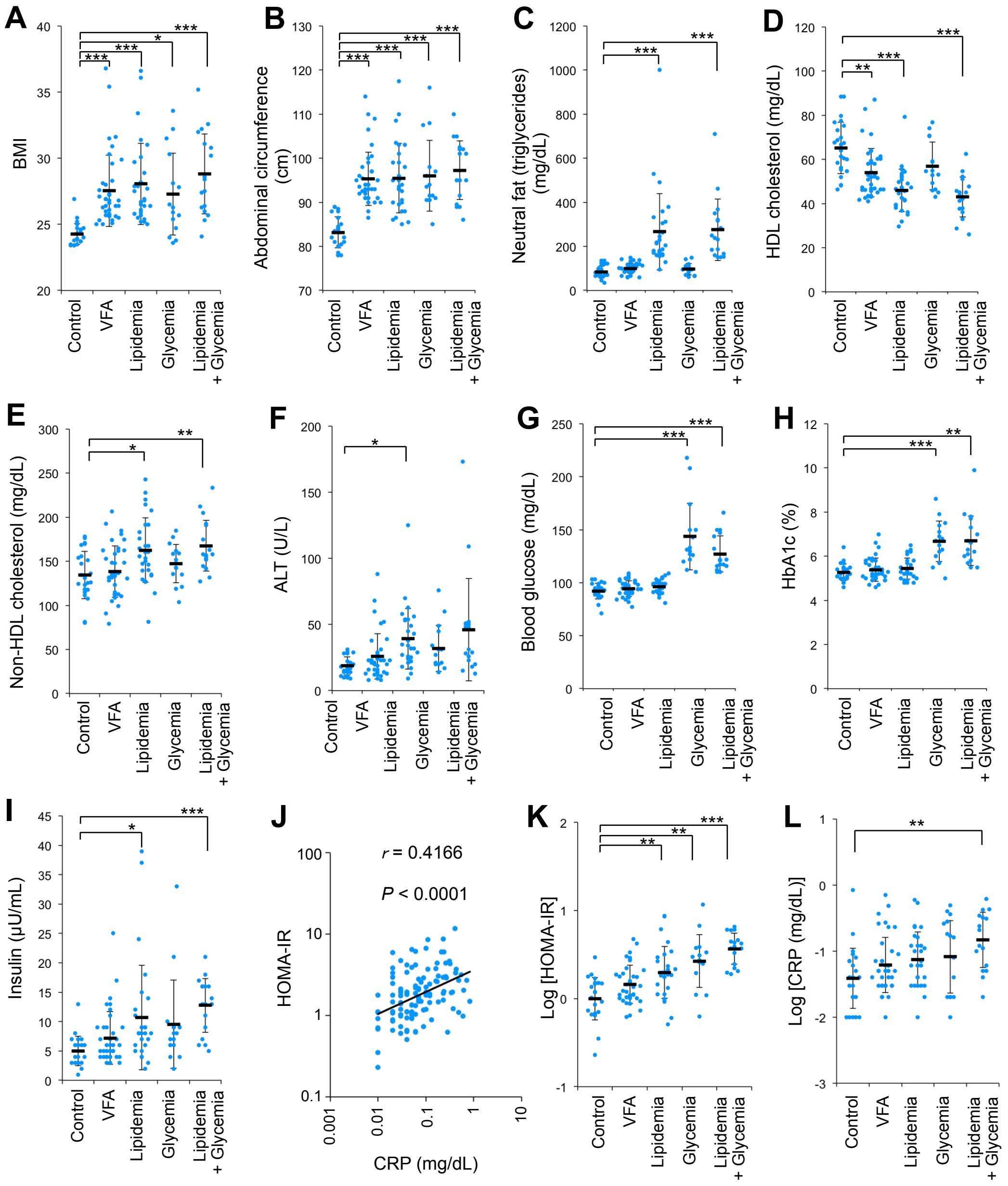

Appendix Figure 1 Values of clinical markers in presymptomatic subjects and patients with metabolic disorders. Values of clinical markers for metabolic disorders and for chronic inflammation in various pathological phases are shown. **(A)** BMI. **(B)** Abdominal circumference. **(C)** Serum natural fat (triglyceride). **(D)** Serum HDL cholesterol. **(E)** Serum non-HDL cholesterol. **(F)** Serum alanine transaminase (ALT). **(G)** Blood glucose. **(H)** HbA1c. **(I)** Serum insulin. **(J)** Spearman's correlation between HOMA-IR and serum CRP. **(K)** HOMA-IR (log scale). **(L)** Serum CRP (log scale). Data shown are individual values and mean ± SD (control, n=24; VFA, n=38; lipidemia, n=28; glycemia, n=15; lipidemia + glycemia, n=17), analyzed by two-tailed unpaired *t*-test with Bonferroni's correction. *p< 0.05, **p< 0.01, ***p< 0.001 for comparisons between indicated groups.

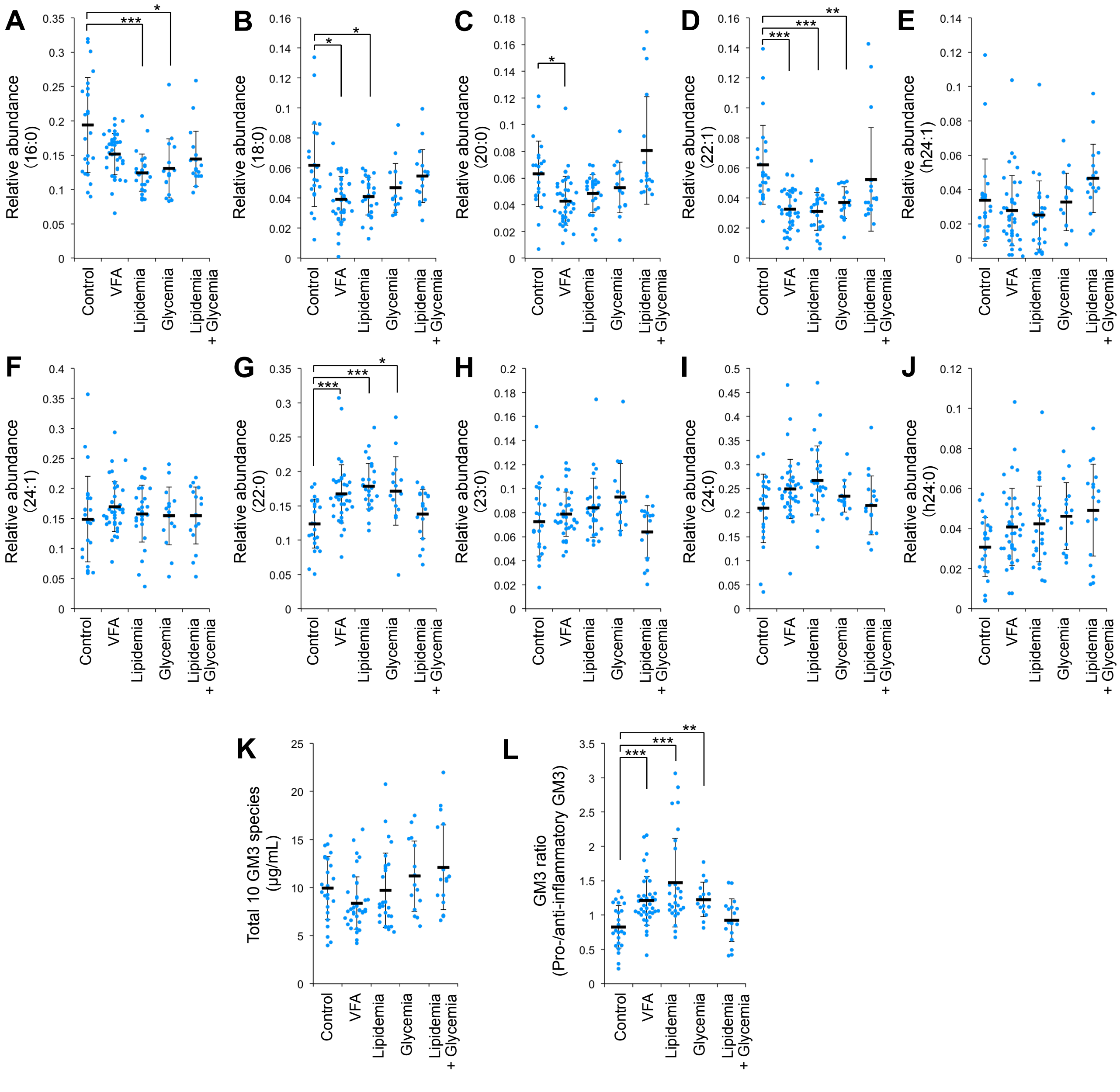

Appendix Figure 2 Relative abundances of major serum GM3 species in various pathological phases.

(A-J) Properties of various GM3 species, classified on the basis of acyl chain structures, as a function of pathological phase. Data shown are relative abundances of GM3 16:0 (A), 18:0 (B), 20:0 (C), 22:1 (D), h24:1 (E), 24:1 (F), 22:0 (G), 22:0 (H), 24:0 (I), and h24:0 (J) relative to total for ten major GM3 species (defined as 1.0) for each subject. **(K)** Total abundances of ten GM3 species (quantified as ratio to internal standard: ^{13}C-labeled GM3 [d18:1-]16:0) for each subject, at various pathological phases. **(L)** Ratios of proinflammatory GM3 (22:0, 24:0, 23:0, h24:0) to anti-inflammatory GM3 (16:0, 18:0, 20:0, 22:1, 24:1, h24:1) for each subject, at various pathological phases. Data shown are individual values and mean ± SD (control, n=24; VFA, n=38; lipidemia, n=28; glycemia, n=15; and lipidemia + glycemia, n=17), analyzed by two-tailed unpaired *t*-test with Bonferroni's correction. *p< 0.05, **p< 0.01, ***p< 0.001 for comparisons between indicated groups.

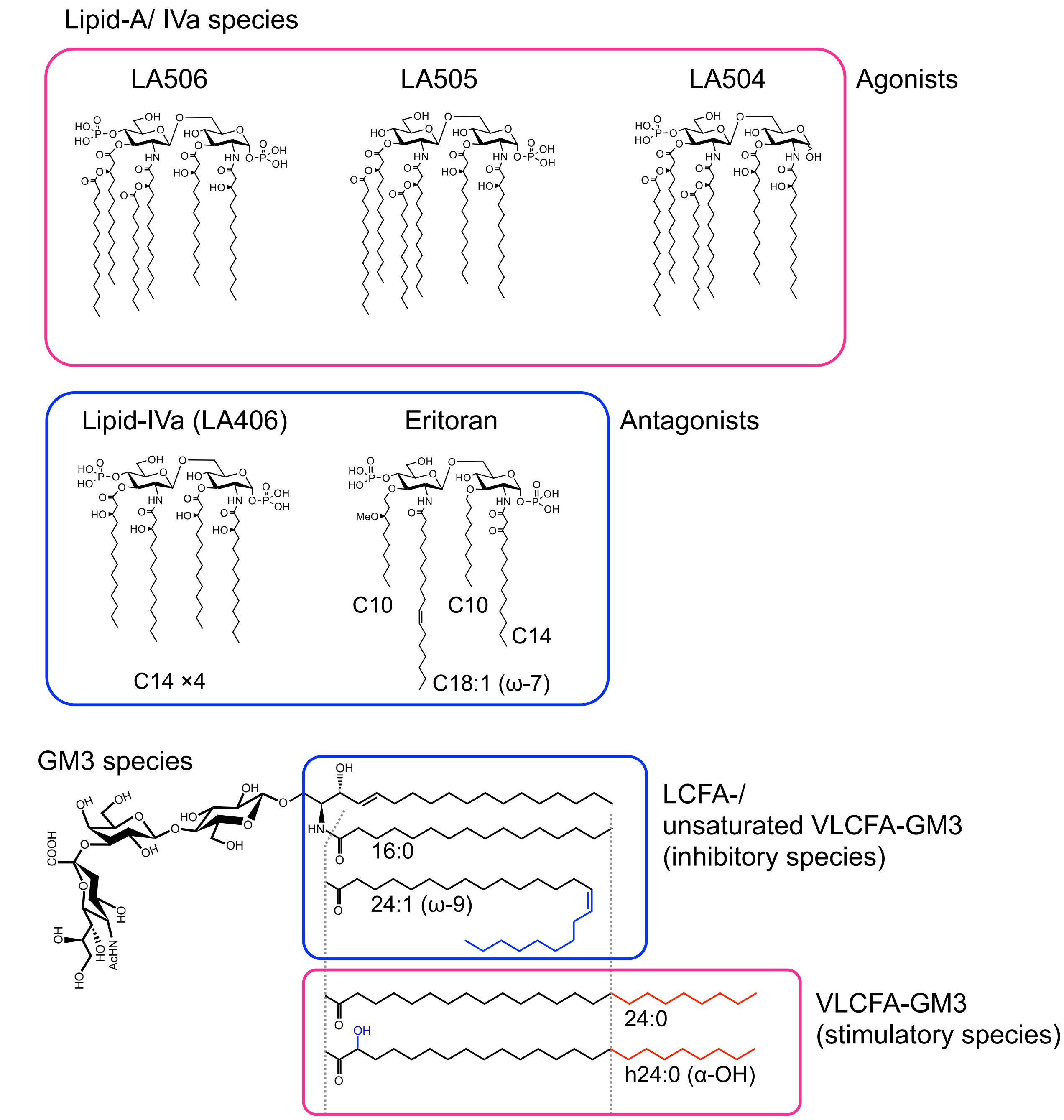

Appendix Figure 3 Molecular characteristics of lipid-A/IVa species, eritoran, and GM3 species. Molecular structures are shown for lipid-A 506 (LA506), lipid-A 505 (LA505), lipid-A 504 (LA504), lipid-IVa (LA406), and eritoran in comparison to GM3 species.

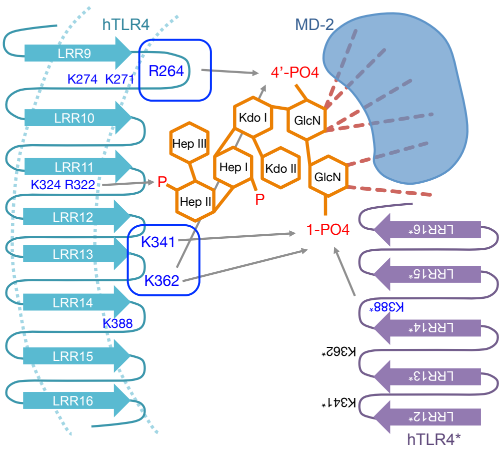

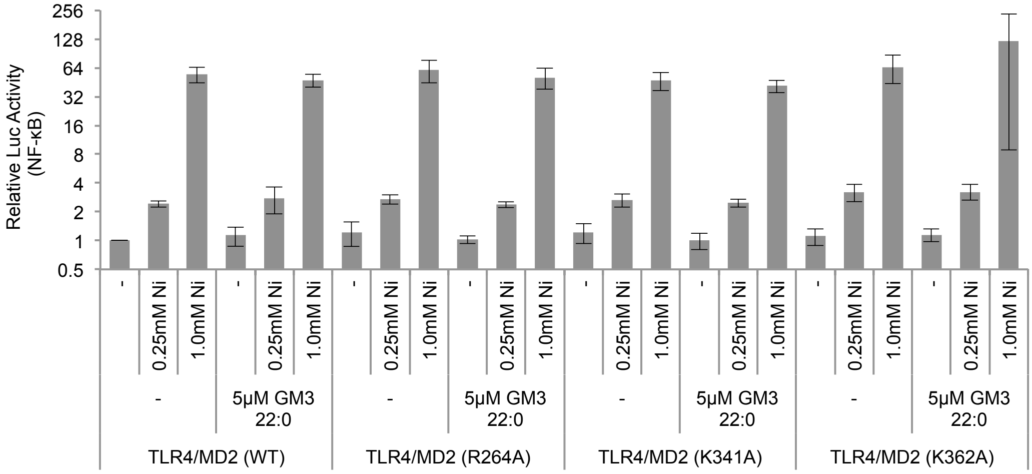

Appendix Figure 4 Co-stimulation of human TLR4/MD-2 variants by nickel ion plus proinflammatory GM3 22:0. Canonical LPS-binding residues on hTLR4/MD-2 complex and co-stimulation of HEK293T cells co-expressing hTLR4/hMD-2 by GM3 22:0 (5 µM) plus $NiSO_4$ (0.25, 1.0 mM). Data shown are mean ± SD (n=3).

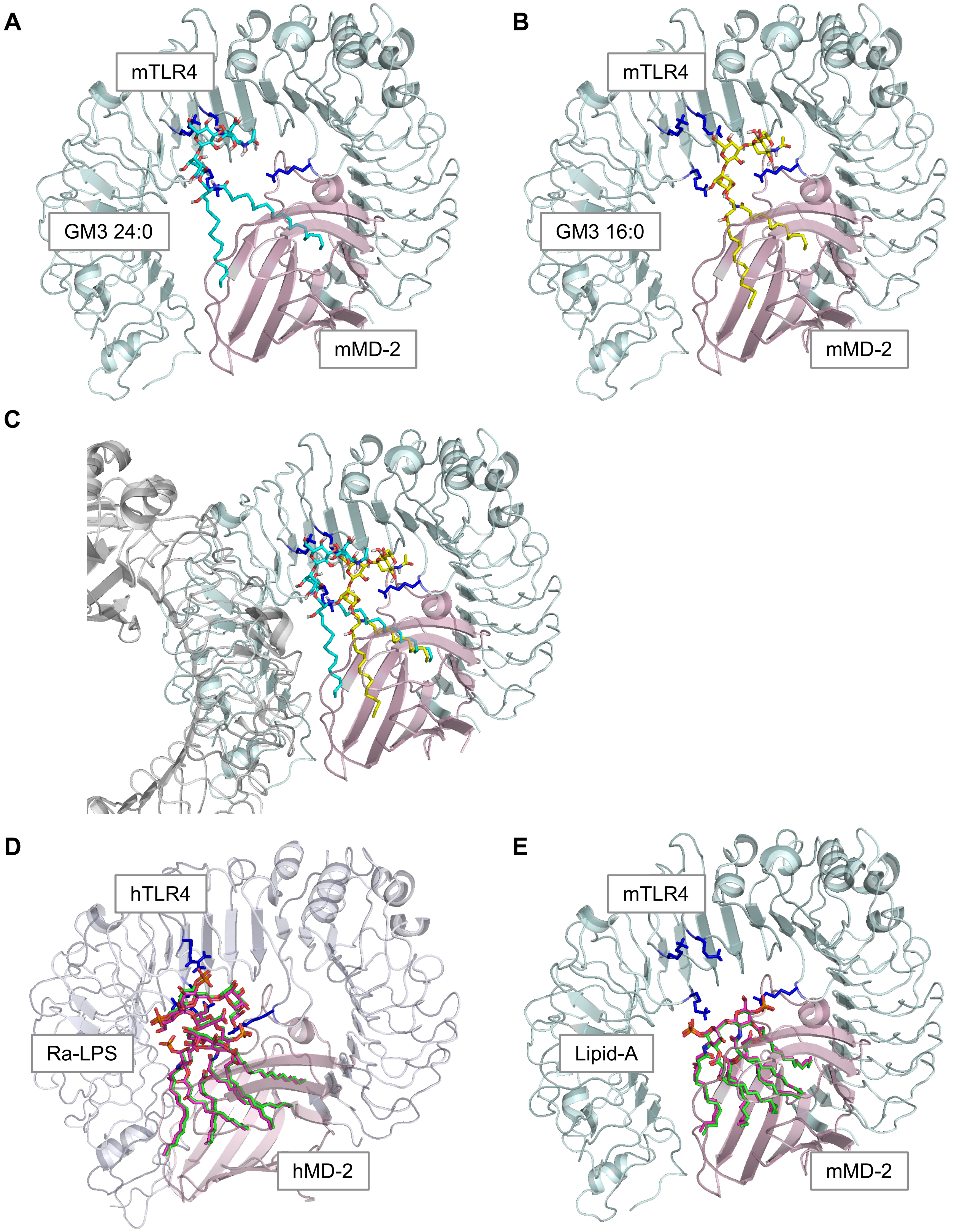

Appendix Figure 5 Binding model of VLCFA/LCFA-GM3 species on mTLR4/mMD-2 and results of benchmark calculation. (A, B) Docking model of GM3 24:0 **(A)** and 16:0 **(B)** binding to mouse TLR4/MD-2 complex (3VQ2). Basic residues of TLR4 are colored in blue. **(C)** Superposition of GM3 24:0 vs. GM3 16:0 on mouse TLR4/MD-2 complex. **(D, E)** Rigid-rigid docking calculation of Ra-LPS on hTLR4/hMD-2 complex **(D)** and lipid-A on mTLR4/mMD-2 **(E)** as a benchmark of this study. Superposition of docking results (green) vs. coordinates in reference structures (magenta) are shown.
