# Supplementary material for: Homeostatic and pathogenic roles of GM3 ganglioside molecular species in TLR4 signaling in obesity
Source: EMBO J. 2020 May 7;39(12):e101732. doi: 10.15252/embj.2019101732 (PMC7298289; doi:10.15252/embj.2019101732)
Supplement: Supplementary file 2 — Expanded View Figures PDF [file EMBJ-39-e101732-s002.pdf]

Expanded View Figures

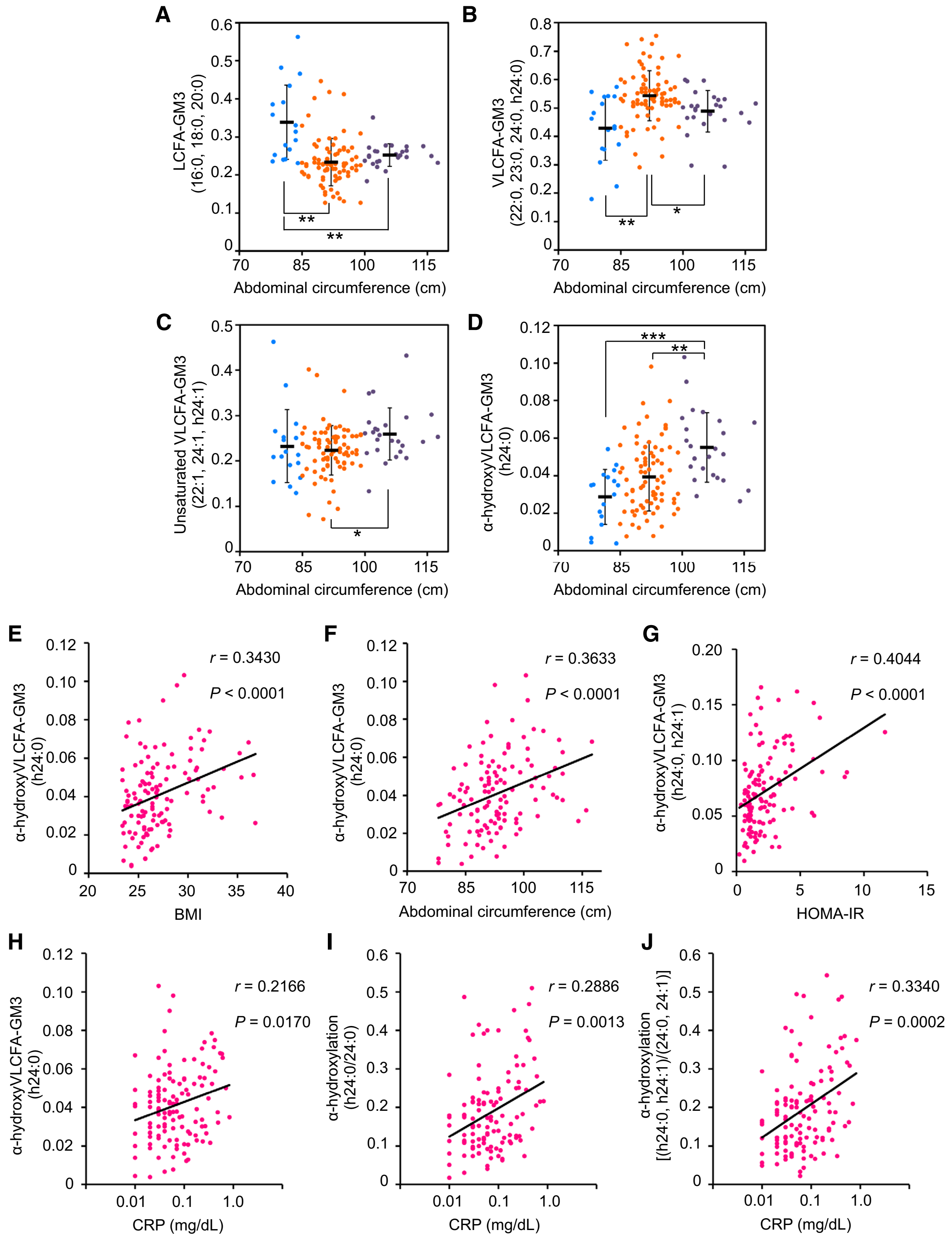

Figure EV1.

Figure EV1. Properties of various GM3 species as a function of clinical markers of metabolic disorders and chronic inflammation.

A–D LCFA species (A), VLCFA species (B), unsaturated VLCFA species (C), and α-hydroxy VLCFA-GM3 (h24:0) (D). Colors indicate disease severity: light blue, no abnormal scores (n = 17); orange, early-phase obesity (n = 80); purple, severe obesity (n = 25). Data shown are mean ± SD, analyzed by two-tailed unpaired t-test with Bonferroni's correction. *$P < 0.05$, **$P < 0.01$, and ***$P < 0.001$ for comparisons between indicated groups.

E–J Spearman's correlations for GM3 h24:0 vs. BMI (E), GM3 h24:0 vs. abdominal circumference (F), total of α-hydroxy GM3 (h24:0 and h24:1) vs. HOMA-IR (G), GM3 h24:0 vs. serum CRP (H), α-hydroxylation rate (h24:0 to 24:0) vs. serum CRP (I), and α-hydroxylation rate (h24:0 and h24:1 to 24:0 and 24:1) vs. serum CRP (J).

Data information: Sample sizes: (A–G), n = 122; (H-J), n = 121.

 © 2020 The Authors

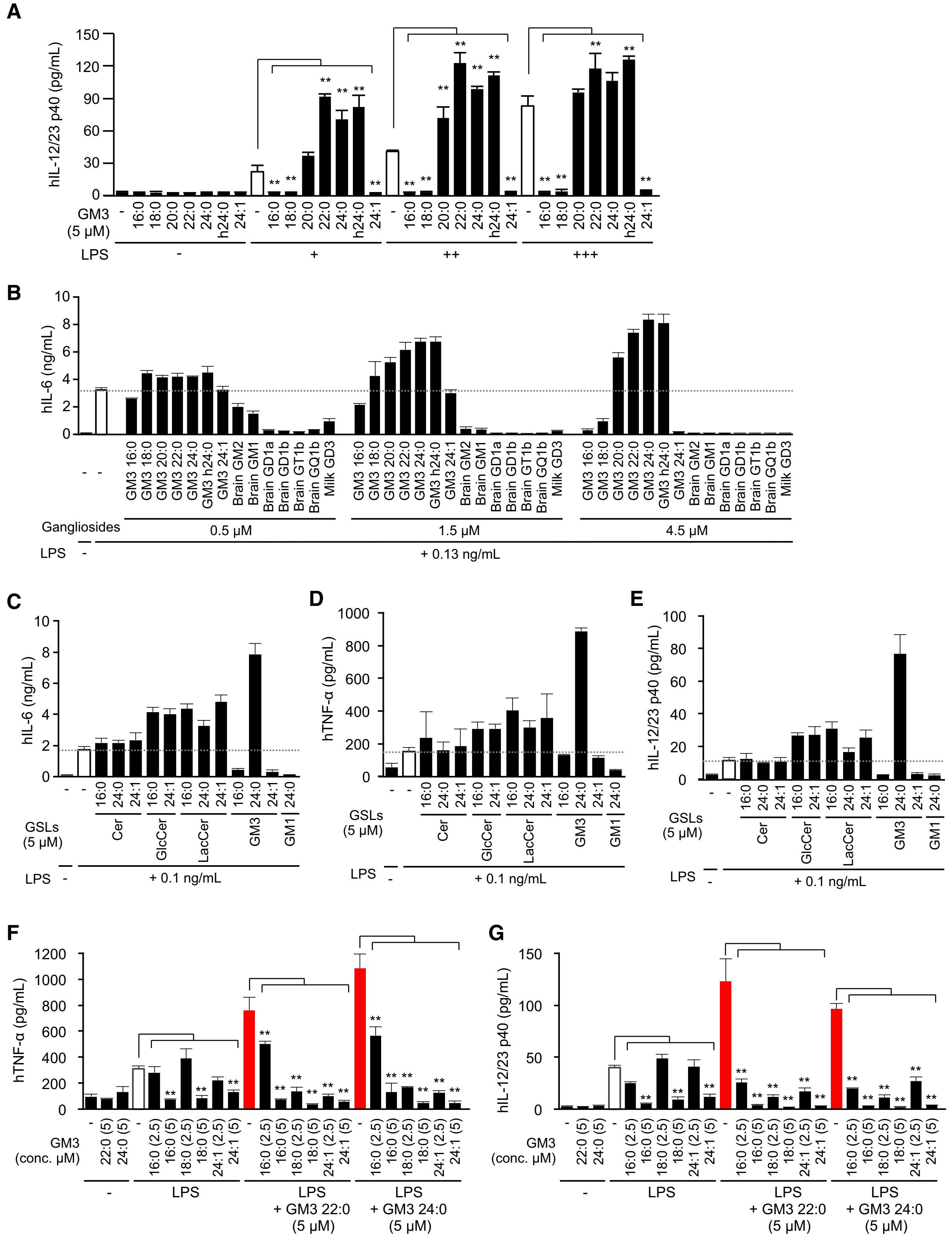
A
hIL-12/23 p40 (pg/mL)
GM3 (5 µM)
LPS
B
hIL-6 (ng/mL)
Gangliosides
0.5 µM
1.5 µM
4.5 µM
+ 0.13 ng/mL
C
hIL-6 (ng/mL)
D
hTNF-α (pg/mL)
E
hIL-12/23 p40 (pg/mL)
GSLs (5 µM)
Cer
GlcCer
LacCer
GM3
GM1
+ 0.1 ng/mL
F
hTNF-α (pg/mL)
G
hIL-12/23 p40 (pg/mL)
GM3 (conc. µM)
LPS + GM3 22:0 (5 µM)
LPS + GM3 24:0 (5 µM)

Figure EV2.

Figure EV2. Positive and negative regulation of innate immune response by GM3 gangliosides.

A GM3-mediated enhancement and inhibition of IL-12/23 production from LPS-stimulated monocytes (measured by ELISA).

B Co-stimulation of monocytes by LPS plus GM3 species or complex ganglioside species. IL-6 production in culture supernatant was measured by ELISA.

C–E Co-stimulation of monocytes by LPS plus GM3 species or precursor GSL species. The production of IL-6 (C), TNF-α (D), and IL-12/23 p40 (E) in culture supernatant was measured by ELISA.

F, G Inhibitory effect of LCFA and unsaturated VLCFA-GM3 on VLCFA-GM3 species. The production of TNF-α (F) and IL-12/23 p40 (G) in culture supernatant was measured by ELISA.

Data information: Data shown are mean ± SD ($n = 3$) analyzed by Tukey's multiple comparison test. $^{**}P < 0.01$ for comparison with LPS stimulation without GM3 species (A), or co-stimulation by LPS and proinflammatory GM3 (22:0, 24:0) (F, G).

 © 2020 The Authors

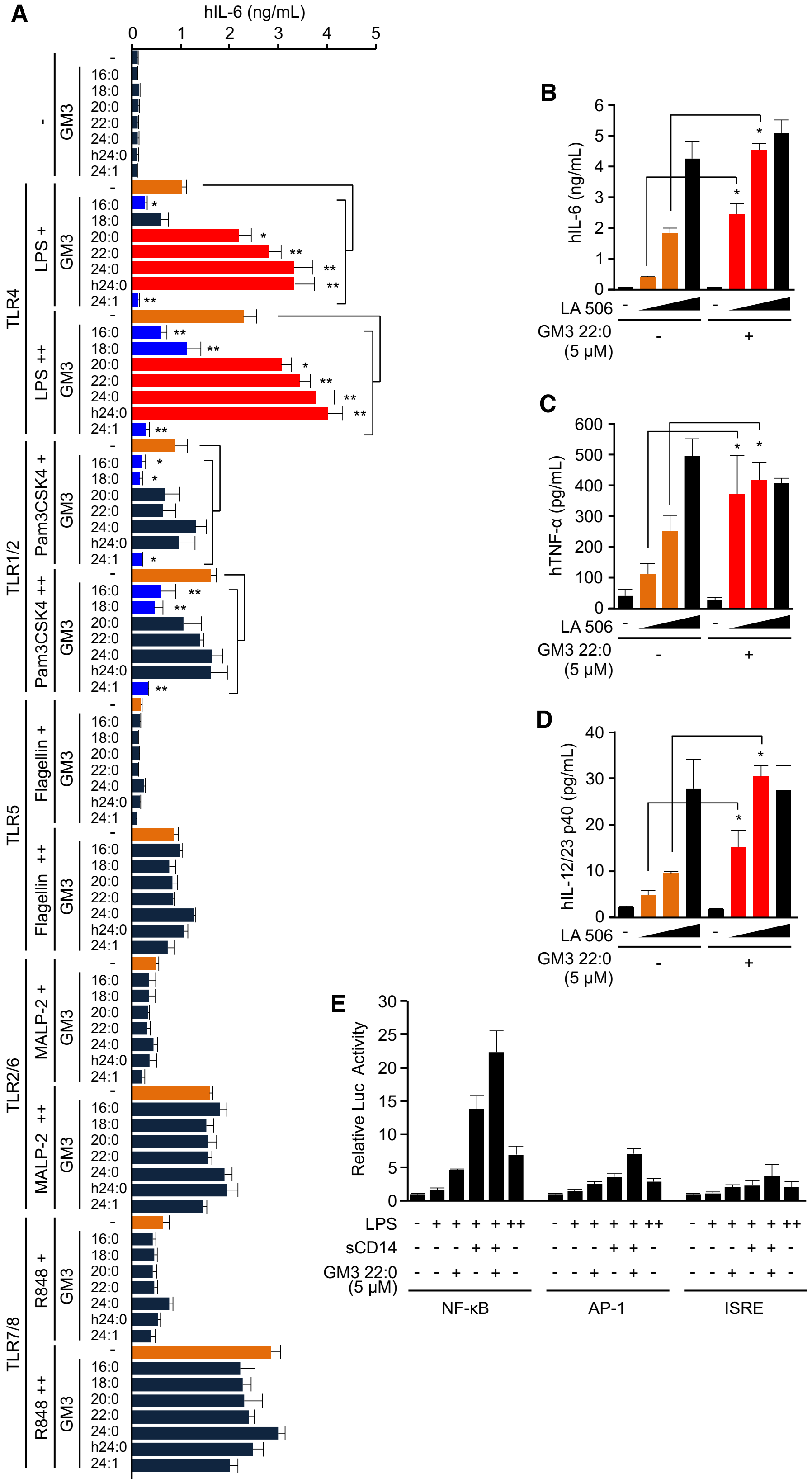

Figure EV3. VLCFA-GM3 species synergistically and selectively control human TLR4/MD-2 activation.

A Co-stimulation of human monocytes by GM3 species plus various TLR ligands: LPS (0.13, 0.25 ng/ml), TLR4/MD-2, Pam3CSK4 (0.25, 0.5 μg/ml), TLR1/2, Flagellin (10, 50 ng/ml), TLR5, R848 (0.25, 0.5 μg/ml), TLR7/8, MALP-2 (0.5, 1.0 ng/ml), and TLR2/6. IL-6 production in culture supernatant was quantified by ELISA.

B–D Production of IL-6 (B), TNF-α (C), and IL-12/23 p40 (D) in culture supernatant following co-stimulation of monocytes by GM3 species plus LA506 (synthetic TLR4 ligand) (3, 10, 30 ng/ml).

E Relative luciferase reporter activities of NF-κB, AP-1, and ISRE in response to LPS (+, 5 ng/ml; ++, 1 μg/ml), sCD14 (1 μg/ml), GM3 22:0 (5 μM), and their combinations. Relative luciferase activity of control condition was defined as 1 for every reporter gene.

Data information: Data shown are mean ± SD (n = 3, A–D; n = 4, E) analyzed by Tukey's multiple comparison test (A) or by two-tailed unpaired t-test (B–D). *$P < 0.05$ and **$P < 0.01$ for comparisons between indicated groups.

© 2020 The Authors

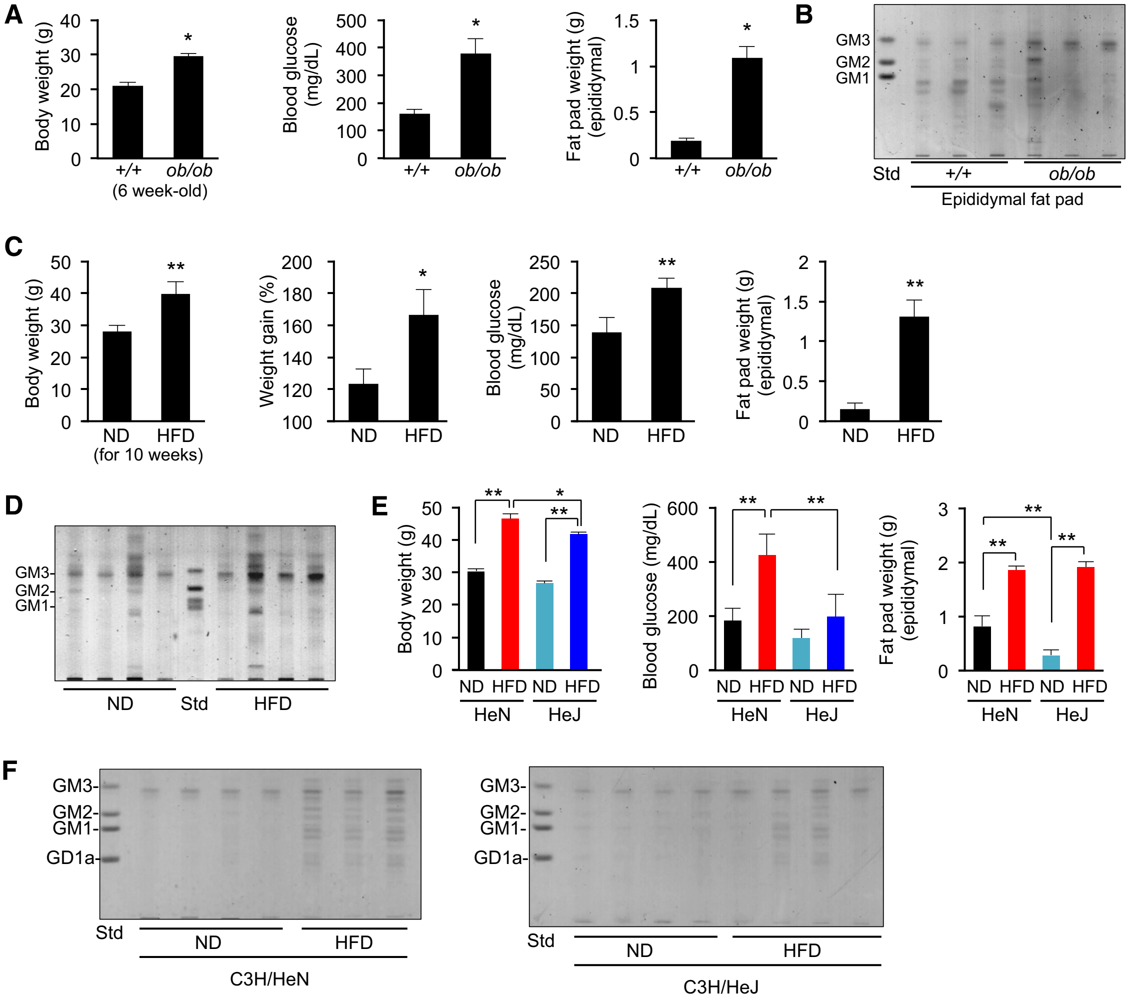

Figure EV4. GM3 ganglioside in adipose tissue showed increased abundance in early-phase obesity and short-term HFD.

A Body weight, blood glucose, and epididymal fat pad weight of 6-week-old control C57/BL6 mice and *ob/ob* mice (n = 3).

B Ganglioside species in epididymal fat pad were analyzed by TLC.

C Body weight, weight gain, blood glucose, and epididymal fat pad weight of normal diet (ND) and high-fat diet (HFD) C57/BL6 mice (n = 4).

D Ganglioside species in epididymal fat pad were analyzed by TLC.

E Body weight, blood glucose, and epididymal fat pad weight of C3H/HeN mice (ND, HFD) and C3H/HeJ mice (ND, HFD) (n = 4).

F Full-size images of Fig 7C. TLC analysis of acidic GSL fraction (equivalent to 0.1 mg protein) from epididymal fat pads of C3H/HeN and C3H/HeJ mice on ND or HFD for 8 weeks.

Data information: Data shown are mean ± SD analyzed by two-tailed unpaired *t*-test (A, C) or by Tukey's multiple comparison test (E). *$P < 0.05$ and **$P < 0.01$ for comparisons between indicated groups.

© 2020 The Authors

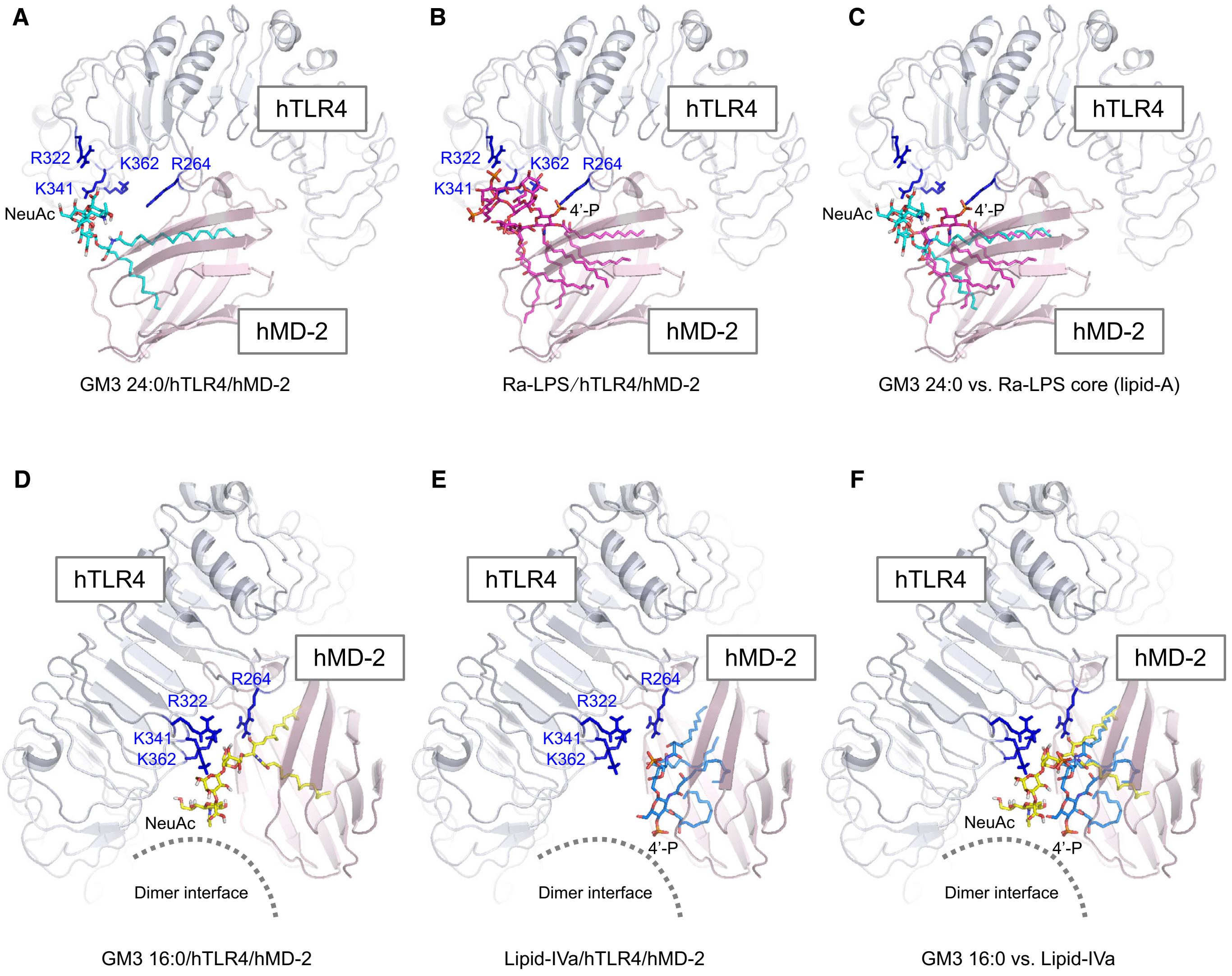

Figure EV5. Binding model of VLCFA/LCFA-GM3 species on hTLR4/hMD-2 and the comparison to LPS and lipid IVa.

A–C Docking model of GM3 24:0 (A), Ra-LPS (in 3FXI) (B), and superposition of GM3 24:0 vs. core structure of Ra-LPS (lipid A) (C).

D–F Docking model of GM3 16:0 (D), lipid IVa (in complex with hMD-2 in 2E59) (E), and superposition of GM3 16:0 vs. lipid IVa (F). Basic residues of hTLR4 are colored in blue.

© 2020 The Authors
