# Supplementary material for: Homeostatic and pathogenic roles of GM3 ganglioside molecular species in TLR4 signaling in obesity
Source: EMBO J. 2020 May 7;39(12):e101732. doi: 10.15252/embj.2019101732 (PMC7298289; doi:10.15252/embj.2019101732)
Supplement: Supplementary file 3 — Review Process File [file EMBJ-39-e101732-s003.pdf]

THE EMBO JOURNAL

Homeostatic and pathogenic roles of GM3 ganglioside molecular species in TLR4 signaling in obesity

Hirotaka Kanoh, Takahiro Nitta, Shinji Go, Kei-ichiro Inamori, Lucas Veillon, Wataru Nihei, Mayu Fujii, Kazuya Kabayama, Atsushi Shimoyama, Koichi Fukase, Umeharu Ohto, Toshiyuki Shimizu, Taku Watanabe, Hiroki Shindo, Sorama Aoki, Kenichi Sato, Mika Nagasaki, Yutaka Yatomi, Naoko Komura, Hiromune Ando, Hideharu Ishida, Makoto Kiso, Yoshihiro Natori, Yuichi Yoshimura, Asia Zonca, Anna Cattaneo, Marilena Letizia, Maria Ciampa, Laura Mauri, Alessandro Prinetti, Sandro Sonnino, Akemi Suzuki, and Jin-ichi Inokuchi

Review timeline:

Submission date:	7 February 2019
Editorial Decision:	25 February 2019
Resubmission:	23 May 2019
Editorial Decision:	23 July 2019
Revision received:	24 September 2019
Editorial Decision:	25 October 2019
Revision received:	22 January 2020
Editorial Decision:	24 February 2020
Revision received:	13 March 2020
Accepted:	23 March 2020

Editor: Karin Dumstrei

Transaction Report:

(Note: With the exception of the correction of typographical or spelling errors that could be a source of ambiguity, letters and reports are not edited. The original formatting of letters and referee reports may not be reflected in this compilation.)

1st Editorial Decision 25 February 2019

Thank you for submitting your manuscript to the EMBO Journal. I am sorry for the delay in getting back to you, but we are at the moment receiving a large number of new submissions that unfortunately has lead to some delays in handling time.

I have now had a chance to read your manuscript carefully and to discuss it with the other members of our editorial team. In addition, I have also sought external advice on the manuscript from a good expert in the field. I am sorry to inform you that we find that the manuscript is not well suited for publication in the EMBO Journal and that we therefore have decided not to proceed with its handling and peer review.

Your analysis reports on the role of GM3 ganglioside lipids in chronic inflammation. Your analysis provides a careful characterization of GM3 species in human sera and in samples from patients at different stages of chronic inflammation. The findings show that GM3 species have different properties in regulating anti and proinflammatory cytokine production. While very-long-chain GM3s promote the expression or pro-inflammatory ones, long-chain GM3 species promote expression of ant-inflammatory ones. Different GM3 species are also associated with different phases of chronic diseases. I really appreciate the dataset and see that this provides a valuable resource for the community. However, previous work has also reported a link between glycosphingolipids and TLR4 signaling and also that lipids can have different effects on inflammation. I see that the findings add new insight into the role of ganglioside lipids in

© European Molecular Biology Organization

inflammation, but we also gain limited further insight into how the different GM3 species differentially affect the expression of ant/pro inflammatory species.

As mentioned above I have also sought advice on the study from a good expert in the field. However, I am afraid that our advisor reached a conclusion very similar to ours and found that the study would be most suitable published in the more specialized literature. Given these considerations, we therefore find it unlikely that the manuscript would fare well under review here.

I apologise for the delayed response and I am sorry that I can't be more positive on this occasion.

Resubmission 23 May 2019

I appreciate very much for your critical and valuable comments to our paper (EMBOJ-2019-101732) entitled "Homeostatic and pathogenic roles of GM3 ganglioside molecular species in TLR4 signaling in obesity".

Your main concern was that how the different GM3 species differentially affect the expression of ant/pro inflammatory species. In order to gain insight into this issue, we performed molecular modelling and docking studies between GM3 species and human TLR4/MD2 as you can see Fig. 8 and corresponding expanded view figure 5 and appendix figure 5 in the attached new manuscript. I believe that we could understand at least in part of molecular mechanisms of agonistic effect of very long chain (VLCFA)-GM3 species and antagonistic effect of long chain (LCFA)-GM3 on LPS/HMGB-1 induced TLR 4 activation. You can see the distinct and contrasting effects of VLCFA-GM3 and LCFA-GM3 are similar to distinct functions between Lipid A (activation) and Lipid IVa (inactivation).

I am most grateful if you could read our revised manuscript entitled "GM3 containing very long-chain fatty acid acts as endogenous TLR4 modulator and exacerbates metabolic disorders" and suggest the possibility to review our paper for EMBO Journal.

2nd Editorial Decision 23 July 2019

Thank you for submitting your manuscript to The EMBO Journal. Your study has now been seen by two referees and their comments are provided below.

As you can see from the comments below, the referees find the analysis interesting and comprehensive. They raise a number of issues that I would like to ask you to address in a revised version. Let me know if we need to discuss any points further.

I should add that it is EMBO Journal policy to allow only a single round of revision and that it is therefore important to address the raised concerns at this stage.

When preparing your letter of response to the referees' comments, please bear in mind that this will form part of the Review Process File, and will therefore be available online to the community. For more details on our Transparent Editorial Process, please visit our website: https://www.embopress.org/page/journal/14602075/authorguide#transparentprocess

We generally allow three months as standard revision time. As a matter of policy, competing manuscripts published during this period will not negatively impact on our assessment of the conceptual advance presented by your study. However, we request that you contact the editor as soon as possible upon publication of any related work, to discuss how to proceed. Should you foresee a problem in meeting this three-month deadline, please let us know in advance and we may be able to grant an extension.

Thank you for the opportunity to consider your work for publication. I look forward to your revision.

REFEREE REPORTS

Referee #1:

The manuscript by Kanoh et al shows that fatty acid chain length in specific gangliosides (GM3s) impacts the inflammatory activation of macrophages through modulation of TLR4 signaling. They furthermore show that in humans certain GM3s are plasma markers of obesity and metabolic health. The study is novel, the manuscript well-prepared and data abundant.

Minor points:

1. The authors use LPS as an equivalent to innate immune response. This is incorrect and needs to be adjusted throughout the manuscript. For making that point the authors should actually investigate the effects of GM3s on a "real" immune response in e.g. mice.

2. Is the switch from VLCFA-GM3 to LCFA-GM3 in adipose tissue in obesity happening in adipocytes or linked to immune cell infiltration?

3. In figure 6, the display of GM3 species as "Relative expression" is meaningless - wild-type and ob/ob mice obviously have very different adipose tissue - to me, most valid would be "nmol/mg adipocyte fraction". This is an important issue that usually arises when comparing lean and obese mice.

4. Is the switch from VLCFA-GM3 to LCFA-GM3 in adipose tissue linked to ceramide production or de novo lipogenesis? How is GM3S regulated in DIO in adipocytes and macrophages?

5. The data set in the GM3S mice remains anecdotal and misleading. Why did the authors choose to only perform 2 weeks of HFD feeding instead of more established 16 weeks protocols? The data are clear in that the GM3S KO mice don't seem to put on weight on HFD - but is this really linked to the mechanism proposed here by the authors? Lower weight gain means that there is a strong impact on energy metabolism (Tschöp et al. Nat Methods 2012). Any metabolic outcomes are usually driven by adiposity and therefore the data are confounded by the differences in body weight. Much more data is needed for this GM3S model. Also, if I understand correctly by deleting GM3S, both the beneficial and deleterious effects of GM3s are lost? If the authors don't want to provide a full set of energy metabolism data at this point I suggest omitting the data (as they are not helpful anyway).

6. Figure 5 does not show data obtained in a mouse model, rather it shows data in cells isolated from a mouse model. In the end, strong in vivo data in the context of DIO is missing.

7. The Abstract can be enhanced; it currently lacks proper contextualization. The language could be enhanced for clarity. While the English is acceptable, working on the text for better reading is advisable.

Referee #2:

General Summary. The manuscript by Kanoh identifies that specific sphingolipids, VLCFA-GM3, are able to synergistically enhance LPS-induced TLR4 activation. While I think there are some areas of the manuscript that should be improved upon (see comments below), the manuscript in general presents very strong evidence that the above is indeed the case. Several cell systems, both human and mouse, are used, along with a variety of molecular approaches that provide strong evidence that supports the principal claims of the manuscript. The breadth of the presented work is impressive, combining in vitro molecular work, structural modelling, along with human clinical data. Nonetheless, there are some limitations of manuscript. Specifically, further mechanistic studies demonstrating enhanced TLR4 dimerisation and the subsequent consequences on downstream signalling pathway activation would strengthen the work. In addition, I feel it is important that my Point 5 in particular is addressed.

Point 1. In reference to Figure 1K it is stated that only GM3 species, but not Cer, GlcCer or LacCer display synergistic effects. While it is difficult to fully interpret the data as presented (in a heat map), it appears to me that GluCer and LacCer potentiate the effects of LPS. I would like to see this data presented in bar graph format so it is more interpretable as well as confirmation that these species do not display the same synergistic effects as GM3 species.

Point 2.In reference to Figure 2C and D, S100A9 has been implicated as a factor that is released from adipocytes and can activate TLR4, potentially contributing to metabolic inflammation. It would be interesting to see if the VLCFA-GM3 species also synergise with S100A9.

Point 3.The inclusion of human clinical data together with the molecular work is a strength of the paper. With respect to the authors, I would consider re-ordering the current figures. The manuscript reads a little disjointed with the presentation of the clinical data in the middle of the manuscript. A better narrative might be achievable by having the clinical data at the beginning or end of the manuscript.

Point 4. The authors state that an important aspect of the synergistic activation of TLR4 activation by GM3 is that it occurs at low concentrations of LPS, and a molecular rationale for this is discussed. Indeed, such an effect could be quite relevant in the context of metabolic inflammation where the levels of endogenous activators of TLR4, e.g. LPS/S100A9, are likely to be quite low relative to acute inflammatory conditions (e.g. sepsis). Potentiation of these activators by VLCFA-GM3 could therefore have a meaningful impact on TLR4 activation in conditions such as obesity-driven inflammation. However, the authors do not formally address the effective concentrations of LPS. In figure 1, concentrations of 0.13 and 0.06ng/ml of LPS are used. These are well below the typical concentrations typically used in studies of LPS (10ng/ml to 1ug/ml is typical in in vitro experiments). An experiment in which a fixed GM3 concentration used in combination with an extensive LPS dose response would be very insightful. Do you still see synergism at high doses of LPS? Or, is it possible, similar to LipidIVa, that at high doses of LPS even VLCFA-GM3 acts as an antagonist of TLR4 activation? These experiments would be quite straight-forward to conduct and hopefully would give some useful insight.

Point 5.The in vivo mouse studies presented in figures 6E and F do not implicate TLR4 directly, they only show that the loss of GM3s protects against obesity and impaired glucose tolerance. This may well be due to a TLR4-independent role of GM3s. I therefore feel that the implication of the title of the manuscript, i.e. that increased GM3 species exacerbate metabolic diseases by modulating TLR4, is not entirely proven based on the data presented. Indeed, mice are only being fed a high fat diet for 2 weeks. This is a very early time point in the evolution of metabolic dysfunction in rodents and, importantly, typically at this time little adipose tissue inflammation would be present. Therefore, the phenotype of the GM3 KO mice shown by the authors is not likely to be due to decreased TLR4 activation but some other effect of losing GM3s. If the authors were able to show reduced TLR4 activation in the high fat fed GM3 KO mice, this would greatly strengthen their case.

Point 6. With reference to Figure 7, the authors present evidence that key residues within TLR4, which mediate stability of the active heterotetrameric complex, are required for GM3's synergistic effects. While the cross-linking experiments provide evidence that GM3 is enhancing dimerization, this aspect of the work would be greatly enhanced by the analysis of real time dimerization of TLR4 following LPS stimulation in cells treated with or without GM3. This can be done by a flow cytometry based assay using a specific TLR4 antibody that discriminates between active and non-active TLR4 (used extensively by the Kagan lab, see for example Zanoni et al 2015). This would provide more compelling evidence that GM3 is augmenting TLR4 dimerisation (and also endocytosis, a key component of TLR4 signalling) than the cross-linking assay.

Point 7. Finally, no signalling data is presented in the manuscript. I feel it is important that such data be added to give a more complete picture of the mechanistic basis by which VLCFA-GM3 is augmenting LPS responses. Time course pathway analysis, by for example immunoblotting, would add another level of mechanistic insight to the manuscript. With this and the other above experiments I have mentioned, the manuscript would provide and very comprehensive basis by which GM3s augment TLR4 signalling.

1st Revision - authors' response 24 September 2019

Referee #1:

The manuscript by Kanoh et al shows that fatty acid chain length in specific gangliosides (GM3s) impacts the inflammatory activation of macrophages through modulation of TLR4 signaling. They furthermore show that in humans certain GM3s are plasma markers of obesity and metabolic health. The study is novel, the manuscript well-prepared and data abundant.

Minor points:

1. The authors use LPS as an equivalent to innate immune response. This is incorrect and needs to be adjusted throughout the manuscript. For making that point the authors should actually investigate the effects of GM3s on a "real" immune response in e.g. mice.
Answer: We corrected the points "innate immune response" to the words such as "LPS-mediated monocyte activation".

2. Is the switch from VLCFA-GM3 to LCFA-GM3 in adipose tissue in obesity happening in adipocytes or linked to immune cell infiltration?
Answer: In general, mouse immune cells express complex gangliosides such as GM1 and GD1a although adipocytes express GM3 as predominant species [Ohashi M. Lipids 14, 52-57, 1979]. So, the switch of GM3 species might happen in adipocytes, and the increase of complex gangliosides (found slightly in EV.4F) would be due to immune cell infiltration as shown previously [Tanabe A. et al. Biochem Biophys Res Commun. 379, 547-52. 2009].

3. In figure 6, the display of GM3 species as "Relative expression" is meaningless - wild-type and ob/ob mice obviously have very different adipose tissue - to me, most valid would be "nmol/mg adipocyte fraction". This is an important issue that usually arises when comparing lean and obese mice.
Answer: In general, the content of ganglioside in cell/ tissue can be normalized by the protein content in the same sample [ref No. 16, 17, 43]. Therefore, in this study, we normalized the GM3 content by the protein content in the same adipose tissue as describe in the method section. For comparison of LC/MS data between control and obese mice, the average of total GM3 abundance in control group was defined as 1, and the abundances of each GM3 species in both control and fatty (HFD, ob/ob) group were normalized and displayed as relative amounts. So, those data (in Fig.7) are comparable among different mouse groups, and also among different GM3 species. To improve the manuscript more understandable, we additionally described such explanations above in the method section.

4. Is the switch from VLCFA-GM3 to LCFA-GM3 in adipose tissue linked to ceramide production or de novo lipogenesis? How is GM3S regulated in DIO in adipocytes and macrophages?
Answer: CerSs and ELOVLs control the acyl-chain length in the ceramide structure. As described in the discussion part, we suppose that both mechanism (ceramide synthesis and fatty-acid elongation) are involved in the molecular switching of GM3 species.

Furthermore, total GM3 expression could be increased in adipocytes by TNF-alpha stimulation as we reported previously [ref No. 16: Tagami et al., J. Biol. Chem. 3085-3092, 2002]. NF-kappaB activation induces SREBP-1 activation, which induces gene expression of Elovls and desaturases [ref No. 56: Oishi et al. 2016 Cell metab]. So, we suppose that pro-inflammatory cytokine production in obesity is largely involved in the molecular switching in GM3 species. These interesting but highly complex mechanisms should be clarified in future studies.

5. The data set in the GM3S mice remains anecdotal and misleading. Why did the authors choose to only perform 2 weeks of HFD feeding instead of more established 16 weeks protocols? The data are clear in that the GM3S KO mice don't seem to put on weight on HFD - but is this really linked to the mechanism proposed here by the authors? Lower weight gain means that there is a strong impact on energy metabolism (Tschöp et al. Nat Methods 2012). Any metabolic outcomes are usually driven by adiposity and therefore the data are confounded by the differences in body weight. Much more data is needed for this GM3S model. Also, if I understand correctly by deleting GM3S, both the

© European Molecular Biology Organization

beneficial and deleterious effects of GM3s are lost? If the authors don't want to provide a full set of energy metabolism data at this point I suggest omitting the data (as they are not helpful anyway).

Answer:

To improve the manuscript more understandable, we omitted the GM3S-KO data according to discussions in the comment 5 above.

In the reference No.17 (Nagafuku et al., Glycobiology 25; 303-315, 2015), we have already reported that GM3S-KO attenuated the chronic-inflammatory phenotypes and metabolic abnormalities in HFD mice by 10-week feeding. In this study, we were also interested in the early phenotypes, because the VLCFA-shift in human serum GM3 occurred before the onset of chronic inflammation and metabolic disorders. So, we tried 2-week protocol, and confirmed at least that GM3S-KO could attenuate some of the early phenotypes as well as shown in long-term phenotypes.

However, as indicated in the comment 5 above, knockout of GM3S might have a considerable impact on energy metabolism inducing lower weight gain. We and other researchers have showed that TLR4 deficiency also induce lower weight gain, so, TLR4 signaling also confers a large impact on energy metabolism. It means that both GM3 expression and TLR4 signaling may regulate energy metabolism, and the details of their cooperative mechanisms should be investigated in future studies.

6. Figure 5 does not show data obtained in a mouse model, rather it shows data in cells isolated from a mouse model. In the end, strong in vivo data in the context of DIO is missing.

Answer:

We changed the subtitle: VLCFA-GM3 species selectively enhance mouse TLR4/MD-2 signaling. Additionally, to investigate the TLR4 signaling in GM3S-KO adipose tissue, there are some technical limitations. Discussions about in HFD experiments using knockout mouse have been described above in the answer for comment 5. Furthermore, to clarify the GM3-mediated inflammatory signaling in adipose tissue upon DIO, it would be better to newly establish the adipose-tissue cKO mouse to distinguish the local effect (by GM3 in visceral adipose tissue) and the systemic effect (by GM3 including other tissues). So, we would like to address these points more precisely in a future study.

7. The Abstract can be enhanced; it currently lacks proper contextualization. The language could be enhanced for clarity. While the English is acceptable, working on the text for better reading is advisable.

Answer: We refined the abstract as follows:

Innate immune signaling via TLR4 plays critical roles in pathogenesis of metabolic disorders. GM3 ganglioside in human serum is composed by a variety of fatty acids including long-chain (LCFA) and very long-chain (VLCFA). Serum VLCFA-GM3 increased significantly and LCFA-GM3 decreased sharply in metabolic disorders. Artificial intelligence based approaches revealed that GM3 species are significantly related to the disease symptoms. VLCFA-GM3 also increased in the adipose tissue of obese mice, and the increase of VLCFA-GM3 was attenuated in TLR4-mutant mice, implying an axis from TLR4 to GM3. In cultured monocytes, GM3 by itself had no effects on TLR4 activation; however, VLCFA-GM3 synergistically and selectively augmented TLR4 activation by LPS/HMGB1, and in contrast, LCFA- and unsaturated VLCFA-GM3 suppressed TLR4 activation. GM3 interacted with extracellular regions of TLR4/MD-2, and modulated dimerization/oligomerization. Ligand-molecular docking study suggested that VLCFA- and LCFA-GM3 act as agonist and antagonist against TLR4 activation, respectively, by differentially binding to hydrophobic pocket of MD-2. Our findings suggest that VLCFA-GM3 is a risk factor for TLR4-mediated disease progression.

Referee #2:

General Summary. The manuscript by Kanoh identifies that specific sphingolipids, VLCFA-GM3, are able to synergistically enhance LPS-induced TLR4 activation. While I think there are some areas of the manuscript that should be improved upon (see comments below), the manuscript in general presents very strong evidence that the above is indeed the case. Several cell systems, both human and mouse, are used, along with a variety of molecular approaches that provide strong evidence that supports the principal claims of the manuscript. The breadth of the presented work is impressive, combining in vitro molecular work, structural modelling, along with human clinical data. Nonetheless, there are some limitations of manuscript. Specifically, further mechanistic studies demonstrating enhanced TLR4 dimerisation and the subsequent consequences on downstream

signalling pathway activation would strengthen the work. In addition, I feel it is important that my Point 5 in particular is addressed.

Point 1. In reference to Figure 1K it is stated that only GM3 species, but not Cer, GlcCer or LacCer display synergistic effects. While it is difficult to fully interpret the data as presented (in a heat map), it appears to me that GluCer and LacCer potentiate the effects of LPS. I would like to see this data presented in bar graph format so it is more interpretable as well as confirmation that these species do not display the same synergistic effects as GM3 species.
Answer: We refined the manuscript and figures, and showed these data also in bar graphs (in Fig. EV2 C-E). We added the description that monocyte activation was moderately enhanced in the presence of precursor GSL species, and reached to the maximum in the presence of GM3 24:0.

Point 2.In reference to Figure 2C and D, S100A9 has been implicated as a factor that is released from adipocytes and can activate TLR4, potentially contributing to metabolic inflammation. It would be interesting to see if the VLCFA-GM3 species also synergise with S100A9.
Answer: We are also interested in the functions of S100 proteins in chronic inflammation. In addition to S100A9, S100A8 also potentiates TLR4 activation in sepsis (ref No. 38), and recently, Vogl T. et al. reported that homotypic-/ heterotypic interactions between S100A8 and A9 proteins and binding to calcium ion could modulate their function as TLR4 ligands (J Clin Invest. 128,1852–1866. 2018). These S100A proteins could induce further expression of serum amyloid-A (SAA) proteins, which is involved in chronic inflammation in cancer metastasis (ref No. 39). SAA proteins are acute serum proteins increased in sepsis and also in obesity, and recently known as endogenous TLR2/4 ligands. Since GM3 species showed selectivity to TLR4, and to TLR2 partially, we have already started to investigate the functional interactions among GM3 species and endogenous TLR2/4 ligands, S100A and SAA proteins. However, to figure out the detail of molecular mechanism and target receptor selectivity, we would like to address these points more deeply in a future study.

Point 3. The inclusion of human clinical data together with the molecular work is a strength of the paper. With respect to the authors, I would consider re-ordering the current figures. The manuscript reads a little disjointed with the presentation of the clinical data in the middle of the manuscript. A better narrative might be achievable by having the clinical data at the beginning or end of the manuscript.
Answer: We agree the suggestion to re-order the current figures, putting the clinical data at the beginning of manuscript as Fig.1 and 2. Accordingly, we refined abstract describing the clinical data first followed by in vitro experiments, mouse experiments, and then docking model study.

Point 4. The authors state that an important aspect of the synergistic activation of TLR4 activation by GM3 is that it occurs at low concentrations of LPS, and a molecular rationale for this is discussed. Indeed, such an effect could be quite relevant in the context of metabolic inflammation where the levels of endogenous activators of TLR4, e.g. LPS/S100A9, are likely to be quite low relative to acute inflammatory conditions (e.g. sepsis). Potentiation of these activators by VLCFA-GM3 could therefore have a meaningful impact on TLR4 activation in conditions such as obesity-driven inflammation. However, the authors do not formally address the effective concentrations of LPS. In figure 1, concentrations of 0.13 and 0.06ng/ml of LPS are used. These are well below the typical concentrations typically used in studies of LPS (10ng/ml to 1ug/ml is typical in in vitro experiments). An experiment in which a fixed GM3 concentration used in combination with an extensive LPS dose response would be very insightful. Do you still see synergism at high doses of LPS? Or, is it possible, similar to LipidIVa, that at high doses of LPS even VLCFA-GM3 acts as an antagonist of TLR4 activation? These experiments would be quite straight-forward to conduct and hopefully would give some useful insight.
Answer: We added the full data covering wider range of LPS concentrations (in Fig. 4B, C and Fig. EV2A). The synergy by VLCFA species is getting saturated in high concentration of LPS, which means that VLCFA species enhance low-grade activation of TLR4 without affecting maximal activation level. Fig. EV3 B-C also displayed the synergism in low Lipid-A concentration and the saturation in high Lipid-A concentration. On the other hand, the inhibition by LCFA/unsaturated species was observed still in high concentration of LPS (in Fig. 4B, C and Fig. EV2A).
As shown in several results, the optimal concentrations of LPS, that indicating dose-dependency, were different among cell types (human primary monocytes, < 0.5-1.0 ng/mL; mouse macrophages, < 1-2 ng/mL; transfected HEK293T cells, < 10-20 ng/mL).

Point 5. The in vivo mouse studies presented in figures 6E and F do not implicate TLR4 directly, they only show that the loss of GM3s protects against obesity and impaired glucose tolerance. This may well be due to a TLR4-independent role of GM3s. I therefore feel that the implication of the title of the manuscript, i.e. that increased GM3 species exacerbate metabolic diseases by modulating TLR4, is not entirely proven based on the data presented. Indeed, mice are only being fed a high fat diet for 2 weeks. This is a very early time point in the evolution of metabolic dysfunction in rodents and, importantly, typically at this time little adipose tissue inflammation would be present. Therefore, the phenotype of the GM3 KO mice shown by the authors is not likely to be due to decreased TLR4 activation but some other effect of losing GM3s. If the authors were able to show reduced TLR4 activation in the high fat fed GM3 KO mice, this would greatly strengthen their case.
Answer: In the reference No.17 (Nagafuku et al., Glycobiology 25; 303-315, 2015), we have already reported that GM3S-KO attenuated the chronic-inflammatory phenotypes and metabolic abnormalities in HFD mice by 10-week feeding. In this study, we were also interested in the early phenotypes, because the VLCFA-shift in human serum GM3 occurred before the onset of chronic inflammation and metabolic disorders. So, we tried 2-week protocol, and confirmed at least that GM3S-KO could attenuate some of the early phenotypes as well as shown in long-term phenotypes. However, as pointed out in the comment above, it would be difficult to detect very small inflammatory symptoms in such a pathological stage. So, taken together, to improve the manuscript more understandable, we omitted the data according to discussions in the point 5.
To investigate the TLR4 signaling in GM3S-KO adipose tissue, there are some technical limitations. High-fat-diet feeding induces chronic inflammation via TLR4 in visceral adipose tissue, but other signaling pathways are also activated simultaneously. On the other hand, LPS injection potentiates TLR4 signaling pathway selectively, but it induces strong (acute) systemic inflammation that could not be controlled as chronic adipose-tissue inflammation. Furthermore, to compare adipose-tissue inflammation via TLR4 (induced by LPS injection) between WT and GM3S-KO mouse, it would be better to newly establish the adipose-tissue cKO mouse to distinguish the local effect (by GM3 in visceral adipose tissue) and the systemic effect (by GM3 including other tissues). So, we would like to address these points more deeply in a future study.

Point 6. With reference to Figure 7, the authors present evidence that key residues within TLR4, which mediate stability of the active heterotetrameric complex, are required for GM3's synergistic effects. While the cross-linking experiments provide evidence that GM3 is enhancing dimerization, this aspect of the work would be greatly enhanced by the analysis of real time dimerization of TLR4 following LPS stimulation in cells treated with or without GM3. This can be done by a flow cytometry based assay using a specific TLR4 antibody that discriminates between active and non-active TLR4 (used extensively by the Kagan lab, see for example Zanoni et al 2015). This would provide more compelling evidence that GM3 is augmenting TLR4 dimerisation (and also endocytosis, a key component of TLR4 signalling) than the cross-linking assay.
Answer: In general, the FACS analyses using mAb MTS510 (specific to surface TLR4/MD-2 monomer) and mAb Sa15-21 (pan detection of surface TLR4) are effective to investigate LPS-mediated TLR4 internalization and to estimate the dimerization. On the other hand, these methods are established on the basis of LPS-mediated signaling events in mouse immune cells. High LPS concentration is required for starting activation of almost all cell-surface TLR4 simultaneously (in a short time), rather than inducing chronic activation of TLR4 persistently (for a long time). So, it is ambiguous whether these methods are applicable to analysis for chronic signaling events mediated by endogenous ligands or low concentration LPS. Therefore, in a future study, we would like to clarify whether the FACS analysis is effective for chronic TLR4 activation and also for the synergistic signaling event by VLCFA GM3 species.

Point 7. Finally, no signalling data is presented in the manuscript. I feel it is important that such data be added to give a more complete picture of the mechanistic basis by which VLCFA-GM3 is augmenting LPS responses. Time course pathway analysis, by for example immunoblotting, would add another level of mechanistic insight to the manuscript. With this and the other above experiments I have mentioned, the manuscript would provide and very comprehensive basis by which GM3s augment TLR4 signalling.
Answer: We tried to investigate the activation of several signaling pathways downstream of TLR4. We succeeded to detect and compare the activities of NF-κB response element, AP-1 response element (downstream of MAPK), and interferon-stimulated response element (ISRE) (downstream of IRF3/7 or feedback from type-I interferon production), and the results were shown in Fig. EV3 E.

© European Molecular Biology Organization

NF-κB response element activity was most strongly activated by LPS and GM3 22:0. Similarly, AP-1 response element activity was moderately activated, suggesting that GM3-mediated enhancement of TLR4 signaling is dependent on NF-κB and MAPK signaling pathway. On the other hand, ISRE activity was enhanced only weakly, implying that the contribution of the IRF3/7 pathway is relatively small. In general, relatively strong LPS stimulation is required for analyzing protein phosphorylation (e.g. phospho-I-kappaB, phospho-MAPK) by western blotting. So, we chose the reporter assay that is applicable to chronic stimulation.

3rd Editorial Decision 25 October 2019

Thank you for submitting the revised manuscript to the EMBO Journal. Your study has now been re-reviewed by the referees. The referees appreciate that the revisions have resolved some of the concerns raised. However, they also find that some of the concerns raised have not been addressed fully and that some additional work is needed to sort this out.

Should you be able to address the last remaining issues then I would like to invite you to submit a revised version. Let me know if we need to discuss any issues further

----

REFEREE REPORTS

Referee #1:

This is a revised version. My enthusiasm is still very high for this study; however, I am a little puzzled that the authors instead of addressing my questions experimentally they hand-wave, pointing to a lot of maybes.

My overall primary question was/is on the obesity-induced changes in the adipose tissue, which is a main theme of the study. In the title they indicate the VLCFA-GM3s relevance for "obesity". In the human part they show very interesting plasma data. In the mouse in vivo part show that adipose tissue content is altered in obesity. In the in vitro part they use monocytes/macrophages. In the end it remains unclear how much of this mechanism is linked to obesity per se or could one take any inflammatory condition in macrophages and find the same thing. In other words, the link to obesity and adipose tissue biology is weak. So, either the authors settle these issues experimentally or dedicate a large part of the discussion to this limitation, as pointed out below.

Alternatively/additionally, this paper would benefit from a comment putting this work into context.

Remaining (previous) points:

1. Is the switch from VLCFA-GM3 to LCFA-GM3 in adipose tissue in obesity happening in adipocytes or linked to immune cell infiltration? The authors responded that, in general, mouse immune cells express complex gangliosides such as GM1 and GD1a although adipocytes express GM3 as predominant species [Ohashi M. Lipids 14, 52-57, 1979]. So, the switch of GM3 species might happen in adipocytes, and the increase of complex gangliosides (found slightly in EV.4F) would be due to immune cell infiltration as shown previously [Tanabe A. et al. Biochem Biophys Res Commun. 379, 547-52. 2009]. Here the authors hand-wave and fail to answer my question. I have looked at the Tanabe paper and the in vivo measurements are weak. It is clear that 3T3-L1 adipocytes have more GM3s whereas RAW macrophages have more GM1s and GM2s. So, in the end LC-MS measurement of the adipocyte vs macrophage fraction would corroborate the previous observations and settle this issue. This is also relevant for the overall concept: the authors ASSUME that the adipocytes change their GM3 composition due to HFD, from LCFA to VLCFA, and this impacts the signaling in the macrophage but there are no data showing this.

2. In the new figure 7, the display of GM3 species as "Relative expression" is meaningless - wild-type and ob/ob mice obviously have very different adipose tissue. This is an important issue that usually arises when comparing lean and obese mice. The authors responded that, in general, the

content of ganglioside in cell/ tissue can be normalized by the protein content in the same sample. I respectfully disagree. Adipose tissue in a lean mouse (per fat pad, e.g. epididymal) is comprised of mainly fat cells and low protein content per fat pad. In the obese roughly 90% of the cells are macrophages and the protein content is completely changed per g tissue and also per fat pad. So, the reasoning of the authors is flawed. Again, what is changed in the adipocyte fraction vs. the macrophages fraction would be most relevant. Is the change in GM3 an obesity-induced switch in the adipocyte or is it just more macrophages in the tissue. This is unclear.

Referee #2:

Point 1. In reference to Figure 1K it is stated that only GM3 species, but not Cer, GlcCer or LacCer display synergistic effects. While it is difficult to fully interpret the data as presented (in a heat map), it appears to me that GluCer and LacCer potentiate the effects of LPS. I would like to see this data presented in bar graph format so it is more interpretable as well as confirmation that these species do not display the same synergistic effects as GM3 species.

Answer: We refined the manuscript and figures, and showed these data also in bar graphs (in Fig. EV2 C-E). We added the description that monocyte activation was moderately enhanced in the presence of precursor GSL species, and reached to the maximum in the presence of GM3 24:0.

Reviewer Response: Satisfied with the response.

Point 2.In reference to Figure 2C and D, S100A9 has been implicated as a factor that is released from adipocytes and can activate TLR4, potentially contributing to metabolic inflammation. It would be interesting to see if the VLCFA-GM3 species also synergise with S100A9.

Answer: We are also interested in the functions of S100 proteins in chronic inflammation. In addition to S100A9, S100A8 also potentiates TLR4 activation in sepsis (ref No. 38), and recently, Vogl T. et al. reported that homotypic-/ heterotypic interactions between S100A8 and A9 proteins and binding to calcium ion could modulate their function as TLR4 ligands (J Clin Invest. 128,1852-1866. 2018). These S100A proteins could induce further expression of serum amyloid-A (SAA) proteins, which is involved in chronic inflammation in cancer metastasis (ref No. 39). SAA proteins are acute serum proteins increased in sepsis and also in obesity, and recently known as endogenous TLR2/4 ligands. Since GM3 species showed selectivity to TLR4, and to TLR2 partially, we have already started to investigate the functional interactions among GM3 species and endogenous TLR2/4 ligands, S100A and SAA proteins. However, to figure out the detail of molecular mechanism and target receptor selectivity, we would like to address these points more deeply in a future study.

Reviewer Response: The inclusion of some S100 experiments would have been nice, but it's not essential to the manuscript. So satisfied with the response.

Point 3. The inclusion of human clinical data together with the molecular work is a strength of the paper. With respect to the authors, I would consider re-ordering the current figures. The manuscript reads a little disjointed with the presentation of the clinical data in the middle of the manuscript. A better narrative might be achievable by having the clinical data at the beginning or end of the manuscript.

Answer: We agree the suggestion to re-order the current figures, putting the clinical data at the beginning of manuscript as Fig.1 and 2. Accordingly, we refined abstract describing the clinical data first followed by in vitro experiments, mouse experiments, and then docking model study.

Reviewer Response: Satisfied with the response.

Point 4. The authors state that an important aspect of the synergistic activation of TLR4 activation by GM3 is that it occurs at low concentrations of LPS, and a molecular rationale for this is discussed. Indeed, such an effect could be quite relevant in the context of metabolic inflammation where the levels of endogenous activators of TLR4, e.g. LPS/S100A9, are likely to be quite low relative to acute inflammatory conditions (e.g. sepsis). Potentiation of these activators by VLCFA-

GM3 could therefore have a meaningful impact on TLR4 activation in conditions such as obesity-driven inflammation. However, the authors do not formally address the effective concentrations of LPS. In figure 1, concentrations of 0.13 and 0.06ng/ml of LPS are used. These are well below the typical concentrations typically used in studies of LPS (10ng/ml to 1ug/ml is typical in in vitro experiments). An experiment in which a fixed GM3 concentration used in combination with an extensive LPS dose response would be very insightful. Do you still see synergism at high doses of LPS? Or, is it possible, similar to LipidIVa, that at high doses of LPS even VLCFA-GM3 acts as an antagonist of TLR4 activation? These experiments would be quite straight-forward to conduct and hopefully would give some useful insight.

Answer: We added the full data covering wider range of LPS concentrations (in Fig. 4B, C and Fig. EV2A). The synergy by VLCFA species is getting saturated in high concentration of LPS, which means that VLCFA species enhance low-grade activation of TLR4 without affecting maximal activation level. Fig. EV3 B-C also displayed the synergism in low Lipid-A concentration and the saturation in high Lipid-A concentration. On the other hand, the inhibition by LCFA/unsaturated species was observed still in high concentration of LPS (in Fig. 4B, C and Fig. EV2A).
As shown in several results, the optimal concentrations of LPS, that indicating dose-dependency, were different among cell types (human primary monocytes, < 0.5-1.0 ng/mL; mouse macrophages, < 1-2 ng/mL; transfected HEK293T cells, < 10-20 ng/mL).

Reviewer Response: Thank you for adding the new data. It's unfortunate that higher doses of LPS were not used (0.25ng/ml, the highest dose used, is still low compared with what it typically used), as this may have shown a true saturation of the effect, which you do not have. Nonetheless, the combinatorial effects of LPS + GM3 are clearly lowered at higher doses of LPS, as would be predicted. Satisfied with the response.

Point 5. The in vivo mouse studies presented in figures 6E and F do not implicate TLR4 directly, they only show that the loss of GM3s protects against obesity and impaired glucose tolerance. This may well be due to a TLR4-independent role of GM3s. I therefore feel that the implication of the title of the manuscript, i.e. that increased GM3 species exacerbate metabolic diseases by modulating TLR4, is not entirely proven based on the data presented. Indeed, mice are only being fed a high fat diet for 2 weeks. This is a very early time point in the evolution of metabolic dysfunction in rodents and, importantly, typically at this time little adipose tissue inflammation would be present. Therefore, the phenotype of the GM3 KO mice shown by the authors is not likely to be due to decreased TLR4 activation but some other effect of losing GM3s. If the authors were able to show reduced TLR4 activation in the high fat fed GM3 KO mice, this would greatly strengthen their case.

Answer: In the reference No.17 (Nagafuku et al., Glycobiology 25; 303-315, 2015), we have already reported that GM3S-KO attenuated the chronic-inflammatory phenotypes and metabolic abnormalities in HFD mice by 10-week feeding. In this study, we were also interested in the early phenotypes, because the VLCFA-shift in human serum GM3 occurred before the onset of chronic inflammation and metabolic disorders. So, we tried 2-week protocol, and confirmed at least that GM3S-KO could attenuate some of the early phenotypes as well as shown in long-term phenotypes. However, as pointed out in the comment above, it would be difficult to detect very small inflammatory symptoms in such a pathological stage. So, taken together, to improve the manuscript more understandable, we omitted the data according to discussions in the point 5. To investigate the TLR4 signaling in GM3S-KO adipose tissue, there are some technical limitations. High-fat-diet feeding induces chronic inflammation via TLR4 in visceral adipose tissue, but other signaling pathways are also activated simultaneously. On the other hand, LPS injection potentiates TLR4 signaling pathway selectively, but it induces strong (acute) systemic inflammation that could not be controlled as chronic adipose-tissue inflammation. Furthermore, to compare adipose-tissue inflammation via TLR4 (induced by LPS injection) between WT and GM3S-KO mouse, it would be better to newly establish the adipose-tissue cKO mouse to distinguish the local effect (by GM3 in visceral adipose tissue) and the systemic effect (by GM3 including other tissues). So, we would like to address these points more deeply in a future study.

Reviewer Response: I concur with reviewer 1 and the authors that removing this data is reasonable.

Point 6. With reference to Figure 7, the authors present evidence that key residues within TLR4, which mediate stability of the active heterotetrameric complex, are required for GM3's synergistic

effects. While the cross-linking experiments provide evidence that GM3 is enhancing dimerization, this aspect of the work would be greatly enhanced by the analysis of real time dimerization of TLR4 following LPS stimulation in cells treated with or without GM3. This can be done by a flow cytometry based assay using a specific TLR4 antibody that discriminates between active and non-active TLR4 (used extensively by the Kagan lab, see for example Zanoni et al 2015). This would provide more compelling evidence that GM3 is augmenting TLR4 dimerisation (and also endocytosis, a key component of TLR4 signalling) than the cross-linking assay.

Answer: In general, the FACS analyses using mAb MTS510 (specific to surface TLR4/MD-2 monomer) and mAb Sa15-21 (pan detection of surface TLR4) are effective to investigate LPS-mediated TLR4 internalization and to estimate the dimerization. On the other hand, these methods are established on the basis of LPS-mediated signaling events in mouse immune cells. High LPS concentration is required for starting activation of almost all cell-surface TLR4 simultaneously (in a short time), rather than inducing chronic activation of TLR4 persistently (for a long time). So, it is ambiguous whether these methods are applicable to analysis for chronic signaling events mediated by endogenous ligands or low concentration LPS. Therefore, in a future study, we would like to clarify whether the FACS analysis is effective for chronic TLR4 activation and also for the synergistic signaling event by VLCFA GM3 species.

Reviewer Response: The authors are correct in stating that previous studies that have used this approach to assess TLR4 activation have typically used high concentrations of LPS. They also make a point about acute vs chronic stimulation that may be valid. However, these experiments are not challenging to conduct and I feel they should be performed. I would probably try co-treatments with LPS and GM3, as well as a more prolonged pre-treatment with GM3 (perhaps addressing the authors' comments about chronic stimulation), followed by LPS stimulation. I think these experiments are very worthwhile to conduct. If indeed they prove to be technically unfeasible due to the relatively low concentrations of the agonists being used (compared to the high concentrations of LPS typically used in these assays), then so be it. But I would comment that you are seeing a potentiation of TLR4-dependent increases in cytokine production, and so, according to the hypothesis being proposed by the authors, TLR4 dimerization/internalization should be detectable by these methods - which are very sensitive. The authors have not been asked for extensive experimental additions, so it's not unreasonable to expect that those requests for additional work, where they have been made, and which are technically quite straight forward, be attempted.

Point 7. Finally, no signaling data is presented in the manuscript. I feel it is important that such data be added to give a more complete picture of the mechanistic basis by which VLCFA-GM3 is augmenting LPS responses. Time course pathway analysis, by for example immunoblotting, would add another level of mechanistic insight to the manuscript. With this and the other above experiments I have mentioned, the manuscript would provide and very comprehensive basis by which GM3s augment TLR4 signaling.

Answer: We tried to investigate the activation of several signaling pathways downstream of TLR4. We succeeded to detect and compare the activities of NF-κB response element, AP-1 response element (downstream of MAPK), and interferon-stimulated response element (ISRE) (downstream of IRF3/7 or feedback from type-I interferon production), and the results were shown in Fig. EV3 E. NF-κB response element activity was most strongly activated by LPS and GM3 22:0. Similarly, AP-1 response element activity was moderately activated, suggesting that GM3-mediated enhancement of TLR4 signaling is dependent on NF-κB and MAPK signaling pathway. On the other hand, ISRE activity was enhanced only weakly, implying that the contribution of the IRF3/7 pathway is relatively small. In general, relatively strong LPS stimulation is required for analyzing protein phosphorylation (e.g. phospho-I-kappaB, phospho-MAPK) by western blotting. So, we chose the reporter assay that is applicable to chronic stimulation.

Reviewer Response: My feeling with regards to this point is somewhat to similar to that stated above. The reporter assays do not adequately address the point I raised. I would like to see whether signaling kinetics are altered. This type of analysis is very standard when assessing receptor activation. As I suggested above, perhaps the authors could try co-treatments with GM3 and LPS and look at acute (5 min to 4hrs) signaling pathways activation, as well as a chronic gm3 treatment followed by LPS acutely. Stating that relatively strong LPS stimulation is required for analyzing

protein phospho changes by immunoblotting is not accurate. The experiments should at least be attempted. If they are successful I think they add considerable insight.

2nd Revision - authors' response 22 January 2020

Referee #1:

This is a revised version. My enthusiasm is still very high for this study; however, I am a little puzzled that the authors instead of addressing my questions experimentally they hand-wave, pointing to a lot of maybes.
My overall primary question was/is on the obesity-induced changes in the adipose tissue, which is a main theme of the study. In the title they indicate the VLCFA-GM3s relevance for "obesity". In the human part they show very interesting plasma data. In the mouse in vivo part show that adipose tissue content is altered in obesity. In the in vitro part they use monocytes/macrophages. In the end it remains unclear how much of this mechanism is linked to obesity per se or could one take any inflammatory condition in macrophages and find the same thing. In other words, the link to obesity and adipose tissue biology is weak. So, either the authors settle these issues experimentally or dedicate a large part of the discussion to this limitation, as pointed out below. Alternatively/additionally, this paper would benefit from a comment putting this work into context.

Answer: Thank you for your valuable comments and suggestions. We have changed the title to " Homeostatic and pathogenic roles of GM3 ganglioside molecular species in TLR4 signaling in obesity" from "VLCFA-GM3 ganglioside acts as endogenous TLR4 modulator and exacerbates metabolic disorders". Our findings suggest that VLCFA-GM3 is a risk factor for TLR4-mediated disease progression. But also, we demonstrated that human TLR4/MD-2 received positive regulation by VLCFA-GM3 and negative regulation by LCFA-/ unsaturated VLCFA-GM3 in the presence of LPS and HMGB1. Increases of VLCFA-/ α-hydroxyl VLCFA-GM3 species, and decreases of LCFA-GM3 species, were involved in pathogenesis of metabolic disorders via chronic inflammatory processes. Moreover, LCFA-GM3 species such as 16:0 consistently inhibited TLR4 activation even in the presence of VLCFA-GM3 species 22:0 or 24:0; 18:0 and 20:0 (Fig. 5I). These findings indicate that GM3 species function as a rheostat for TLR4 signaling (Fig. 5J). Thus, the new title is more appropriate because GM3 species plays a role of rheostat for TLR-4 signaling.
So, we inserted a new sentence at the first paragraph of Discussion (page 15) as follows; LCFA-GM3 species such as 16:0 consistently inhibited TLR4 activation even in the presence of VLCFA-GM3 species 22:0 or 24:0; 18:0 and 20:0 (Fig. 5I). These findings indicate that GM3 species function as a rheostat for TLR4 signaling (Fig. 5J).

Remaining (previous) points:

1. Is the switch from VLCFA-GM3 to LCFA-GM3 in adipose tissue in obesity happening in adipocytes or linked to immune cell infiltration? The authors responded that, in general, mouse immune cells express complex gangliosides such as GM1 and GD1a although adipocytes express GM3 as predominant species [Ohashi M. Lipids 14, 52-57, 1979]. So, the switch of GM3 species might happen in adipocytes, and the increase of complex gangliosides (found slightly in EV.4F) would be due to immune cell infiltration as shown previously [Tanabe A. et al. Biochem Biophys Res Commun. 379, 547-52. 2009]. Here the authors hand-wave and fail to answer my question. I have looked at the Tanabe paper and the in vivo measurements are weak. It is clear that 3T3-L1 adipocytes have more GM3s whereas RAW macrophages have more GM1s and GM2s. So, in the end LC-MS measurement of the adipocyte vs macrophage fraction would corroborate the previous observations and settle this issue. This is also relevant for the overall concept: the authors ASSUME that the adipocytes change their GM3 composition due to HFD, from LCFA to VLCFA, and this impacts the signaling in the macrophage but there are no data showing this.
Answer: Our previous report suggested that GM3 expression in adipocytes was regulated by the co-presence of the resident macrophages in adipose tissue [Nagafuku et al., Glycobiology 2015; see Fig. 1I and K, Fig. 2G]. It has been also known that the activation of GM3 synthase in monocyte/macrophages was easily occurred during culturing in vitro [Gracheva et al., Biochemistry 72:772-777, 2007]. Therefore, except for the purpose of in vitro cell

culture/differentiation after the purification, in vitro cell fractionation would not be a suitable method for keeping the ganglioside levels of these cells and analyzing them in our purposes. In the near future, we would like to establish the specific method such as the imaging mass spectrometry, in order to detect GM3 species directly in the intact adipose tissues without in vitro cell manipulation as studied for other glycosphingolipid species [Sugimoto et al., PLoS One 11, e0152191. 2016].
So, according to your suggestion, we mentioned about the technical limitations mentioned above in the discussion part (page 17 to 18) as follows; "Moreover, it should be clarified directly in adipose tissue that GM3 species could mediate the adipocyte-macrophage communication in the future study. It would be important to specify the GM3 and other ganglioside species expressed in a specific type of cells, such as macrophages, pre-adipocytes, and differentiated adipocytes, that are mixed in adipose tissue. While pre-adipocytes/adipocytes predominantly express GM3, it is considered that human monocytes and mouse macrophages express GM3 and GM1/GD1a, respectively [60, 61]. However, it remains unclear how ganglioside species and their acyl-chain structures are different in a cell-type specific manner in the intact adipose tissue. To characterize miscellaneous cells in adipose tissue, in vitro enzymatic digestion/fractionation and antibody-based cell sorting are performed generally. On the other hand, our previous report suggested that GM3 expression in adipocytes was regulated by the co-presence of the resident macrophages in adipose tissue [17]. It has been also known that the activation of GM3 synthase in monocyte/macrophages was easily occurred during culturing in vitro [62]. Therefore, the specific method such as the imaging mass spectrometry for GM3 species should be established in order to analyze GM3 species directly in the intact adipose tissues without in vitro cell manipulation [63]."

2. In the new figure 7, the display of GM3 species as "Relative expression" is meaningless - wild-type and ob/ob mice obviously have very different adipose tissue. This is an important issue that usually arises when comparing lean and obese mice. The authors responded that, in general, the content of ganglioside in cell/ tissue can be normalized by the protein content in the same sample. I respectfully disagree. Adipose tissue in a lean mouse (per fat pad, e.g. epididymal) is comprised of mainly fat cells and low protein content per fat pad. In the obese roughly 90% of the cells are macrophages and the protein content is completely changed per g tissue and also per fat pad. So, the reasoning of the authors is flawed. Again, what is changed in the adipocyte fraction vs. the macrophages fraction would be most relevant. Is the change in GM3 an obesity-induced switch in the adipocyte or is it just more macrophages in the tissue. This is unclear.
Answer: As mentioned above, fractionation would not be a suitable method for keeping the ganglioside levels of these cells and analyzing them in our purposes. Moreover, even after the separation of floating adipocytes, collected SVF still contains not only macrophages but also many of pre-adipocytes (more than 60% in steady state), and small amounts of other immune cells and mesenchymal stem cells. Further fractionations by antibody-based separation will further increase the risk of affecting ganglioside levels. Therefore, it will be considerably difficult to estimate the accurate GM3 levels of adipocytes and macrophages in adipose tissue by separating these cells in vitro. In the future study, we will have to perform the imaging mass spectrometry to quantify GM3 species directly in these intact adipose tissues.
Regarding absolute quantification, we discussed the possibility as much as we could; however, we concluded there is a large difficulty to achieve it. To carry out the absolute quantification, isotope-labeled internal standards must be synthesized for every molecular species that could be detected by multiple reaction monitoring, and every molecular species should be quantified by every internal standards. However, it would be quite difficult or practically impossible to perform one-by-one quantification. Therefore, the quantification of glycosphingolipids and ceramides species by LC-MS/MS should be represented as the ratio to internal-standard and the relative expression level (fold to control) based on the signal intensity of every molecular species, that are normalized by the protein content of tissue (in lean and obese conditions), as reported elsewhere [Chavez JA. et al., JBC 289, 723-734 (2014) Fig.3A, 3D, 4A, and 5A; Turpin S.M. et al, Cell Metab. 20, 678–686 (2014) Fig.2A-D].

Referee #2:

I appreciate very much that the referee #2 has satisfied point 1 to 5.

© European Molecular Biology Organization

So, I am going to respond to point 6 and 7.

Point 1. In reference to Figure 1K it is stated that only GM3 species, but not Cer, GlcCer or LacCer display synergistic effects. While it is difficult to fully interpret the data as presented (in a heat map), it appears to me that GluCer and LacCer potentiate the effects of LPS. I would like to see this data presented in bar graph format so it is more interpretable as well as confirmation that these species do not display the same synergistic effects as GM3 species.

Answer: We refined the manuscript and figures, and showed these data also in bar graphs (in Fig. EV2 C-E). We added the description that monocyte activation was moderately enhanced in the presence of precursor GSL species, and reached to the maximum in the presence of GM3 24:0.

Reviewer Response: Satisfied with the response.

Point 2. In reference to Figure 2C and D, S100A9 has been implicated as a factor that is released from adipocytes and can activate TLR4, potentially contributing to metabolic inflammation. It would be interesting to see if the VLCFA-GM3 species also synergise with S100A9.
Answer: We are also interested in the functions of S100 proteins in chronic inflammation. In addition to S100A9, S100A8 also potentiates TLR4 activation in sepsis (ref No. 38), and recently, Vogl T. et al. reported that homotypic-/ heterotypic interactions between S100A8 and A9 proteins and binding to calcium ion could modulate their function as TLR4 ligands (J Clin Invest. 128,1852-1866. 2018). These S100A proteins could induce further expression of serum amyloid-A (SAA) proteins, which is involved in chronic inflammation in cancer metastasis (ref No. 39). SAA proteins are acute serum proteins increased in sepsis and also in obesity, and recently known as endogenous TLR2/4 ligands. Since GM3 species showed selectivity to TLR4, and to TLR2 partially, we have already started to investigate the functional interactions among GM3 species and endogenous TLR2/4 ligands, S100A and SAA proteins. However, to figure out the detail of molecular mechanism and target receptor selectivity, we would like to address these points more deeply in a future study.

Reviewer Response: The inclusion of some S100 experiments would have been nice, but it's not essential to the manuscript. So satisfied with the response.
Point 3. The inclusion of human clinical data together with the molecular work is a strength of the paper. With respect to the authors, I would consider re-ordering the current figures. The manuscript reads a little disjointed with the presentation of the clinical data in the middle of the manuscript. A better narrative might be achievable by having the clinical data at the beginning or end of the manuscript.

Answer: We agree the suggestion to re-order the current figures, putting the clinical data at the beginning of manuscript as Fig.1 and 2. Accordingly, we refined abstract describing the clinical data first followed by in vitro experiments, mouse experiments, and then docking model study.
Reviewer Response: Satisfied with the response.

Point 4. The authors state that an important aspect of the synergistic activation of TLR4 activation by GM3 is that it occurs at low concentrations of LPS, and a molecular rationale for this is discussed. Indeed, such an effect could be quite relevant in the context of metabolic inflammation where the levels of endogenous activators of TLR4, e.g. LPS/S100A9, are likely to be quite low relative to acute inflammatory conditions (e.g. sepsis). Potentiation of these activators by VLCFA-GM3 could therefore have a meaningful impact on TLR4 activation in conditions such as obesity-driven inflammation. However, the authors do not formally address the effective concentrations of LPS. In figure 1, concentrations of 0.13 and 0.06ng/ml of LPS are used. These are well below the typical concentrations typically used in studies of LPS (10ng/ml to 1ug/ml is typical in in vitro experiments). An experiment in which a fixed GM3 concentration used in combination with an extensive LPS dose response would be very insightful. Do you still see synergism at high doses of LPS? Or, is it possible, similar to LipidIVa, that at high doses of LPS even VLCFA-GM3 acts as an antagonist of TLR4 activation? These experiments would be quite straight-forward to conduct and hopefully would give some useful insight.

Answer: We added the full data covering wider range of LPS concentrations (in Fig. 4B, C and

Fig. EV2A). The synergy by VLCFA species is getting saturated in high concentration of LPS, which means that VLCFA species enhance low-grade activation of TLR4 without affecting maximal activation level. Fig. EV3 B-C also displayed the synergism in low Lipid-A concentration and the saturation in high Lipid-A concentration. On the other hand, the inhibition by LCFA/unsaturated species was observed still in high concentration of LPS (in Fig. 4B, C and Fig. EV2A).
As shown in several results, the optimal concentrations of LPS, that indicating dose-dependency, were different among cell types (human primary monocytes, < 0.5-1.0 ng/mL; mouse macrophages, < 1-2 ng/mL; transfected HEK293T cells, < 10-20 ng/mL).

Reviewer Response: Thank you for adding the new data. It's unfortunate that higher doses of LPS were not used (0.25ng/ml, the highest dose used, is still low compared with what it typically used), as this may have shown a true saturation of the effect, which you do not have. Nonetheless, the combinatorial effects of LPS + GM3 are clearly lowered at higher doses of LPS, as would be predicted. Satisfied with the response.

Point 5. The in vivo mouse studies presented in figures 6E and F do not implicate TLR4 directly, they only show that the loss of GM3s protects against obesity and impaired glucose tolerance. This may well be due to a TLR4-independent role of GM3s. I therefore feel that the implication of the title of the manuscript, i.e. that increased GM3 species exacerbate metabolic diseases by modulating TLR4, is not entirely proven based on the data presented. Indeed, mice are only being fed a high fat diet for 2 weeks. This is a very early time point in the evolution of metabolic dysfunction in rodents and, importantly, typically at this time little adipose tissue inflammation would be present. Therefore, the phenotype of the GM3 KO mice shown by the authors is not likely to be due to decreased TLR4 activation but some other effect of losing GM3s. If the authors were able to show reduced TLR4 activation in the high fat fed GM3 KO mice, this would greatly strengthen their case.

Answer: In the reference No.17 (Nagafuku et al., Glycobiology 25; 303-315, 2015), we have already reported that GM3S-KO attenuated the chronic-inflammatory phenotypes and metabolic abnormalities in HFD mice by 10-week feeding. In this study, we were also interested in the early phenotypes, because the VLCFA-shift in human serum GM3 occurred before the onset of chronic inflammation and metabolic disorders. So, we tried 2-week protocol, and confirmed at least that GM3S-KO could attenuate some of the early phenotypes as well as shown in long-term phenotypes. However, as pointed out in the comment above, it would be difficult to detect very small inflammatory symptoms in such a pathological stage. So, taken together, to improve the manuscript more understandable, we omitted the data according to discussions in the point 5. To investigate the TLR4 signaling in GM3S-KO adipose tissue, there are some technical limitations. High-fat-diet feeding induces chronic inflammation via TLR4 in visceral adipose tissue, but other signaling pathways are also activated simultaneously. On the other hand, LPS injection potentiates TLR4 signaling pathway selectively, but it induces strong (acute) systemic inflammation that could not be controlled as chronic adipose-tissue inflammation. Furthermore, to compare adipose-tissue inflammation via TLR4 (induced by LPS injection) between WT and GM3S-KO mouse, it would be better to newly establish the adipose-tissue cKO mouse to distinguish the local effect (by GM3 in visceral adipose tissue) and the systemic effect (by GM3 including other tissues). So, we would like to address these points more deeply in a future study.

Reviewer Response: I concur with reviewer 1 and the authors that removing this data is reasonable.

Point 6. With reference to Figure 7, the authors present evidence that key residues within TLR4, which mediate stability of the active heterotetrameric complex, are required for GM3's synergistic effects. While the cross-linking experiments provide evidence that GM3 is enhancing dimerization, this aspect of the work would be greatly enhanced by the analysis of real time dimerization of TLR4 following LPS stimulation in cells treated with or without GM3. This can be done by a flow cytometry based assay using a specific TLR4 antibody that discriminates between active and non-active TLR4 (used extensively by the Kagan lab, see for example Zanoni et al 2015). This would provide more compelling evidence that GM3 is augmenting TLR4 dimerisation (and also endocytosis, a key component of TLR4 signalling) than the cross-linking assay.

Answer: In general, the FACS analyses using mAb MTS510 (specific to surface TLR4/MD-2 monomer) and mAb Sa15-21 (pan detection of surface TLR4) are effective to investigate LPS-mediated TLR4 internalization and to estimate the dimerization. On the other hand, these methods are established on the basis of LPS-mediated signaling events in mouse immune cells. High LPS concentration is required for starting activation of almost all cell-surface TLR4 simultaneously (in a short time), rather than inducing chronic activation of TLR4 persistently (for a long time). So, it is ambiguous whether these methods are applicable to analysis for chronic signaling events mediated by endogenous ligands or low concentration LPS. Therefore, in a future study, we would like to clarify whether the FACS analysis is effective for chronic TLR4 activation and also for the synergistic signaling event by VLCFA GM3 species.

Reviewer Response: The authors are correct in stating that previous studies that have used this approach to assess TLR4 activation have typically used high concentrations of LPS. They also make a point about acute vs chronic stimulation that may be valid. However, these experiments are not challenging to conduct and I feel they should be performed. I would probably try co-treatments with LPS and GM3, as well as a more prolonged pre-treatment with GM3 (perhaps addressing the authors' comments about chronic stimulation), followed by LPS stimulation. I think these experiments are very worthwhile to conduct. If indeed they prove to be technically unfeasible due to the relatively low concentrations of the agonists being used (compared to the high concentrations of LPS typically used in these assays), then so be it. But I would comment that you are seeing a potentiation of TLR4-dependent increases in cytokine production, and so, according to the hypothesis being proposed by the authors, TLR4 dimerization/internalization should be detectable by these methods - which are very sensitive. The authors have not been asked for extensive experimental additions, so it's not unreasonable to expect that those requests for additional work, where they have been made, and which are technically quite straight forward, be attempted.

Answer:
We tried flow-cytometric analysis of mTLR4/MD-2 using MTS510 mAb which can detect both dimerization and internalization. According to the established FACS methods [Akashi S. et al., JEM (2003), Zanoni I. et al., Cell (2011), Tan Y. et al., Immunity (2015)], 1-3 micro g/mL LPS is required for inducing dimerization, and the level of dimerization was almost saturated at around 60-80% within 30-60 min. On the other hand, our ELISA data indicated that low-dose LPS (1-10 nano g/mL), 100- or 1000-fold less than optimal concentration for flow cytometry, could successfully show the synergism with GM3 species on cytokine production. So, we used a wide range of low-dose LPS (0.5, 5, 50, and 500 ng/mL) and stimulated RAW macrophages for 60 min. We also added VLCFA-GM3 22:0 (10, 25 μM) to see the synergism.
Our results showed that monomeric/surface mTLR4/MD-2 population could decrease in a dose-dependent manner (Panel 1, red arrow), but the response was weak (Graph 1 and Panel 2). Compered to control, the shift was 20% in 500 ng/mL LPS, less than 10% in 50 ng/mL LPS, and less than only 5% in 5 and 0.5 ng/mL LPS (Graph 1), the concentrations used for cytokine production. It suggested that only a small population of TLR4/MD-2 was activated in the presence of low concentration LPS, and the sensitivity of flow-cytometric analysis was not sufficient. We also assessed the effect of GM3 22:0, but the change was very small due to the insufficient dynamic range for weak activation level (Panel 2, and Graph 1).
We also analyzed the time course of cytokine production using low-dose of LPS (0, 5, 50 ng/mL) plus GM3 22:0 (5 μM) at three time points (at 3, 9, and 18 hr) (Graph 2). Lower-dose LPS (5 ng/mL) induced chronic/weak TNF-α production throughout the incubation, and the synergistic effect of GM3 22:0 was observed clearly. Higher-dose LPS (50 ng/mL) induced rapid/strong activation, but the synergistic effect of GM3 22:0 was very small due to the early saturation by higher amount of LPS (Graph 2).
These results indicated that the optimal dose of LPS differs largely between flow-cytometric analysis and cytokine production, and it is difficult to mimic chronic signaling events by using high concentration of LPS. Chronic phenotypes might be induced by long-term induction of weak signaling that can be detected as accumulations of cytokines or reporter products. So, we feel that further optimization and modification are required for flow-cytometric analysis of chronic TLR4 activation, otherwise, some different types of analyses such as living-cell imaging might enable us to observe small population of TLR4/MD-2 activated by low-dose LPS and endogenous ligands. We would like to perform the further optimization and the specific

modification for the methods in the future study. We described the effectiveness and possibility of flow-cytometric analysis in the discussion part (page 16 to 17) as follows: " Additionally, it is known that the dimerization and internalization of mTLR4/MD-2 upon acute stimulation by LPS can be analyzed by flow cytometry [53, 54, 55], which might enable to detect GM3-mediated receptor dynamics directly on the plasma membrane of living cells."

Point 7. Finally, no signaling data is presented in the manuscript. I feel it is important that such data be added to give a more complete picture of the mechanistic basis by which VLCFA-GM3 is augmenting LPS responses. Time course pathway analysis, by for example immunoblotting, would add another level of mechanistic insight to the manuscript. With this and the other above experiments I have mentioned, the manuscript would provide and very comprehensive basis by which GM3s augment TLR4 signaling.

Answer: We tried to investigate the activation of several signaling pathways downstream of TLR4. We succeeded to detect and compare the activities of NF-κB response element, AP-1 response element (downstream of MAPK), and interferon-stimulated response element (ISRE) (downstream of IRF3/7 or feedback from type-I interferon production), and the results were shown in Fig. EV3 E. NF-κB response element activity was most strongly activated by LPS and GM3 22:0. Similarly, AP-1 response element activity was moderately activated, suggesting that GM3-mediated enhancement of TLR4 signaling is dependent on NF-κB and MAPK signaling pathway. On the other hand, ISRE activity was enhanced only weakly, implying that the contribution of the IRF3/7 pathway is relatively small. In general, relatively strong LPS stimulation is required for analyzing protein phosphorylation (e.g. phospho-I-kappaB, phospho-MAPK) by western blotting. So, we chose the reporter assay that is applicable to chronic stimulation.

Reviewer Response: My feeling with regards to this point is somewhat to similar to that stated above. The reporter assays do not adequately address the point I raised. I would like to see whether signaling kinetics are altered. This type of analysis is very standard when assessing receptor activation. As I suggested above, perhaps the authors could try co-treatments with GM3 and LPS and look at acute (5 min to 4hrs) signaling pathways activation, as well as a chronic gm3 treatment followed by LPS acutely. Stating that relatively strong LPS stimulation is required for analyzing protein phospho changes by immunoblotting is not accurate. The experiments should at least be attempted. If they are successful I think they add considerable insight.

Answer: According to references for flow-cytometric analysis, western blot analysis also requires the same LPS dose (1 μg/mL) as that of flow-cytometric analysis. With consideration of results of flow-cytometric analysis, we concluded that these methods would not be optimal for analyzing the kinetics of chronic TLR4 activation by low concentration of LPS and GM3. On the other hand, however, we agreed with your suggestion about the importance of the kinetic analysis. So, we added the Graph 2 (time course of cytokine production) into Main figure 6 (as 6B) to show the kinetics of GM3-mediated cell activation together with the explanation that "The enhancement was clearly observed in chronic/weak TLR4 activation by low-dose LPS, and was saturated in rapid/strong activation by high-dose LPS" (Page 10). In future study, it would be important to establish the kinetic analysis sensitive enough to detect weak signaling events, such as real-time monitoring of NF-kB activity in living cells, based on more sensitive/bright luciferase strains with in vivo substrates.

Panel 1. FACS analysis of monomeric/suraface mTLR4/MD-2 population (responses to LPS).

FACS by anti-mTLR4/MD-2 mAb (MTS510)

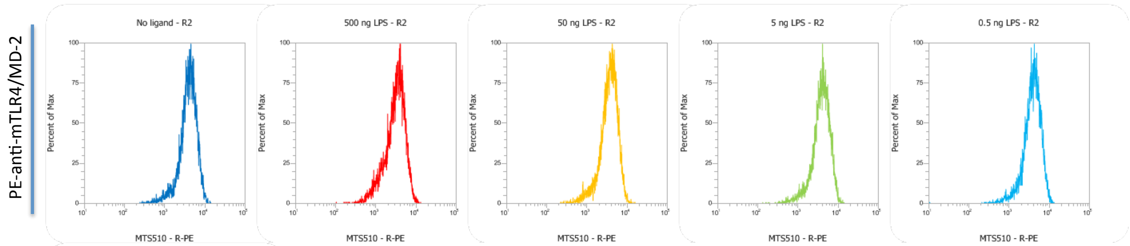

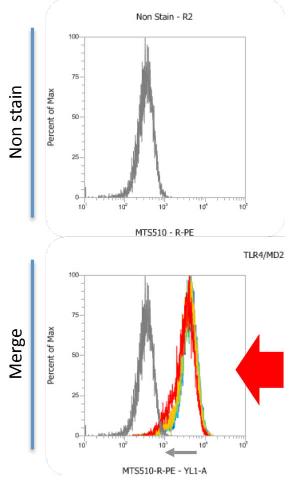

<Protocols>
RAW cells:
Cultured in 1.0 % FCS-DMEM overnight.
Resuspended in conditioned medium with 10 mM HEPES pH7.0.
Approx. 2x10e5 cells/ 100 µL in 1.5 mL tube.

Stimulation:
0.5, 5, 50 ,500 ng/mL LPS,
and 10, 25 µM GM3 22:0.

Incubation: 1h, at 37°C.

Antibodies and staining:
Treated with 5 µL mouse Fc-Blocker (Miltenyi) in 0.1% FCS-PBS for 10 min, and
stained with 0.5 µL PE-anti-TLR4/MD-2(MTS510)(Biolegend) for 20-30min on ice.
Then washed in 0.1% FCS-PBS.

Cell analyzer:
Attune NxT (Thermo scientific) with yellow laser for R-PE (Filter: 585/16 nm).

Panel 2. FACS analysis of monomeric/suraface mTLR4/MD-2 population (responses to GM3)

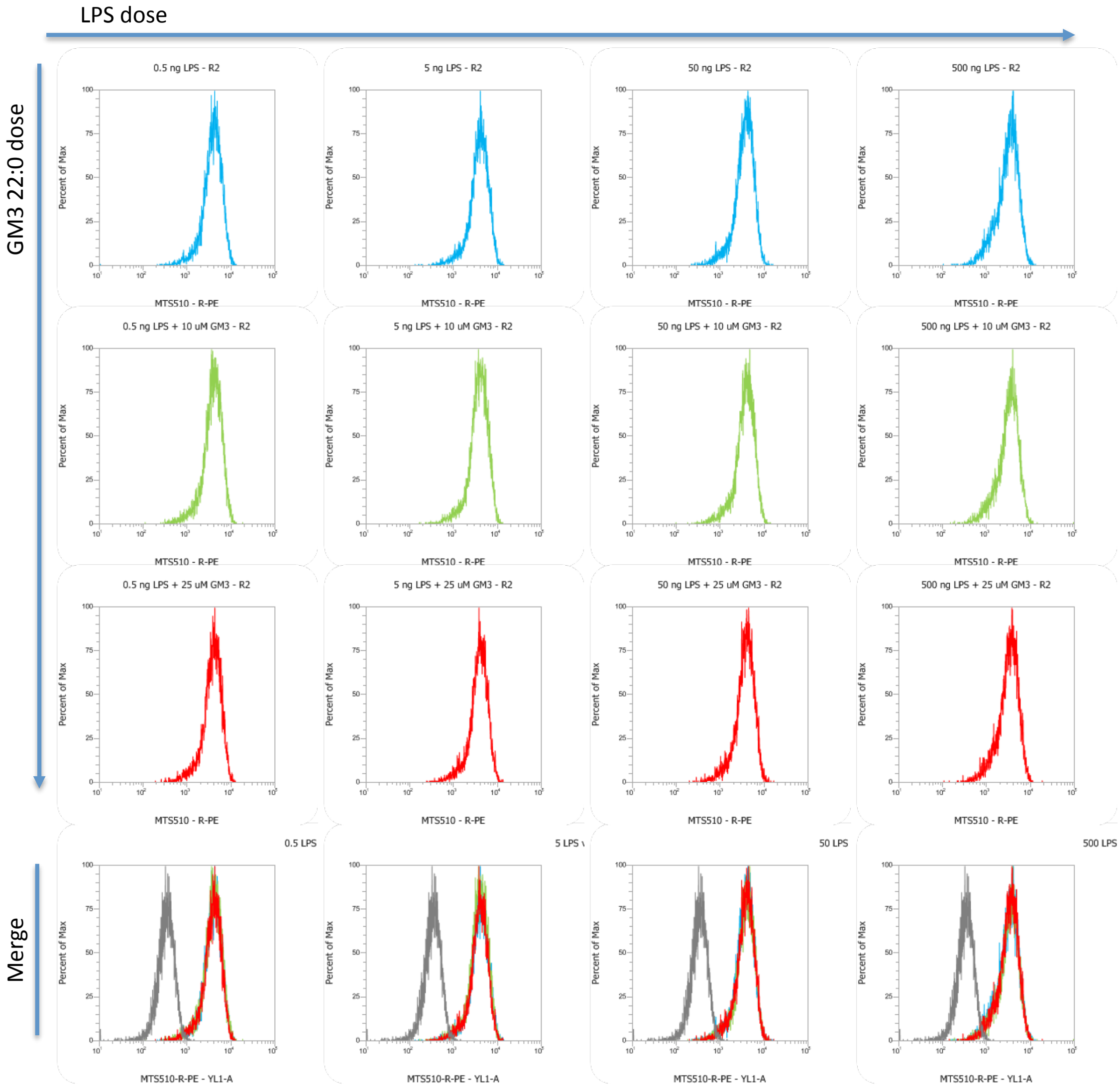

(Histograms of controls, stimulations only by LPS, are same results of Panel 1.)

Graph 1. Quantification of the effect of LPS and GM3 22:0 by FACS analysis.

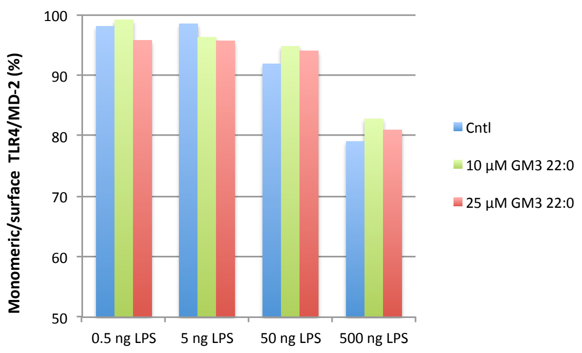

Monomeric/surface population of mTLR4/MD-2 was quantified based on MFI.

Graph 2. Time course of TNF-α production by lower and higher concentration of LPS plus GM3.

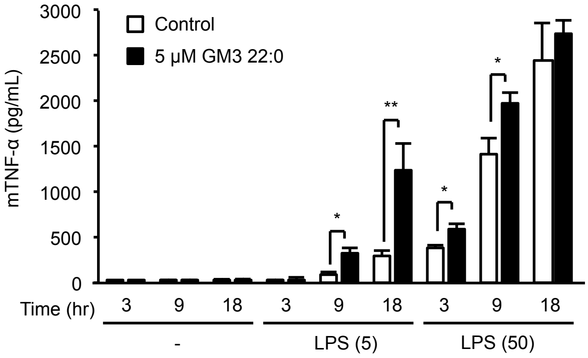

Co-stimulation of RAW macrophages by lower-/ higher concentration of LPS (0, 5, 50 ng/mL) plus GM3 22:0 (5 μM). Time course of TNF-α production in culture supernatant was quantified by ELISA.

3rd Editorial Decision 24 February 2020

Thank you for submitting your revised manuscript to The EMBO Journal. Your study has now been re-evaluated by the referees and as you can see from the comments below, they are happy with the introduced revisions.

I am therefore pleased to let you know that we will accept the manuscript for publication here. Before sending you the formal accept letter there are just a few editorial things to sort out

- It would be good to deposit the mass spec data (on the GM3 species) in a suitable database and provide the accession number(s) in the manuscript as part of the Data Availability Section

- Author contribution is missing for Wataru Nihei.

- Please remove the Figure legends from the individual Figure and EV Figure files and add them after the reference list.

- 'Author name order:' in the main MS listed before the Author Acknowledgments should be removed

- Reference style should be alphabetical.

- Please merge your 5 Appendix figures and create a Table of Contents into one appendix file. The Appendix figure legends should be removed from the main MS and added to the appendix.

- I have asked our publisher to do their pre-publication checks on the paper. They will send me the file within the next few days. Please wait to upload the revised version until you have received their comments.

That should be all let me know if we need to discuss anything further

----

REFEREE REPORTS

Referee #1:

The authors have addressed my concern by elaborating on their limitations in the discussion. I agree that another study would be needed to overcome the technical task of identifying the exact source of GM3s in obesity, especially in adipose tissue. This study has built a strong foundation for any future work. I think the paper is very complex, rich in data and implications, and some of these key messages could be emphasized in a comment accompanying this work. I have no further concerns.

Referee #2:

The authors have done a much more thorough job of addressing the issues I raised. I thank them for their efforts with regards to the flow cytometric assay and agree with their interpretation. I'm satisfied with their response.

With regards to the signaling kinetics experiments I raised, I don't completely agree that high concentrations of LPS are require to detect signaling changes by immunoblotting. The inclusion of the cytokine secretion time course is welcome though. I'm satisfied with the response though.

All of my previous comments were satisfied after the initial review.

3rd Revision - authors' response 13 March 2020

The authors performed all minor editorial changes.

EMBO PRESS

YOU MUST COMPLETE ALL CELLS WITH A PINK BACKGROUND ↓

PLEASE NOTE THAT THIS CHECKLIST WILL BE PUBLISHED ALONGSIDE YOUR PAPER

Corresponding Author Name: Jin-ichi Inokuchi

Journal Submitted to: The EMBO Journal
Manuscript Number: EMBOJ-2019-101732R1

USEFUL LINKS FOR COMPLETING THIS FORM

http://www.antibodypedia.com
http://1degreebio.org
http://www.equator-network.org/reporting-guidelines/improving-bioscience-research-repor
http://grants.nih.gov/grants/olaw/olaw.htm
http://www.mrc.ac.uk/Ourresearch/Ethicsresearchguidance/Useofanimals/index.htm
http://ClinicalTrials.gov
http://www.consort-statement.org
http://www.consort-statement.org/checklists/view/32-consort/66-title
http://www.equator-network.org/reporting-guidelines/reporting-recommendations-for-tumo
http://datadryad.org
http://figshare.com
http://www.ncbi.nlm.nih.gov/gap
http://www.ebi.ac.uk/ega
http://biomodels.net/
http://biomodels.net/miriam/
http://jjj.biochem.sun.ac.za
http://oba.od.nih.gov/biosecurity/biosecurity_documents.html
http://www.selectagents.gov/

Reporting Checklist For Life Sciences Articles (Rev. June 2017)

This checklist is used to ensure good reporting standards and to improve the reproducibility of published results. These guidelines are consistent with the Principles and Guidelines for Reporting Preclinical Research issued by the NIH in 2014. Please follow the journal's authorship guidelines in preparing your manuscript.

A- Figures
1. Data
The data shown in figures should satisfy the following conditions:

- the data were obtained and processed according to the field's best practice and are presented to reflect the results of the experiments in an accurate and unbiased manner.
- figure panels include only data points, measurements or observations that can be compared to each other in a scientifically meaningful way.
- graphs include clearly labeled error bars for independent experiments and sample sizes. Unless justified, error bars should not be shown for technical replicates.
- if n< 5, the individual data points from each experiment should be plotted and any statistical test employed should be justified
- Source Data should be included to report the data underlying graphs. Please follow the guidelines set out in the author ship guidelines on Data Presentation.

2. Captions

Each figure caption should contain the following information, for each panel where they are relevant:

- a specification of the experimental system investigated (eg cell line, species name).
- the assay(s) and method(s) used to carry out the reported observations and measurements
- an explicit mention of the biological and chemical entity(ies) that are being measured.
- an explicit mention of the biological and chemical entity(ies) that are altered/varied/perturbed in a controlled manner.

- the exact sample size (n) for each experimental group/condition, given as a number, not a range;
- a description of the sample collection allowing the reader to understand whether the samples represent technical or biological replicates (including how many animals, litters, cultures, etc.).
- a statement of how many times the experiment shown was independently replicated in the laboratory.
- definitions of statistical methods and measures:
  - common tests, such as t-test (please specify whether paired vs. unpaired), simple χ2 tests, Wilcoxon and Mann-Whitney tests, can be unambiguously identified by name only, but more complex techniques should be described in the methods section;
  - are tests one-sided or two-sided?
  - are there adjustments for multiple comparisons?
  - exact statistical test results, e.g., P values = x but not P values < x;
  - definition of 'center values' as median or average;
  - definition of error bars as s.d. or s.e.m.

Any descriptions too long for the figure legend should be included in the methods section and/or with the source data.

In the pink boxes below, please ensure that the answers to the following questions are reported in the manuscript itself. Every question should be answered. If the question is not relevant to your research, please write NA (non applicable). We encourage you to include a specific subsection in the methods section for statistics, reagents, animal models and human subjects.

B- Statistics and general methods

Please fill out these boxes ↓ (Do not worry if you cannot see all your text once you press return)

1.a. How was the sample size chosen to ensure adequate power to detect a pre-specified effect size?	We determined the sample size according to previous studies (reference#13, 16, 17).
1.b. For animal studies, include a statement about sample size estimate even if no statistical methods were used.	No statistical method was used for sample size estimation.
2. Describe inclusion/exclusion criteria if samples or animals were excluded from the analysis. Were the criteria pre-established?	Tukey's fense method was applied.
3. Were any steps taken to minimize the effects of subjective bias when allocating animals/samples to treatment (e.g. randomization procedure)? If yes, please describe.	Mice were randomly divided into 2 groups, and then subjected to normal-diet and high-fat diet feeding.
For animal studies, include a statement about randomization even if no randomization was used.	We described the point above in the materials and methods part.
4.a. Were any steps taken to minimize the effects of subjective bias during group allocation or/and when assessing results (e.g. blinding of the investigator)? If yes please describe.	For human serum analyses, the collection of serum, the clinical marker acquisition, the LC/MS data acquisition, and the correlational analyses were performed by different researchers and partially blinded. Analyses by artificial intelligence was also used in order to reduce the subjective bias.
4.b. For animal studies, include a statement about blinding even if no blinding was done	No blinding was allpied between feeding and investigating procedures.
5. For every figure, are statistical tests justified as appropriate?	We chose appropriate methods as far as possible. Multiple comparison: Tukey's multiple comparison (honesty significant difference) test or two-tailed unpaired t-test (with modification for unequal variances; Welch's t-test) with Bonferroni's correction was used. Two-group comparison: two-tailed unpaired t-test (with modification for unequal variances; Welch's t-test) was used.
Do the data meet the assumptions of the tests (e.g., normal distribution)? Describe any methods used to assess it.	No test was used for assessing normal distribution.
Is there an estimate of variation within each group of data?	We indicated the standard deviation for every group of data.

Is the variance similar between the groups that are being statistically compared?	Two-tailed unpaired t-test with modification for unequal variances (Welch's t-test) was applied.

C- Reagents

6. To show that antibodies were profiled for use in the system under study (assay and species), provide a citation, catalog number and/or clone number, supplementary information or reference to an antibody validation profile. e.g., Antibodypedia (see link list at top right), 1DegreeBio (see link list at top right).	This study does not contain the data for western blotting or immunohistochemistry. For ELISA, all antibodies are included in the ELISA kits from Biolegend indicated in the method section.
7. Identify the source of cell lines and report if they were recently authenticated (e.g., by STR profiling) and tested for mycoplasma contamination.	Purchased cell-lines: HEK293T, RIKEN; RAW264.7, ATCC. The elimination of Mycoplasma comtamination was rouitinely performed by addition of killing/preventing concentrations of Plasmocin, an anti-Mycoplasma macrolide (Invivogen).

* for all hyperlinks, please see the table at the top right of the document

D- Animal Models

8. Report species, strain, gender, age of animals and genetic modification status where applicable. Please detail housing and husbandry conditions and the source of animals.	Information of mouse strain, gender, and age were desribed in methods. Mice were purchased from CLEA Japan and Japan SLC, and the cages were placed in a room with controlled temperature (21–23°C) and lighting (07:00–19:00).
9. For experiments involving live vertebrates, include a statement of compliance with ethical regulations and identify the committee(s) approving the experiments.	All animals were maintained in accordance with Tohoku Medical and Pharmaceutical University guidelines for the care and use of laboratory animals by the ethics committee for the animal study of Tohoku Medical and Pharmaceutical University.
10. We recommend consulting the ARRIVE guidelines (see link list at top right) (PLoS Biol. 8(6), e1000412, 2010) to ensure that other relevant aspects of animal studies are adequately reported. See author guidelines, under 'Reporting Guidelines'. See also: NIH (see link list at top right) and MRC (see link list at top right) recommendations. Please confirm compliance.	We read those guidelines and confirmed compliance for animal studies.

E- Human Subjects

11. Identify the committee(s) approving the study protocol.	Our studies were approved by the ethics committee of Tohoku Medical and Pharmaceutical University and of University of Tokyo.
12. Include a statement confirming that informed consent was obtained from all subjects and that the experiments conformed to the principles set out in the WMA Declaration of Helsinki and the Department of Health and Human Services Belmont Report.	We confirmed the informed consent of all subjects and the research procedures according to these declarations and principles.
13. For publication of patient photos, include a statement confirming that consent to publish was obtained.	Not applicable.
14. Report any restrictions on the availability (and/or on the use) of human data or samples.	Human sera are not available. Data are partially available on request.
15. Report the clinical trial registration number (at ClinicalTrials.gov or equivalent), where applicable.	Not applicable.
16. For phase II and III randomized controlled trials, please refer to the CONSORT flow diagram (see link list at top right) and submit the CONSORT checklist (see link list at top right) with your submission. See author guidelines, under 'Reporting Guidelines'. Please confirm you have submitted this list.	Not applicable.
17. For tumor marker prognostic studies, we recommend that you follow the REMARK reporting guidelines (see link list at top right). See author guidelines, under 'Reporting Guidelines'. Please confirm you have followed these guidelines.	Not applicable.

F- Data Accessibility

18: Provide a "Data Availability" section at the end of the Materials & Methods, listing the accession codes for data generated in this study and deposited in a public database (e.g. RNA-Seq data: Gene Expression Omnibus GSE39462, Proteomics data: PRIDE PXD000208 etc.) Please refer to our author guidelines for 'Data Deposition'.<br><br>Data deposition in a public repository is mandatory for:<br>a. Protein, DNA and RNA sequences<br>b. Macromolecular structures<br>c. Crystallographic data for small molecules<br>d. Functional genomics data<br>e. Proteomics and molecular interactions	This study does not include large-scale datasets of RNA sequencing, microarray, proteomics, crystallography, functional genomics, or any sequence data related to these analyses.
19. Deposition is strongly recommended for any datasets that are central and integral to the study; please consider the journal's data policy. If no structured public repository exists for a given data type, we encourage the provision of datasets in the manuscript as a Supplementary Document (see author guidelines under 'Expanded View' or in unstructured repositories such as Dryad (see link list at top right) or Figshare (see link list at top right).	This study does not include large-scale datasets of RNA sequencing, microarray, proteomics, crystallography, functional genomics, or any sequence data related to these analyses. For the correlational study of GM3 species and clinical markers, we showed the data of every GM3 species and clinical markers in the supplemetary part.
20. Access to human clinical and genomic datasets should be provided with as few restrictions as possible while respecting ethical obligations to the patients and relevant medical and legal issues. If practically possible and compatible with the individual consent agreement used in the study, such data should be deposited in one of the major public access-controlled repositories such as dbGAP (see link list at top right) or EGA (see link list at top right).	This study does not include any practical clinical studies or human genomics datasets.
21. Computational models that are central and integral to a study should be shared without restrictions and provided in a machine-readable form. The relevant accession numbers or links should be provided. When possible, standardized format (SBML, CellML) should be used instead of scripts (e.g. MATLAB). Authors are strongly encouraged to follow the MIRIAM guidelines (see link list at top right) and deposit their model in a public database such as Biomodels (see link list at top right) or JWS Online (see link list at top right). If computer source code is provided with the paper, it should be deposited in a public repository or included in supplementary information.	Computational analysis for GM3 species was perfomed on the public software packages sited in reference #69. SOM: Kohonen's group of Helsinki University of Technology, (http://www.cis.hut.fi/research/som-research/nnrc-programs.shtml). BRNN: Flexible Bayesian Modeling, (http://www.cs.toronto.edu/ radford/fbm.2004-11-10.doc/index.html). For ligand-macromolecular docking, the calculation was performed on the public software Avogadro (https://avogadro.cc) and Autodock 4.2 (http://autodock.scripps.edu).

G- Dual use research of concern

22. Could your study fall under dual use research restrictions? Please check biosecurity documents (see link list at top right) and list of select agents and toxins (APHIS/CDC) (see link list at top right). According to our biosecurity guidelines, provide a statement only if it could.	Not applicable.
